# The mosaicism of plasmids revealed by atypical genes detection and analysis

**DOI:** 10.1186/1471-2164-12-403

**Published:** 2011-08-08

**Authors:** Emanuele Bosi, Renato Fani, Marco Fondi

**Affiliations:** 1 Lab. of Microbial and Molecular Evolution, Dept. of Evolutionary Biology, Via Romana 17-19, University of Florence, Italy

## Abstract

**Background:**

From an evolutionary viewpoint, prokaryotic genomes are extremely plastic and dynamic, since large amounts of genetic material are continuously added and/or lost through promiscuous gene exchange. In this picture, plasmids play a key role, since they can be transferred between different cells and, through genetic rearrangement(s), undergo gene(s) load, leading, in turn, to the appearance of important metabolic innovations that might be relevant for cell life. Despite their central position in bacterial evolution, a massive analysis of newly acquired functional blocks [likely the result of horizontal gene transfer (HGT) events] residing on plasmids is still missing.

**Results:**

We have developed a computational, composition-based, pipeline to scan almost 2000 plasmids for genes that differ significantly from their hosting molecule. Plasmids atypical genes (PAGs) were about 6% of the total plasmids ORFs and, on average, each plasmid possessed 4.4 atypical genes. Nevertheless, conjugative plasmids were shown to possess an amount of atypical genes than that found in not mobilizable plasmids, providing strong support for the central role suggested for conjugative plasmids in the context of HGT. Part of the retrieved PAGs are organized into (mainly short) clusters and are involved in important biological processes (detoxification, antibiotic resistance, virulence), revealing the importance of HGT in the spreading of metabolic pathways within the whole microbial community. Lastly, our analysis revealed that PAGs mainly derive from other plasmid (rather than coming from phages and/or chromosomes), suggesting that plasmid-plasmid DNA exchange might be the primary source of metabolic innovations in this class of mobile genetic elements.

**Conclusions:**

In this work we have performed the first large scale analysis of atypical genes that reside on plasmid molecules to date. Our findings on PAGs function, organization, distribution and spreading reveal the importance of plasmids-mediated HGT within the complex bacterial evolutionary network and in the dissemination of important biological traits.

## Background

Comparative whole-genome analyses have demonstrated that horizontal gene transfer (HGT) provides a significant contribution to prokaryotic genome evolution/innovation. In fact, it is very likely that a significant proportion of the genetic diversity exhibited by extant bacteria might be the result of the acquisition of sequences from more or less distantly related organisms [1]. Indeed, HGT gives a venue for bacterial diversification by the reassortment of existing capabilities [1] and this formidable sexual promiscuity has given bacteria a great advantage, providing an awesome mechanism for ongoing adaptive evolution, a sort of permanently and rapidly evolving communal genome [2].

During evolution, HGT and recombination have shaped bacterial genomes, which today appear as complex mosaics of genes from different lineages, species, and genera [2].

In this picture, plasmids (collections of functional genetic modules that are organized into a stable, self-replicating entity or 'replicon'), might have played (and might still play) a major role because they can be transferred between microorganisms, thus representing natural vectors for the transfer of genes and the functions they code for [3]. Moreover, it can be suggested that, during their evolutionary history, plasmids can undergo genetic rearrangements with either plasmids and/or cromosome(s) residing the same cytoplasm and/or with phages infecting the same cell. As a consequence, newly acquired genes can be integrated on plasmids and (eventually) be maintained.

In general, three major processes can mediate HGT among bacteria: transformation (the uptake of free DNA), transduction (DNA transfer mediated by bacteriophages) and conjugation (DNA transfer by means of plasmids or integrative conjugative elements) [4]. However, regardless of the transfer mechanism, once that DNA has entered the recipient cell it can undergo homologous recombination or homology-facilitated illegitimate recombination and can be successfully integrated into the genome of the new host. Lastly, if the newly acquired DNA confers a selective advantage to the host, it can be maintained and, possibly, spread again through the bacterial population. Importantly, it can be surmised that, at least in the first stages following the integration event (before the amelioration process can start), exogenous sequences maintain their own peculiar compositional features [e.g. GC% and dinucleotide relative abundance difference (δ*)] that usually differ from the rest of the "new" hosting molecule; for this reason these sequences are often defined "atypical".

Atypical (and, possibly, horizontally transferred) genes detection can be pursued by composition-based methods [5,6] that involve alignment-free features, such as GC% content and/or δ* [6]. Compositionally oriented methods rely on the observation that some genome features (including GC% content and δ*) are typical for a given bacterial genome and similar between closely related genomes. Accordingly, recently acquired genes are likely to display anomalous composition, especially when they originated in distantly related species; moreover a different composition will also be observed in those cases in which amelioration process has been retarded. Interestingly, it has been proposed that the genome signature (a compositional parameter reflecting the dinucleotide relative abundance values between two different DNA strands) of plasmids does not resemble that of their host genome, probably indicating either absence of amelioration or a less stable relationship between plasmids and their host [7].

Based on composition-oriented strategies, recent analyses on large sets of bacterial and archaeal chromosomes have revealed their mosaic structure, since considerable proportions of most of them consist of horizontally acquired genes [8-10]. For example, applying a Bayesian method on 116 prokaryotic complete genomes, Nakamura et al (2004) found that the average proportion of horizontally transferred genes *per *genome was about 12% of all ORFs, ranging from 0.5% to 25%. Similarly Cortez et al. (2009), analysing a set of 119 bacterial and archaeal chromosomes (351111 ORFs), found that a large fraction of them was populated by atypical genes (defined as clusters of atypical genes, CAGs) (58487, 16% of all genes). Hence, this strongly indicates that archaeal and bacterial chromosomes contain an impressive proportion of recently acquired foreign genes (including ORFans, that is open reading frames without matches in current sequence databases) coming from a still largely unexplored reservoirs [10]. Finally, the same authors found that among the identified CAGs, a large number were likely of plasmid origin [10]. These lines of evidence suggest that genetic mobility should not be merely interpreted in terms of transportation of genes bypassing the cell barriers of prokaryotes, but rather as a perpetual flow between discrete reproductive units [11], i.e chromosomes and/or MGE, including plasmids. In fact, an emerging view suggests that plasmids (and MGEs in general) should be considered as mosaics of functional blocks (modules) of genes [12,13]. Remarkably, in a few cases, the mosaic structure of plasmids (according to compositional criteria) has been demonstrated [9,14,15] revealing interesting insights on the dissemination of key biological traits such as antibiotic resistance, virulence and heavy metal detoxification. Accordingly, it is reasonable that the identification and the analysis of plasmid atypical genes (hereinafter PAGs) might reveal interesting insights in the (probably complex) network of intra- and inter-cellular gene transfer(s) that plasmids can face during their evolution. Indeed, PAGs might be either the outcome of (one or more) HGT(s) or of internal recombination events with chromosome(s) residing the same cytoplasm but possessing different compositional signatures.

However, to the best of our knowledge, a massive analysis of alien modules that may reside on plasmid molecules has not been undertaken up to now. Therefore, the aim of this work was to develop a statistically validated computational strategy that integrates two distinct compositional measures [GC% content and δ*] to scan nearly 2000 archaeal and bacterial plasmids for the presence of PAGs. Finally retrieved PAGs datasets have been analyzed, revealing interesting trends in the overall plasmid gene exchange network.

## Methods

### Analyzed genomes

All the available complete plasmids, phages and chromosome sequences were downloaded from NCBI ftp site at (http://www.ncbi.nlm.nih.gov/Ftp/, as on February the 1^st ^2010). Concerning plasmids, we focused our attention only on those longer than 3 kb and harboring at least 2 ORFs, in order to be able to detect δ* and differences in GC% content among all the genes. This allowed to assemble a dataset of 1853 plasmids for a total of 128.569 ORFs. The complete list of plasmids analyzed in this work (together with other information such as their size, their accession codes etc.) is available as Additional file 1.

### Atypical genes detection

**Figure 1:**
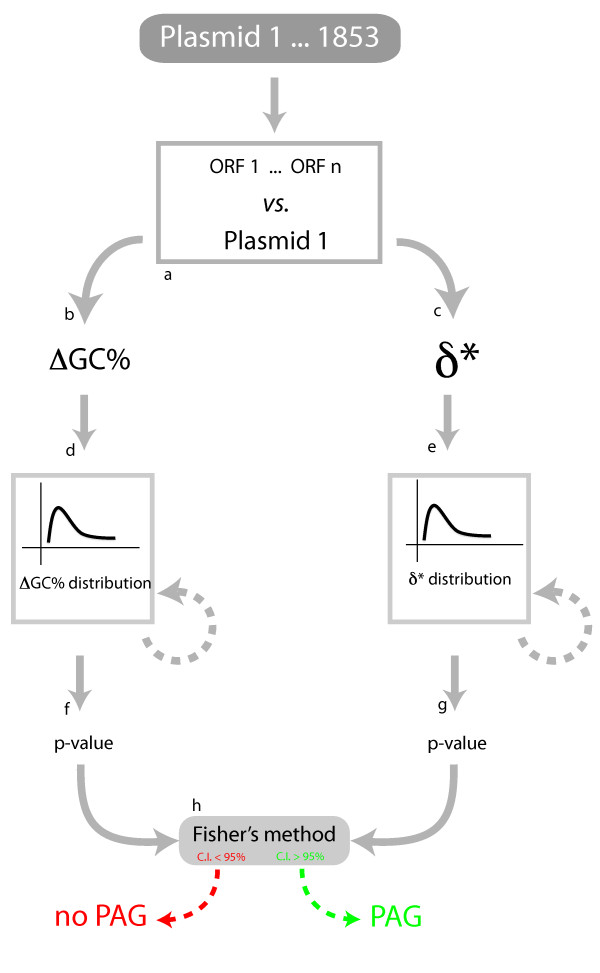
**Applied strategy**. The overall strategy adopted in this work for the identification of plasmids atypical genes (PAGs).

Classically, atypical genes detection has been pursued either by i) phylogenetic methods based on sequence alignment) and/or ii) by composition-based methods that involve alignment-free features such as GC% content, synonymous codon usage or the frequencies of overlapping short oligomers [16]. Several *in silico *based methods have been conceived in the past few years to identify foreign genes that were recently acquired by chromosomes [1,17-25]. However, it is not possible to discriminate between plasmids atypical or native genes using phylogeny-oriented methods since a set of homologous and universally shared sequences (necessary to build a reference phylogeny) are often unavailable. Furthermore, the absence of a universally shared "core" of genes (i.e. likely not subject to extensive HGT) when analysing plasmids sequences does not allow the use of classical statistics methods (such as Markov model-based approaches), which require the presence of a set of native genes in order to identify those ORFs that have a signifcant different composition. Hence, in this work, we have developed a computational strategy (Figure 1) that combines two compositional measures (GC content difference and δ*) in order to identify putative PAGs within a dataset of 1853 plasmids. Briefly, GC content is calculated as (G+C)/(A+T+G+C), where G, C, A and T is the number of guanines, cytosine, adenines and thymines, respectively. Conversely, δ* between two sequences a and b (from different organisms or from different regions of the same genome) is calculated as δ^∗^(a,b) = (1/16)∑_XY_|ρ*_XY_(a)−ρ*_XY_(b)| where the sum extends over all possible XY dinucleotides and where ρ_XY _= f_XY _/f_X_f_Y_, with f_XY _representing the frequency of the dinucleotide XY in the genome.

Hence, for each of the 128.569 ORFs of the 1853 plasmids (Figure 1a), we estimated both the δ* and the GC content difference (ΔGC) in respect to the corresponding source plasmid (Figure 1b and 1c). Since the distributions of these values did not follow a normal distribution (according to Kolmogorov-Smirnov test with a p-value threshold of 0.05), we used a distribution-independent procedure to evaluate the probability of each point of these distributions. performing a bootstrap sampling (Figure 1d and 1e) of all the obtained values. In other words, a probability was assigned to each of the values [e.g. P(A)] of these two distributions computing it as P(A) = n(A)/N, where n(A) is the number of times in which the observed value (of ΔGC and δ*, respectively) was greater than the other (128.569) values after N samplings. By doing so, two distinct p-values (Figure 1f and 1g) were associated to each sequence of the dataset: the first accounting for the probability of a gene to be atypical in terms of δ^∗ ^and the other accounting for the probability of a gene to be atypical in terms of ΔGC. Further on, these two distinct p-values were integrated in a single one according to the Fisher method [26]. In its basic form, the Fisher method is used to combine the results from two (or more) tests bearing upon the same overall null hypothesis. In other words, Fisher's method combines p-values into one test statistic (X^2^) using the formula: X^2 ^= -2∑log_e_(p_i_), where p*i *is the p-value for the *i*^th ^hypothesis test. Accordingly, (Figure 1h) only those sequences identified at a confidence interval (CI) greater than 95% were considered PAGs. Moreover, in order to explore different CI thresholds, we also collected gene sets that were identified as atypical with lower confidence values (i.e. 70%, 80% and 90%). The whole pipeline has been implemented in Perl codes and is available upon request. As it might be expected, lower CI thresholds allowed the assembly of larger PAGs dataset, ranging from 14731 (with a CI of 90%) to almost 40000 (with a CI of 70%) (*see *Additional file 1).

### Identification of PAGs source molecules

In order to identify the most likely source molecule of identified PAGS we developed a similarity-oriented computational pipeline according to which each of the identified PAG was used as a query for a BLAST [27] search against three different databases, each of which embedding 30000 sequences retrieved from NCBI plasmid, phage and chromosome databases (see Methods), respectively. For each of the BLAST searches, only the best BLAST hit was considered, in order to reduce any possible bias due to the presence of closely related sequences in the database that would falsely increase the number of homologs for a given ORF. This strategy was repeated 1000 times for each PAG and, for each of the 1000 runs, new plasmid, chromosome and phage databases were assembled, randomly sampling 30000 sequences from the NCBI databases. Finally, the putative source molecule was identified according to the database (plasmid, phage or chromosome) that produced the highest number of best hits after 1000 BLAST probings.

### Statistics

All statistical tests were performed with the R package [28]. All other statistical analyses were performed using *in-house *developed Perl scripts.

## Results and Discussion

### PAGs general feaures

#### PAGs distribution

**Figure 2:**
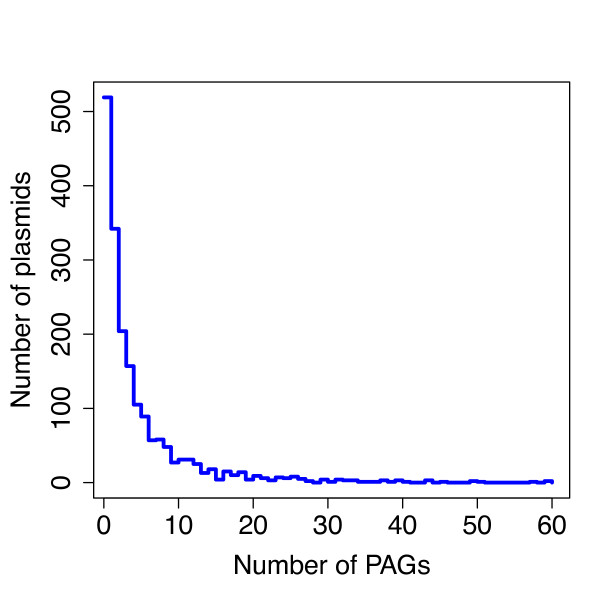
**PAGs distribution**. PAGs ditribution across the plasmids composing the dataset.

We applied the computational pipeline described in Methods (Figure 1) to 1853 archaeal (51) and bacterial (1802) plasmids (128.569 ORFs in total) in order to explore their mosaic structure, using a confidence value of 95% (see Methods for details). In this way we were able to collect 8065 compositionally atypical ORFs, denominated PAGs (that is 6.2% of all the 128.569 encoded proteins, Table 1) distributed through 1354 plasmid molecules (73% of the entire dataset). The remaining plasmids (519, 27% of the dataset) do not possess any atypical gene at all. The fasta format of PAGs sequences (including those retrieved at a CI of 70%, 80% and 90%) is available as Additional file 2. The analysis of the distribution of PAGs (that is the number of PAGs *per *plasmid) within the assembled plasmid dataset revealed that they are not evenly distributed (Figure 2), ranging from 59 (found in *Methylobacterium extorquens *AM1) to 1 PAG (342 plasmids). Overall the distribution of PAGs across the microbial dataset showed that a high number of plasmids possess a small number of PAGs (or do not possess any PAGs at all) whereas only a few of them possess higher number of atypical ORFs (Figure 2). Importantly, the same trend was observed with PAGs dataset retrieved at lower CI, i.e. 70%, 80% and 90% (Additional file 3).

**Table 1:** PAGs general features

N. of analyzed plasmids	1853
N. of analyzed sequences	128.569

N. of retrieved PAGs	8065

Percentage of PAGs	6.2%

Average PAGs for plasmid	4.3

PAGs in clusters (≥ 2 genes)	1653

Cluster of PAGs (≥ 2 genes)	677

PAGs *per *clusters (on average)	2.4

**Figure 3:**
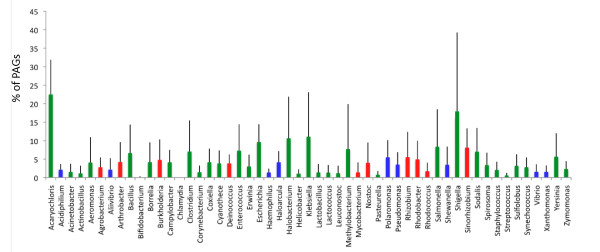
**Taxonomical distribution of PAGs**. Relative PAGs abundance and standard deviations across the most represented genera of the dataset. Red and green bars represent statistically PAGs depleted and enriched genera, respectively. Blue bars represent genera for which the content of PAGs was not statistically different from that expected by chance.

To find possible correlations between PAGs distribution and the taxonomy of their hosting cells, we evaluated the percentage of PAGs for each of the genera embedded in our dataset. For clarity purposes, in Figure 3 we report only results concerning those genera for which it was possible to retrieve at least 7 plasmids during dataset assembly procedures (*see *Methods); complete results are provided in Additional file 4. Overall, we found that PAGs number varies greatly among the different genera (Figure 3). Besides, since the taxonomical distribution of PAGs might be strongly influenced by the overall number of sampled sequences from each genus, we evaluated the statistical significance of this distribution by comparing them with 10000 randomly assembled ones, obtained re-shuffling the 8065 PAGs within the entire plasmids dataset and counting the fraction of times each genus possessed a number of PAGs greater or lower than the observed one. This gives a *p*-value accounting for the statistical significance of the number of PAGs retrieved within each genus in respect to our data model, i.e. that PAGs were randomly distributed within the plasmids.

Overall we found that 71 (out of 106) genera possessed an amount of PAGs that was higher (55 PAGs enriched genera) or lower (16 PAGs depleted genera) than that expected to occur by chance (p-value < 10^-4^).

The two best-scoring genera in terms of PAGs content were *Acaryochloris *and *Shigella *(22.5% and 17.9% of all the plasmids encoded proteins, respectively); in both cases PAGs enrichment was shown to be statistically significant. Interestingly, plasmids from *A. marina *were already shown to possess metabolic capabilitities that were probably acquired HGT transfer [29], thus confirming their mosaic structure assessed by PAGs analysis. Similarly, the mosaicism of *Shigella *plasmids is a well known issue [30-32] and has been demonstrated to be biologically relevant since it has probably allowed these strains to acquire pathogenic adaptation [31,33].

Both genera resulted PAGs enriched also when lower CI thresholds were applied (respectively 48.5, 74.3 and 103.1 PAGs/plasmid at 90%, 80% and 70% threshold for *Acaryochloris *and 31.1, 52.4 and 66.8 in the case of *Shigella *representatives) although lowering CI values below 80% resulted in statistical inconsistency, likely due to the inclusion of too many false positives in the dataset.

At all CI thresholds analyzed, also PAGs depleted plasmids span over a large taxonomic range, comprising Actinobacteria, Firmicutes, Proteobacteria and Cyanobacteria. Interestingly, we found that α-proteobacterial plasmids (mainly from *Sinorhizobium*, *Agrobacterium *and *Rhizobium*) are higlhy represented within PAGs-depleted plasmids, suggesting that plasmids hosted by representatives of this taxonomic unit might undergo recombination/HGT events less frequently than the others. The finding that these bacteria harbor a lower number of PAGs than that expected by chance, might be accounted for by the fact that these are mainly soil inhabiting microorganisms. Indeed, it has been proposed that bacteria inhabiting this ecological niche might represent a less connected component of the overall plasmids-mediated HGT network [34]. Accordingly, this might partially explain their lower number of PAGs. Alternatively, since it has been suggested that there is considerable gene flow between replicons in the rhizobiaceae [35,36], it can be surmised that these bacteria frequently undergo recombination with the chromosomes of their hosting cells. However it is noteworthy that, in most cases, compositional features of rhizobiaceae replicons are pretty similar (as, for example, the GC% content (around 60%) in *Sinorhizobium *and *Rhizobium *representatives along all the replicons inhabiting the same cell). Thus, it is absolutely possbile that a fraction of this internal recombination event(s) may remain obscure due to the composition-oriented pipeline developed in this work. Interestingly, for what concerns identified PAGs (for whose identification another compositional measure was addedd to GC% content, i.e. δ*) we found that in alpha-proteobacteria chromosomal origin PAGs are more represented in respect to the whole dataset (10% and 5%, respectively, see below), suggesting that plasmids from these microorganisms might really have more genes of chromosomal origin that what is seen in other species.

#### PAGs and plasmids size

**Figure 4:**
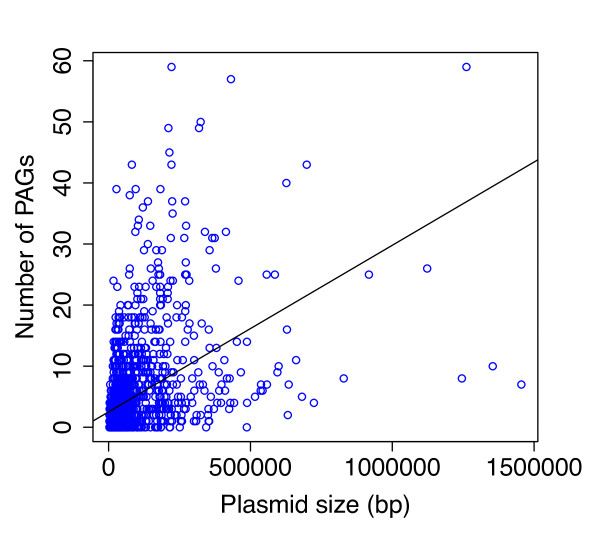
**PAGs and plamids size**. Scatterplot illustrating the correlation existing between plasmids size and their PAGs content.

In principle, the acquisition and the maintainance of exogeneous DNA by a plasmid molecule might lead to the expansion of its coding capabilities and, parallely, to an increase of its size. Moreover, it might be expected that larger plasmids possess a higher number of "entry points" for exogeneous DNA in respect to smaller ones, thus somehow promoting the acquisition of novel genetic material. To test these hypotheses, we compared the number of PAGs possessed by each plasmid with its size (Figure 4). We found only a slightly positive correlation (R^2 ^= 0.21) between the length (in bp) of each plasmid and its PAGs content. Indeed, within our dataset we retrieved plasmids completely riddled in PAGs as, for example, Far04_lp28-1 plasmid from *Borrelia garinii *Far04 (27689 nt) where we found almost 80% of atypical genes. Conversely, in a plasmid of similar size (e.g. pXAG81 from *Xanthomonas axonopodis pv. Glycines*, 26721 bp) we were not able to detect any PAG. This finding might suggest that plasmids can undergo recombination with other informative molecules and/or HGT regardless of their size. Moreover, this might also indicate that, the acquisition of foreign DNA might not be the only force driving the growth and the expansion of plasmids coding capabilities and that other molecular mechanisms (e.g. gene duplication) might play a certain role. Similar trends (with R^2 ^values raging from 0.24 to 0.48 were observed) also when PAGs dataset retrieved with lower CI thresholds were analyzed (Additional file 5).

#### PAGs functions

**Figure 5:**
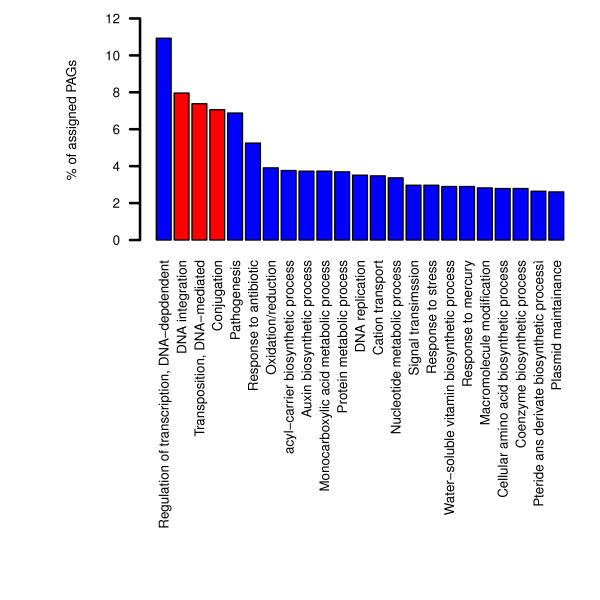
**PAGs functions**. Histogram showing the distribution of the main biological processes in which PAGs are involved. Red bars refer to processes generally related to DNA mobilization.

We aimed at identifying the most represented functions performed by PAGs. To fulfill this task we adopted the computational pipeline implemented in Blast2GO (B2GO) [37] and performed an automated functional annotation. For most of PAGs it was not possible to identify any associated function. This was somehow expected, since it has already been shown [3] that the function of most of the plasmid encoded proteins is still unknown. Specifically, on a total of 8065 sequences used as input for B2GO, 803 PAGs did not retrieve any BLAST hit and 4795 retrieved an hypothetical ortholog in the database (in almost all cases corresponding to the query itself) but did not produce any mapping to a known biological function. Hence, overall, an important fraction of PAGs (5598 sequences, almost 68% of the total) could not be linked to any hit in functional databases and, as a consequence, to any known biological function. For the remaining 2467, it was possible to retrieve a putative biological process. For clarity purposes, we show only those biological process that possessed at least 70 representatives within the PAGs dataset (Figure 5, complete results are shown as Additional file 6).

As shown in Figure 5, most of the PAGs encoded proteins (around 22% of all the annotated ones) are associated to those molecular functions that are able to catalyze the movement of DNA among and within informative molecules, that is DNA integration, transposition and conjugation (7.9%, 7.3% and 7%, respectively, see red bars in Figure 5). Notably, this result partially validates the applied approach for PAGs detection, since genes that are able to move across different molecules are also expected to differ from a compositional viewpoint from the correspondig hosting molecule. Moreover, we found that another important fraction (303 sequences, corresponding to 10.9% of all the PAGs for which a putative function was retrieved) is represented by proteins involved in transcription regulation (DNA-mediated). A further investigation revealed that these proteins are mainly involved in important biological processes that are usually associated to plasmids and\or trasposons and that have a (more or less) long documentated history of HGTs, such as mercury detoxification (e.g. MerD transcriptional regulator from plasmid pEC-IMP, GI: 226807665), tetracycline resistance (e.g. TetR trascriptional regulator of *Salmonella typhimurium *R64 plasmid, GI: 32470145) and virulence (e.g. VirF trascriptional regulator in plasmid pSS_046 of *Shigella sonnei *Ss046, GI: 74314878). The presence of such proteins within the assembled PAGs dataset is intriguing. Indeed, it might be expected that the introgression of proteins capable of interfering with the overall (complex) regulatory network of the cell might be (quite) "dangerous" (from a biological viewpoint) and prone to be counterselected by the novel hosting cell. However, further analyses (see below) revealed that, a considerable amount of PAGs are embedded in more or less compact clusters, involved in processes that are known to be often spread by HGT (including virulence, antibiotic resistance and heavy metals detoxification). Accordingly, atypical trascriptional regulators might be part of this gene clusters and, consequently, might be involved in the regulation of the flanking regions. Alternatively, it can be surmised that, after the introgression of the atypical transcriptional regulator, some modifications (i.e. mutations) might have occurred, rendering the newly acquired sequence compatible with the overall "new" regulatory network, that is more easily recognizable by the transcriptional apparatus of the host cell, as experimentally demonstrated [38]. After these two biological processes, we found that pathogenesis- and antibiotic resistance-related sequences are the most abundant among annotated PAGs (6.8% and 5.2%, respectively). Remarkably, the finding that these two biological processes are highly represented among atypical genes underlines the key role that HGT possesses in the spreading of these two important biological features within the microbial world.

**Figure 6:**
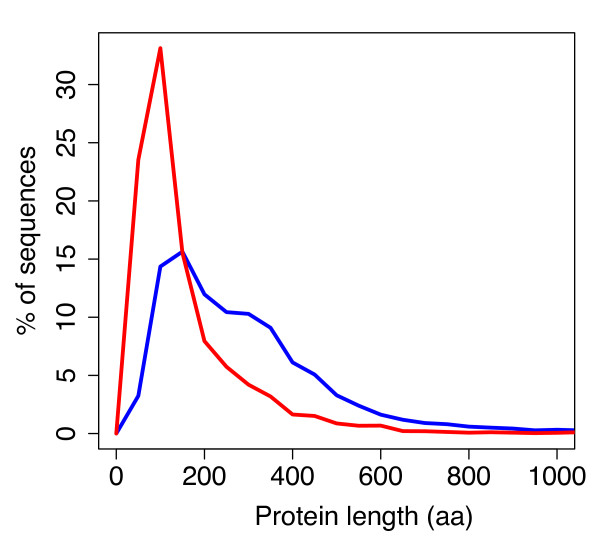
**PAGs length**. The length of PAGs encoded proteins (red line) compared to the length of all the other plasmids encoded proteins (blue line) present in the dataset.

Finally, the high percentage of PAGs that did not retrieve any BLAST hit during the B2GO searches, might speak towards the presence of an important fraction of pseudogenes within our PAGs dataset. Accordingly, these genes might represent aberrant sequences that, in the absence of strong selective pressure, have accumulated a great number of mutations and have evolved beyond recognition. The fact that we identified them as PAGs might rely on the observation that pseudogenes are often originated from failed HGT events between two different source molecules [39]. Interestingly, also premature stop codons (and, consequently, shorter proteins) have been indicated as a typical characteristic of pseudogenes that, in turn, are known to originate (at least in part) from failed HGT [39]. Indeed, PAGs encoded proteins are, on average, shorter (136,5 aminoacids) than not-atypical ones (274,2 aminoacids, Figure 6), suggesting that part of the PAGs retrieved might indeed be pseudogenes, probably originated from unsuccessful integration in the host plasmids. Notably, PAGs datasests retrieved at different thresholds followed the same overall trend (Additional File 7).

### PAGs source molecules

As previously pointed out, PAGs may derive from homologous recombination or homology-facilitated illegitimate recombination between a given plasmid and/or other informative molecules, that is phages, chromosomes or other plasmids. Hence, we aimed at identifying the putative source molecule of PAGs. To this purpose, we have developed a computational approach (see Methods) that, on the basis of the number of orthologs present in different (and randomly assembled) plasmid, chromosome and phage databases allows to assign the most likely source molecule to a set of sequences (in our case the 8065 PAGs). It must be stated clear that this kind of strategy has only an explorative purpose and might be strongly influenced by the present content of public databases that, undoubtedly, represents just a glimpse of the real biodiversity present in nature. For this reason, the reliability of the developed approach was firstly revealed by a test on a set of 1000 likely chromosomal native sequences retrieved by Cortez et al. [10] from a set of 119 bacterial and archaeal chromosomes. Indeed, results of this preliminar screening showed that almost 90% of the probed sequences were correctly annotated, i.e. resulted to possess a putative chromosome origin. Furthermore, we sought to test the implemented strategy on a set of sequences of plasmid and phage origin. However, as already pointed out, in these cases a set of "core" sequences is very difficult (if not impossible) to be retrieved. Hence, the previously described pipeline was applied on two distinct randomly assembled datasets of viral and plasmids sequences, embedding 1000 sequences each. Results similar to those obtained with (likely) chromosome native sequences were obtained (84% and 82% of "correct" identifications in the case of plasmdis and phages, respectively) thus suggesting that, in most cases, the implemented strategy is able to detect the correct source molecule of a given sequence.

**Figure 7:**
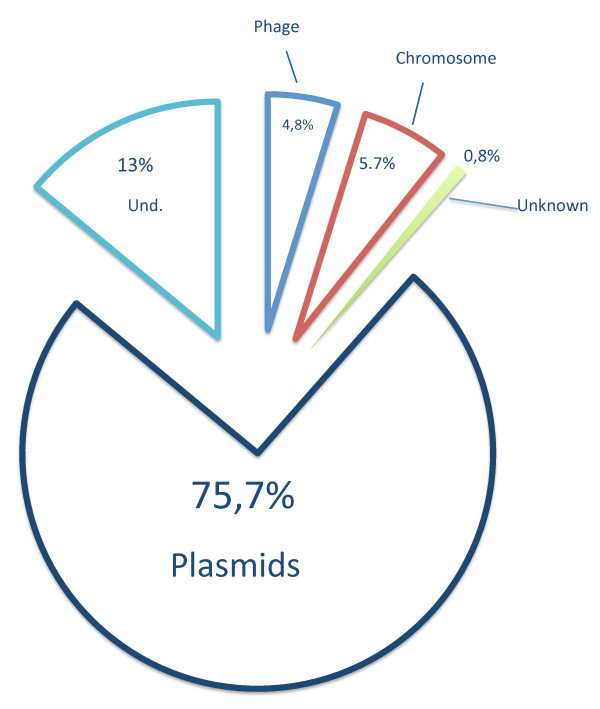
**PAGs origin**. Pie chart showing the most likely source molecule of retrieved PAGs. Und. stands for "Undetermined " (*see *text for details).

Applying the described strategy to our PAGs dataset revealed that, as shown in Figure 7, most of the identified PAGs (almost 75%) are of likely plasmid origin. On the contrary, chromosome and phage sequences appear to be much rarer, being represented by 5.7% and 4.8% of PAGs, respectively. Remarkably, almost 13% of all the probed PAGs (1087), did not retrieve a clear corresponding match in the plasmid, chromosome and phage databases and, for this reason, their origin remained "undetermined" (Figure 7). Finally, less than 1% of the PAGs did not retrieve any match in any of the databases during our BLAST probings and were labelled as "Not Found" in Figure 7. Again, also sequences retrieved with lower CI thresholds showed the same hypothetical origin, with likely plasmid orgin sequences being largely over-represented (73.8%, 72.9% and 73.5% for 70%, 80% and 90% CI thresholds, respectively). Taken together these results indicate that most of the PAGs are likely to appear only in plasmids rather than begin shared among different types of informative molecules. We speculate here that most of retrieved PAGs likely derive from plasmid-plasmid gene exchanges and that, *vice versa*, integrations following virus-plasmid and/or chromosome-plasmid gene exchange appear to be less frequent. Remarkably, this finding fits with previous analyses [40] on the structure of the overall DNA-exchange network and that suggested that DNA families are mostly exchanged among the same type of DNA carriers (i.e. plasmids, phages or chromosomes). Our analyses indicate that this might hold true (at least) in the case of plasmid molecules.

### Clusters of PAGs

**Figure 8:**
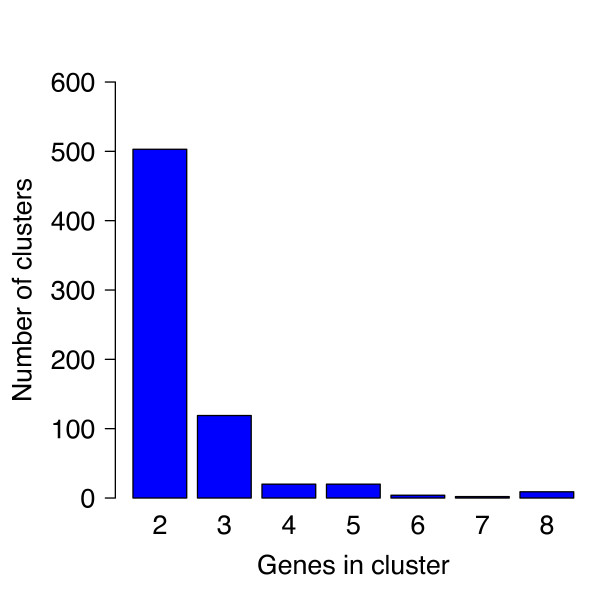
**PAGs clusters**. PAGs clusters size distribution. Clusters longer than 8 genes are not shown here.

Chromosomal atypical genes are often organized in (more or less) tight clusters. These chromosomal regions, named genomic islands [10], embeds, for example, genes involved in pilus and fimbriae formation, lipopolysaccharide biosynthesis or virulence and have been shown to have played a crucial role in microbial evolution [41]. Similarly, PAGs might either stand alone or be embedded in (more or less compact) clusters on their source molecule representing, for example, successfully integrated transposons and/or integrons. Hence, we investigated this issue and evaluated if retrieved PAGs lied in clusters or were scattered throughout the corresponding plasmid. We estimated gene clustering at three different gene distance thresholds, that is 100, 200 and 300 bp. Results shown below refer to genes that are not separated by more than 200 bp (results for 100 and 300 bp are provided as Additional file 8, together with clusters distribution retrieved at different CI thresholds). Overall we found that 1653 PAGs (on a total of 8065) are embedded in 677 clusters of different size (Table 1 and Figure 8). The complete list of all identified PAGs clusters, together with their corresponding organisms and GI codes, are provided as additional material (*see *Additional file 9). Most of them (503) are bi-cistronic clusters, while another important fraction (119) is represented by arrays of three genes. Longer clusters were quite rare and we found only 26, 21 and 5 clusters embedding 4, 5 and 6 genes, respectively. This trend is in partial agreement with previous findings on cromosomal clusters of atypical genes [10] and suggests that PAGs clusters are quickly fragmented and/or eroded following integration. Alternatively, this could suggest that the transfer and integration of shorter clusters is somehow favoured in respect to longer ones. Finally, as shown in Additional file 10, short gene arrays seem to be more frequent also when PAGs were retrieved al lower CIs.

Among identified PAGs clusters, some possess a partially documented evolutionary history, mainly driven by HGT/recombination events as, for example, mercury resistance gene cluster(s). In fact, mercury resistance genes (*mer*) have been usually found embedded in a single compact operon [42] that, in turn, has been suggested to represent an aberrant mercury resistance transposon (namely TndPKHLK2) that, in some cases, has lost those genes responsible for its transposition [43]. The analysis of PAGs clusters allowed the identification of (at least) 10 different *mer *clusters (see, Additional file 10) that showed a different composition in respect to the source molecule, thus revealing the pivotal role of HGT in the spreading of this metabolic ability across bacteria belonging to (sometimes) very different taxonomical units and inhabiting separate ecological niches. Moreover, other gene clusters (or part thereof) coding for important biological traits (i.e. antibiotic resistance, host invasion and cobalamin biosynthesis) were retrieved. For example invasion-associated genes encoding proteins involved in invasion of mammalian cells were found in atypical clusters on plasmids retrieved from different specie of *Shigella *genus (see Additional file 10) providing further support to the idea that one (or more) HGT envent(s) played a role in spreading this feature within representatives of this genus [32,44]. Similarly, plasmid mediated HGT seems to have contributed to the spreading of other key metabolic traits in microbial representatives, as, for example, cobalamin biosynthesis [45] and tetracycline resistance for which very similar (atypical) gene clusters were retrieved from very distantly related microorganisms, including *Geobacter*, *Halorubrum*, *Methylibium*, *Methylobacterium *and *Deinococcus *representatives in the case of cobalamin biosynthesis and *Escherichia*, *Klebsiella*, *Aeromonas*, *Serratia*, *Yersinia *and *Enterobacter *in the case of *tet *genes (see Additional file 10).

### PAGs and conjugative plasmids

**Figure 9:**
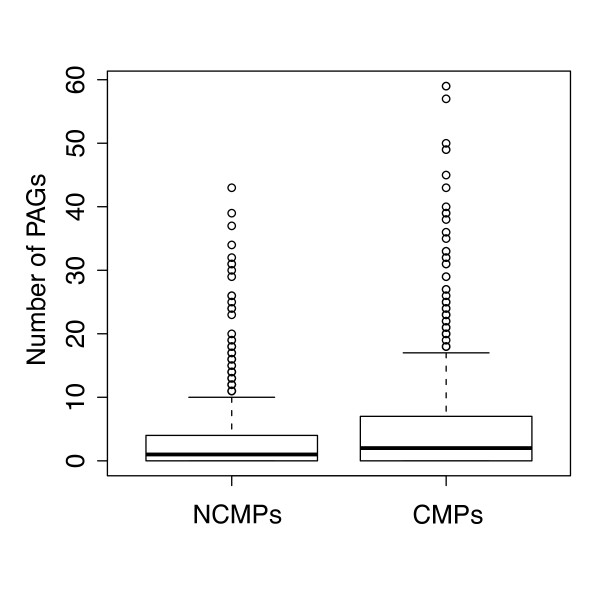
**PAGs in conjugative plasmids**. Abundance of PAGs in conjugative/mobilizable plasmids (CMPs) and not conjugative/mobilizable plasmids (NCMPs).

Among plasmids, conjugative ones have been defined "vessels" of the communal gene pool [11]. Indeed, this class of plasmids possesses the ability to "visit" different cells and, in principle, undergo genetic rearrangements (such as homologous recombination) with other plasmids and/or other informative molecules (phages and chromosomes). For this reason, conjugative plasmids might be expected to possess a higher amount of atypical genes in respect to plasmids that are not (or are less) mobilizable. To test this hypothesis, we performed a comparative analysis of the amount of PAGs belonging to conjugative and non-conjugative plasmids. The conjugative/mobilizable plasmids (hereinafter referred to as CMPs) embedded in our dataset were identified by searching for their propagation-related genes adopting the following strategy: 1) a dataset of genes involved in the propagation of conjugative plasmids (*tra *and *mob *like genes) was assembled probing a search on the ACLAME database http://aclame.ulb.ac.be/; 2) a BLAST search with each of the sequence of our dataset against the *tra*/*mob*-like database previously assembled (setting a minimum e-value of 1e-20) was carried out and then 3) the number of *tra*/*mob*-like genes was determined for each plasmid. In this way we were able to divide our plasmid dataset into (693) conjugative/mobilizable plasmids (CMPs, possessing at least one gene involved in conjugation and/or mobilization) and (1160) not conjugative/mobilizable plasmids (NCMPs, i.e. plasmids that do not possess any *tra *or *mob *related genes). Data obtained revealed that, on average, CMPs harbor a higher number of PAGs than that exhibited by NCMPs and that the distribution of PAGs in these two classes of plasmids were significantly different (Mann-Whitney test, p-value < 0.001). Indeed, CMPs possess, on average, 5.7 PAGs, whereas NCMPs posssess only 2.9 atypical ORFs for each plasmid. Data obtained are summarized in Figure 9 which shows the different trends for CMPs and NCMPs. This holds true also when PAGs dataset retrieved at lower thresholds (i.e. 70%, 80% and 90%) were analyzed (Additional File 11)

The high number of PAGs retrieved in CMPs provide strong support to the idea that plasmids have played (and are still playing) a central role in microbial evolution [11]. In fact, our data suggest that, by visiting different cells, CMPs can undergo recombination event(s) with the host's DNA molecules more frequently than NCMPs; consequently, they can probably acquire pieces of exogeneuos DNA that, in turn, can be further spread within the whole microbial communities. This idea is also partially supported by the finding that CMPs, on average, possess more and longer PAGs clusters in respect to NCMPs as reported in Additional file 12.

## Conclusions

Within the complex evolutionary network of plasmids, new functional blocks are added and exchanged. However, to date, information on plasmids atypical regions is available only for a very limited number of plasmids and/or microorganisms.

In this work we have developed a computational pipeline to detect compositionally atypical ORFs that reside on plasmids and performed a large scale analysis of them. Implementing our strategy on a dataset of nearly 2000 plasmids we have identified 8065 PAGs, almost 6% of all the analyzed ORFs. Accordingly, these PAGS are likely the outcome of (one or more) HGT event(s), although it must be mentioned the hypothesis that, at least part of them, may derive from events of internal recombination with chromosome(s) inhabiting the same cytoplasms but that, in some cases, may possess different compositional features in respect to the corresponding plasmids (as suggested in [7]).

It is worth of noticing that the total amount of retrieved PAGs is, on average, lower than that estimated for chromosomes (10-15%) [10,20]. This might be due to the high confidence interval (C.I. 95%) applied during our PAGs retrieval pipeline (Figure 1), that might have led to a partial understimatation of the actual amount of atypical ORFs that are integrated on plasmids. Indeed, applying the same pipeline for PAGs retrieval with lower CI thresholds allowed to assemble larger PAGS datasets (embedding 14731, 27201 and 38950 sequences at 70%, 80% and 90% CIs, respectively). Nevertheless analyses on these (lower confidence) assembled datasets revealed overlapping trends, suggesting that the possible exclusion of some false negatives did not influence the general conclusions that can be drawn on PAGs and, more in general, on plasmid-mediated HGT. Overall, we found that PAGs are not uniformly distributed among the sampled plasmids dataset. Indeed most of the plasmids harbor a few atypical genes or do not possess any atypical gene at all, wehreas PAGs enriched plasmids are progressively more rare. This finding is in partial agreement with previous findings on horizontal flow of plasmid genes [34] and suggests that all plasmids may not contribute equally to the overall horizontal flow of genes but, instead, some of them may occupy more central positions in the overall network of HGT events. In particular, this role might be covered by conjugative plasmids that have been shown to possess, on average, a higher amount of atypical regions in respect to not mobilizable ones.

Interestingly, plasmids size does not sensibly correlate with PAGs content. This result may provide important evolutionary insights, suggesting that the acquisition of exogeneous DNA (i.e. HGT) might not be the only force driving the plasmids assembly and the expansion of their coding capabilities. Indeed, it might be possible that other molecular mechanisms play a role in this process, such as gene duplication (possibly followed by evolutionary divergence) (Maida et al. *unpublished data*). Moreover, the fact that for a fraction of the identified PAGs it was not possible to retrieve an associated function, together with the observation that PAGs encoded proteins are, on average, shorter than not-atypical ones, points towards the presence of a fraction of pseudogenes within PAGs. Accordingly, these might have originated from unsuccessful HGT events, one of the most likely source of pseudogenes within prokaryotic genomes [39].

The automated functional annotation we have performed has revealed that, among the annotated PAGs, most are involved in the overall process of DNA mobilization, although other biologically relevant functions have been identified, such as transcription, pathogenesis and antibiotic resistance. The fact that we have retrieved the genes encoding for these functions associated to atypical DNA regions has important biological drawbacks, underlining the important role of HGT in the bacterial sharing of these key traits. Importantly PAGs are often found in multi-cistronic clusters embedding two or more genes. However, the fact that shorter plasmids (embedding 2 or 3 genes) are much more frequent than longer ones, probably indicates that PAGs clusters are fragmented following thier integration or that, alternatively, the transfer of shorter clusters is favoured in respect to longer gene arrays.

Finally, our analysis revealed that most of the PAGs might be of plasmid origin suggesting that plasmid-plasmid gene exchange might be favoured in respect to phage-plasmid and chromosome-plasmid ones. This is in partial agreement with previous findings [40] and reveals that a sort of preferential gene flow between vehicles of the same type (in our case plasmids) might exist.

## Authors' contributions

MF conceived of the study and wrote the Perl codes for the analyses. MF and EB performed the analyses. MF and RF interpreted the results and wrote the manuscript. All authors read and approved the final manuscript.

## Supplementary Material

Additional file 1 **analyzed plasmds dataset**. Table with the information concerning the 1853 analyzed plasmids, including their name, their accession number and PAGs content at different CI thresholds. Click here for file

Additional file 2 **all PAGs sequences**. Fasta files embedding all the sequences identified as PAGs at the different CI values. Click here for file

Additional file 3 **lower confidence PAGs distribution**. PAGs (retrieved at 70%, 80% and 90% CIs) ditribution across the plasmids composing the dataset. Click here for file

Additional file 4 **PAGs and taxonomy**. Full taxonomical distribution of PAGs. Click here for file

Additional file 5 **lower confidence PAGs and plasmids size for lower CI values**. Scatterplots illustrating the low positive correlation existing between plasmids size and their PAGs content, with three different PAGs datasets retrieved at a) 70%, b) 80% and c) 90%. Click here for file

Additional file 6 **lower confidence PAGs functional annotation**. a) Full COG http://www.ncbi.nih.gov/COG functional annotation of PAGs retrieved at 70%, 80% and 90% Cis. In particular, the function each of the sequences embedded in these datasets was inferred according to the one assigned to the best BLAST hit of COG database. b) Full Blast2GO functional annotation of PAGs retrieved at 95% CI. Click here for file

Additional file 7 **lower confidence PAGs length**. The length of lower confidence PAGs encoded proteins. Click here for file

Additional file 8 **clusters of lower confidence PAGs**. Results of gene clusters analysis for a) 100, 200 and 300 bp gene distance threshold and b) for PAGs retrieved at 70%, 80% and 90% CIs. Click here for file

Additional file 9 **complete PAGs clusters accession codes**. The complete list of all identified PAGs (retrieved at 95% CI threshold) clusters (at 200 bp threshold), together with their corresponding organisms and GI codes. Click here for file

Additional file 10 **the atypical *mer, tet, maxi *and *cbi *clusters**. Schematic representation of the atypical *mer, tet, maxi *and *cbi *clusters found in different plasmids of different microorganisms. Click here for file

Additional file 11 **lower confidence PAGs in CMPs and NCMPs**. Distribution of lower confidence PAGs among CMPs and NCMPs Click here for file

Additional file 12 **PAGs clusters in CMPs and NCMPs**. Distribution and length of PAGs clusters in CMPs and NCMPs. Click here for file

## References

[B1] Ochman H Lawrence JG Groisman EA Lateral gene transfer and the nature of bacterial innovation Nature 2000 405 6784 299 304 10.1038/35012500 10830951

[B2] Kohiyama M Hiraga S Matic I Radman M Bacterial sex: playing voyeurs 50 years later Science 2003 301 5634 802 803 10.1126/science.1085154 12907791

[B3] Brilli M Mengoni A Fondi M Bazzicalupo M Lio P Fani R Analysis of plasmid genes by phylogenetic profiling and visualization of homology relationships using Blast2Network BMC Bioinformatics 2008 9 551 10.1186/1471-2105-9-551 19099604 PMC2640388

[B4] Heuer H Smalla K Horizontal gene transfer between bacteria Environ Biosafety Res 2007 6 1-2 3 13 10.1051/ebr:2007034 17961477

[B5] Homma K Fukuchi S Nakamura Y Gojobori T Nishikawa K Gene cluster analysis method identifies horizontally transferred genes with high reliability and indicates that they provide the main mechanism of operon gain in 8 species of gamma-Proteobacteria Mol Biol Evol 2007 24 3 805 813 17185745 10.1093/molbev/msl206

[B6] Karlin S Burge C Dinucleotide relative abundance extremes: a genomic signature Trends Genet 1995 11 7 283 290 10.1016/S0168-9525(00)89076-9 7482779

[B7] van Passel MW Bart A Luyf AC van Kampen AH van der Ende A Compositional discordance between prokaryotic plasmids and host chromosomes BMC Genomics 2006 7 26 10.1186/1471-2164-7-26 16480495 PMC1382213

[B8] Berg OG Kurland CG Evolution of microbial genomes: sequence acquisition and loss Mol Biol Evol 2002 19 12 2265 2276 12446817 10.1093/oxfordjournals.molbev.a004050

[B9] Norman A Hansen LH She Q Sorensen SJ Nucleotide sequence of pOLA52: a conjugative IncX1 plasmid from Escherichia coli which enables biofilm formation and multidrug efflux Plasmid 2008 60 1 59 74 10.1016/j.plasmid.2008.03.003 18440636

[B10] Cortez D Forterre P Gribaldo S A hidden reservoir of integrative elements is the major source of recently acquired foreign genes and ORFans in archaeal and bacterial genomes Genome Biol 2009 10 6 R65 10.1186/gb-2009-10-6-r65 19531232 PMC2718499

[B11] Norman A Hansen LH Sorensen SJ Conjugative plasmids: vessels of the communal gene pool Philos Trans R Soc Lond B Biol Sci 2009 364 1527 2275 2289 10.1098/rstb.2009.0037 19571247 PMC2873005

[B12] Osborn AM Boltner D When phage, plasmids, and transposons collide: genomic islands, and conjugative- and mobilizable-transposons as a mosaic continuum Plasmid 2002 48 3 202 212 10.1016/S0147-619X(02)00117-8 12460536

[B13] Toussaint A Merlin C Mobile elements as a combination of functional modules Plasmid 2002 47 1 26 35 10.1006/plas.2001.1552 11798283

[B14] Boyd EF Hill CW Rich SM Hartl DL Mosaic structure of plasmids from natural populations of Escherichia coli Genetics 1996 143 3 1091 1100 8807284 10.1093/genetics/143.3.1091 PMC1207381

[B15] Osborn AM da Silva Tatley FM Steyn LM Pickup RW Saunders JR Mosaic plasmids and mosaic replicons: evolutionary lessons from the analysis of genetic diversity in IncFII-related replicons Microbiology 2000 146 Pt 9 2267 2275 10974114 10.1099/00221287-146-9-2267

[B16] Baran RH Ko H Detecting horizontally transferred and essential genes based on dinucleotide relative abundance DNA Res 2008 15 5 267 276 10.1093/dnares/dsn021 18799480 PMC2575891

[B17] Kang M Zhou R Liu L Langford PR Chen H Analysis of an Actinobacillus pleuropneumoniae multi-resistance plasmid, pHB0503 Plasmid 2009 61 2 135 139 10.1016/j.plasmid.2008.11.001 19041669

[B18] Hsiao WW Ung K Aeschliman D Bryan J Finlay BB Brinkman FS Evidence of a large novel gene pool associated with prokaryotic genomic islands PLoS Genet 2005 1 5 e62 10.1371/journal.pgen.0010062 16299586 PMC1285063

[B19] Cortez DQ Lazcano A Becerra A Comparative analysis of methodologies for the detection of horizontally transferred genes: a reassessment of first-order Markov models In Silico Biol 2005 5 5-6 581 592 16610135

[B20] Nakamura Y Itoh T Matsuda H Gojobori T Biased biological functions of horizontally transferred genes in prokaryotic genomes Nat Genet 2004 36 7 760 766 10.1038/ng1381 15208628

[B21] Garcia-Vallve S Romeu A Palau J Horizontal gene transfer in bacterial and archaeal complete genomes Genome Res 2000 10 11 1719 1725 10.1101/gr.130000 11076857 PMC310969

[B22] Ou HY Chen LL Lonnen J Chaudhuri RR Thani AB Smith R Garton NJ Hinton J Pallen M Barer MR A novel strategy for the identification of genomic islands by comparative analysis of the contents and contexts of tRNA sites in closely related bacteria Nucleic Acids Res 2006 34 1 e3 10.1093/nar/gnj005 16414954 PMC1326021

[B23] Lawrence JG Ochman H Amelioration of bacterial genomes: rates of change and exchange J Mol Evol 1997 44 4 383 397 10.1007/PL00006158 9089078

[B24] Hayes WS Borodovsky M How to interpret an anonymous bacterial genome: machine learning approach to gene identification Genome Res 1998 8 11 1154 1171 9847079 10.1101/gr.8.11.1154

[B25] van Passel MW Bart A Thygesen HH Luyf AC van Kampen AH van der Ende A An acquisition account of genomic islands based on genome signature comparisons BMC Genomics 2005 6 163 10.1186/1471-2164-6-163 16297239 PMC1310630

[B26] Fisher RA Statistical Methods for Research Workers 1925 Edimburg: Oliver and Boyd

[B27] Altschul SF Madden TL Schaffer AA Zhang J Zhang Z Miller W Lipman DJ Gapped BLAST and PSI-BLAST: a new generation of protein database search programs Nucleic Acids Res 1997 25 17 3389 3402 10.1093/nar/25.17.3389 9254694 PMC146917

[B28] R-Development-Core-Team R: A Language and Environment for Statistical Computing Vienna, Austria 2011

[B29] Swingley WD Chen M Cheung PC Conrad AL Dejesa LC Hao J Honchak BM Karbach LE Kurdoglu A Lahiri S Niche adaptation and genome expansion in the chlorophyll d-producing cyanobacterium Acaryochloris marina Proc Natl Acad Sci USA 2008 105 6 2005 2010 10.1073/pnas.0709772105 18252824 PMC2538872

[B30] Buchrieser C Glaser P Rusniok C Nedjari H D'Hauteville H Kunst F Sansonetti P Parsot C The virulence plasmid pWR100 and the repertoire of proteins secreted by the type III secretion apparatus of Shigella flexneri Mol Microbiol 2000 38 4 760 771 10.1046/j.1365-2958.2000.02179.x 11115111

[B31] Jiang Y Yang F Zhang X Yang J Chen L Yan Y Nie H Xiong Z Wang J Dong J The complete sequence and analysis of the large virulence plasmid pSS of Shigella sonnei Plasmid 2005 54 2 149 159 10.1016/j.plasmid.2005.03.002 16122562

[B32] Venkatesan MM Goldberg MB Rose DJ Grotbeck EJ Burland V Blattner FR Complete DNA sequence and analysis of the large virulence plasmid of Shigella flexneri Infect Immun 2001 69 5 3271 3285 10.1128/IAI.69.5.3271-3285.2001 11292750 PMC98286

[B33] Watanabe H Nakamura A Large plasmids associated with virulence in Shigella species have a common function necessary for epithelial cell penetration Infect Immun 1985 48 1 260 262 3980088 10.1128/iai.48.1.260-262.1985 PMC261946

[B34] Fondi M Fani R The horizontal flow of the plasmid resistome: clues from inter-generic similarity networks Environ Microbiol 2010 10.1111/j.1462-2920.2010.02295.x 20636373

[B35] Tian CF Young JP Wang ET Tamimi SM Chen WX Population mixing of Rhizobium leguminosarum bv. viciae nodulating Vicia faba: the role of recombination and lateral gene transfer FEMS Microbiol Ecol 2010 73 3 563 576 20533948 10.1111/j.1574-6941.2010.00909.x

[B36] Harrison PW Lower RP Kim NK Young JP Introducing the bacterial 'chromid': not a chromosome, not a plasmid Trends Microbiol 2010 18 4 141 148 10.1016/j.tim.2009.12.010 20080407

[B37] Conesa A Gotz S Garcia-Gomez JM Terol J Talon M Robles M Blast2GO: a universal tool for annotation, visualization and analysis in functional genomics research Bioinformatics 2005 21 18 3674 3676 10.1093/bioinformatics/bti610 16081474

[B38] Dabizzi S Ammannato S Fani R Expression of horizontally transferred gene clusters: activation by promoter-generating mutations Res Microbiol 2001 152 6 539 549 10.1016/S0923-2508(01)01228-1 11501672

[B39] Liu Y Harrison PM Kunin V Gerstein M Comprehensive analysis of pseudogenes in prokaryotes: widespread gene decay and failure of putative horizontally transferred genes Genome Biol 2004 5 9 R64 10.1186/gb-2004-5-9-r64 15345048 PMC522871

[B40] Halary S Leigh JW Cheaib B Lopez P Bapteste E Network analyses structure genetic diversity in independent genetic worlds Proc Natl Acad Sci USA 2010 107 1 127 132 10.1073/pnas.0908978107 20007769 PMC2806761

[B41] Karlin S Detecting anomalous gene clusters and pathogenicity islands in diverse bacterial genomes Trends Microbiol 2001 9 7 335 343 10.1016/S0966-842X(01)02079-0 11435108

[B42] Fondi M Bacci G Brilli M Papaleo CM Mengoni A Vaneechoutte M Dijkshoorn L Fani R Exploring the evolutionary dynamics of plasmids: the Acinetobacter pan-plasmidome BMC Evol Biol 2010 10 1 59 10.1186/1471-2148-10-59 20181243 PMC2848654

[B43] Kholodii G Mindlin S Gorlenko Z Petrova M Hobman J Nikiforov V Translocation of transposition-deficient (TndPKLH2-like) transposons in the natural environment: mechanistic insights from the study of adjacent DNA sequences Microbiology 2004 150 Pt 4 979 992 15073307 10.1099/mic.0.26844-0

[B44] Jackson RW Athanassopoulos E Tsiamis G Mansfield JW Sesma A Arnold DL Gibbon MJ Murillo J Taylor JD Vivian A Identification of a pathogenicity island, which contains genes for virulence and avirulence, on a large native plasmid in the bean pathogen Pseudomonas syringae pathovar phaseolicola Proc Natl Acad Sci USA 1999 96 19 10875 10880 10.1073/pnas.96.19.10875 10485919 PMC17976

[B45] Morita H Toh H Fukuda S Horikawa H Oshima K Suzuki T Murakami M Hisamatsu S Kato Y Takizawa T Comparative genome analysis of Lactobacillus reuteri and Lactobacillus fermentum reveal a genomic island for reuterin and cobalamin production DNA Res 2008 15 3 151 161 10.1093/dnares/dsn009 18487258 PMC2650639

