# Supplementary figures and images for: The mosaicism of plasmids revealed by atypical genes detection and analysis
Source: BMC Genomics. 2011 Aug 8;12:403. doi: 10.1186/1471-2164-12-403 (PMC3166947; doi:10.1186/1471-2164-12-403)

## Slide 1
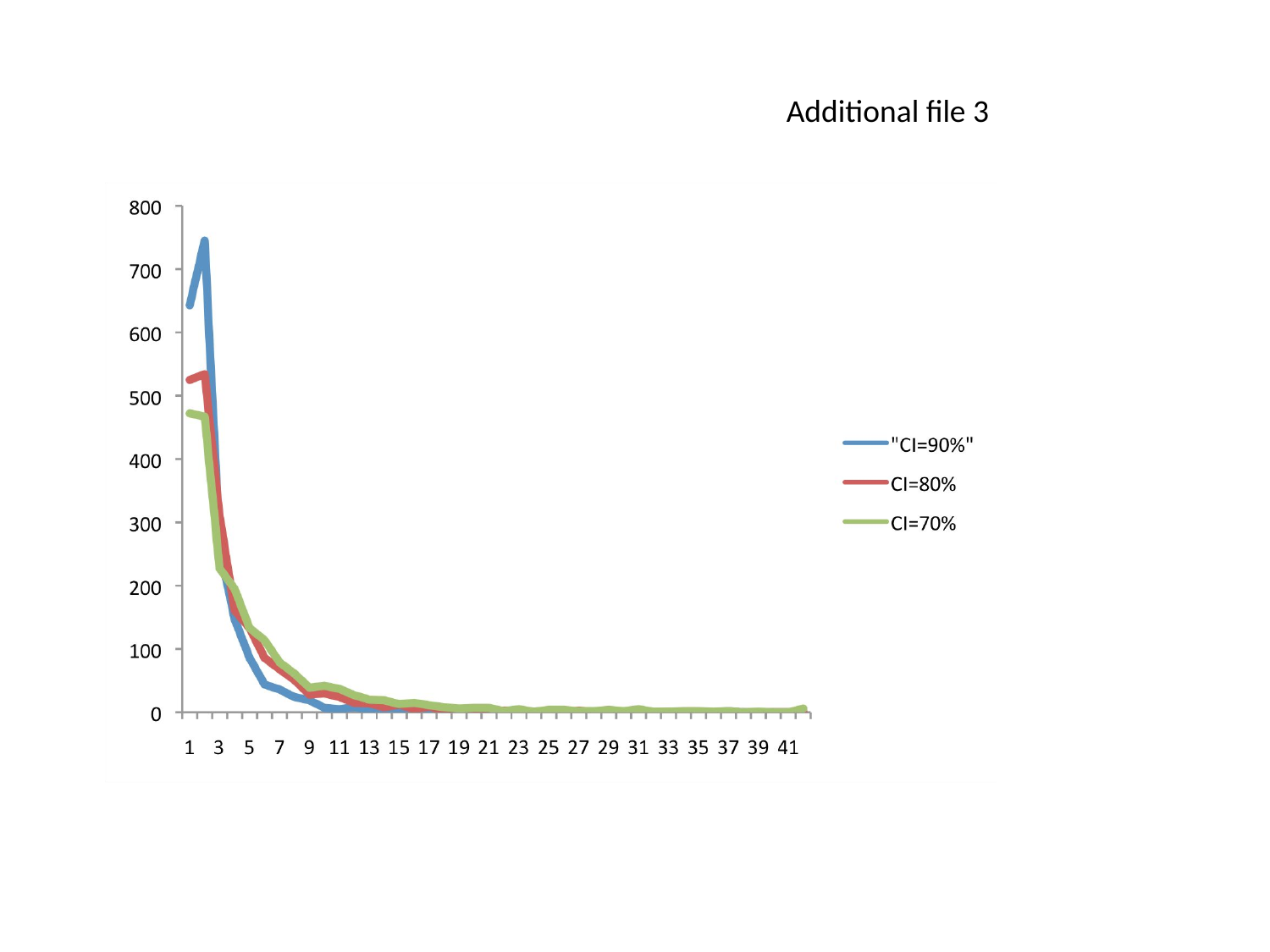

Additional file 3

Supplement: Additional file 3 — lower confidence PAGs distribution. PAGs (retrieved at 70%, 80% and 90% CIs) ditribution across the plasmids composing the dataset. [file 1471-2164-12-403-S3.PPT]

## Slide 1
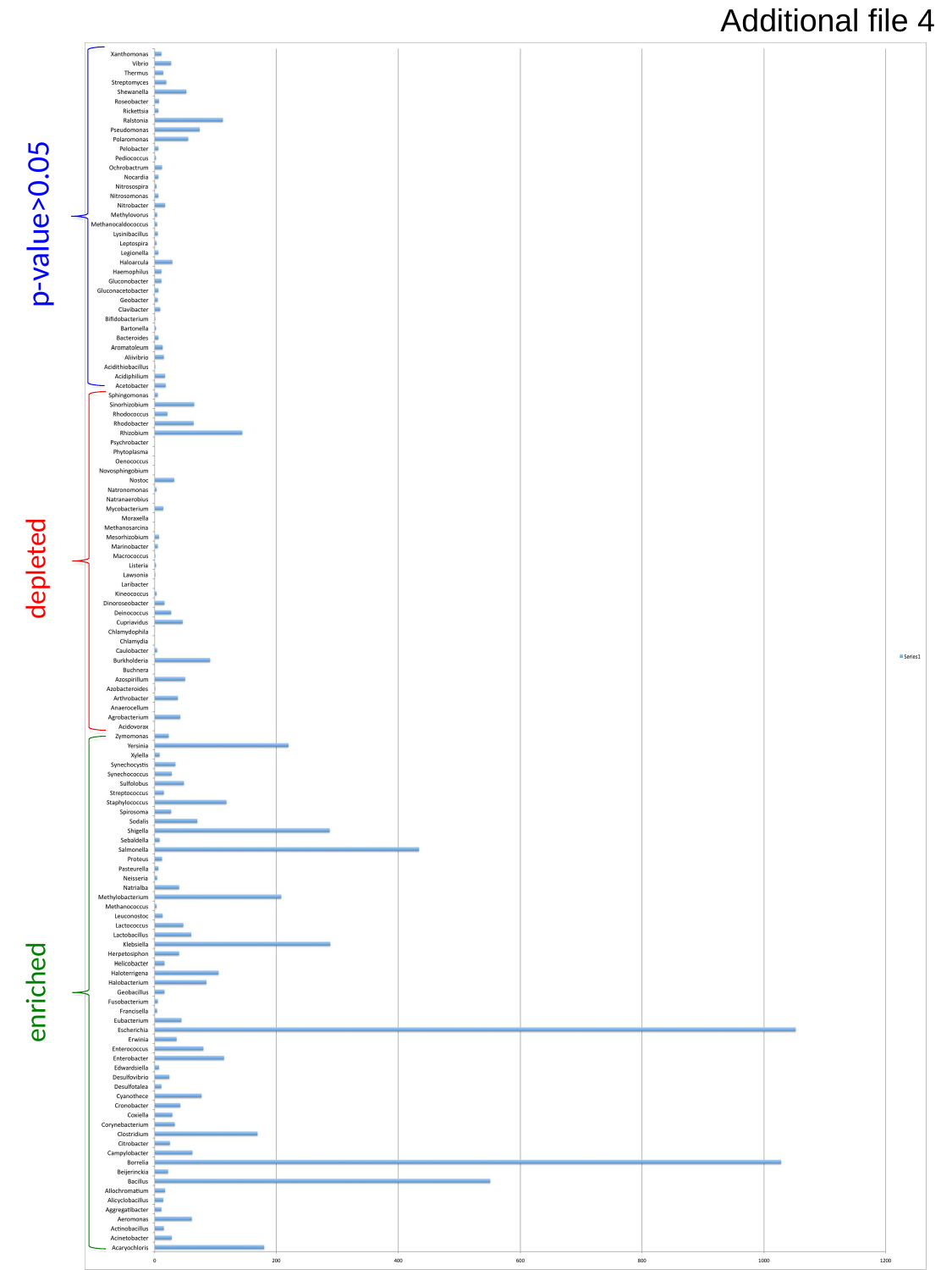

Additional file 4
p-value>0.05
depleted
enriched

Supplement: Additional file 4 — PAGs and taxonomy. Full taxonomical distribution of PAGs. [file 1471-2164-12-403-S4.PPT]

## Slide 1
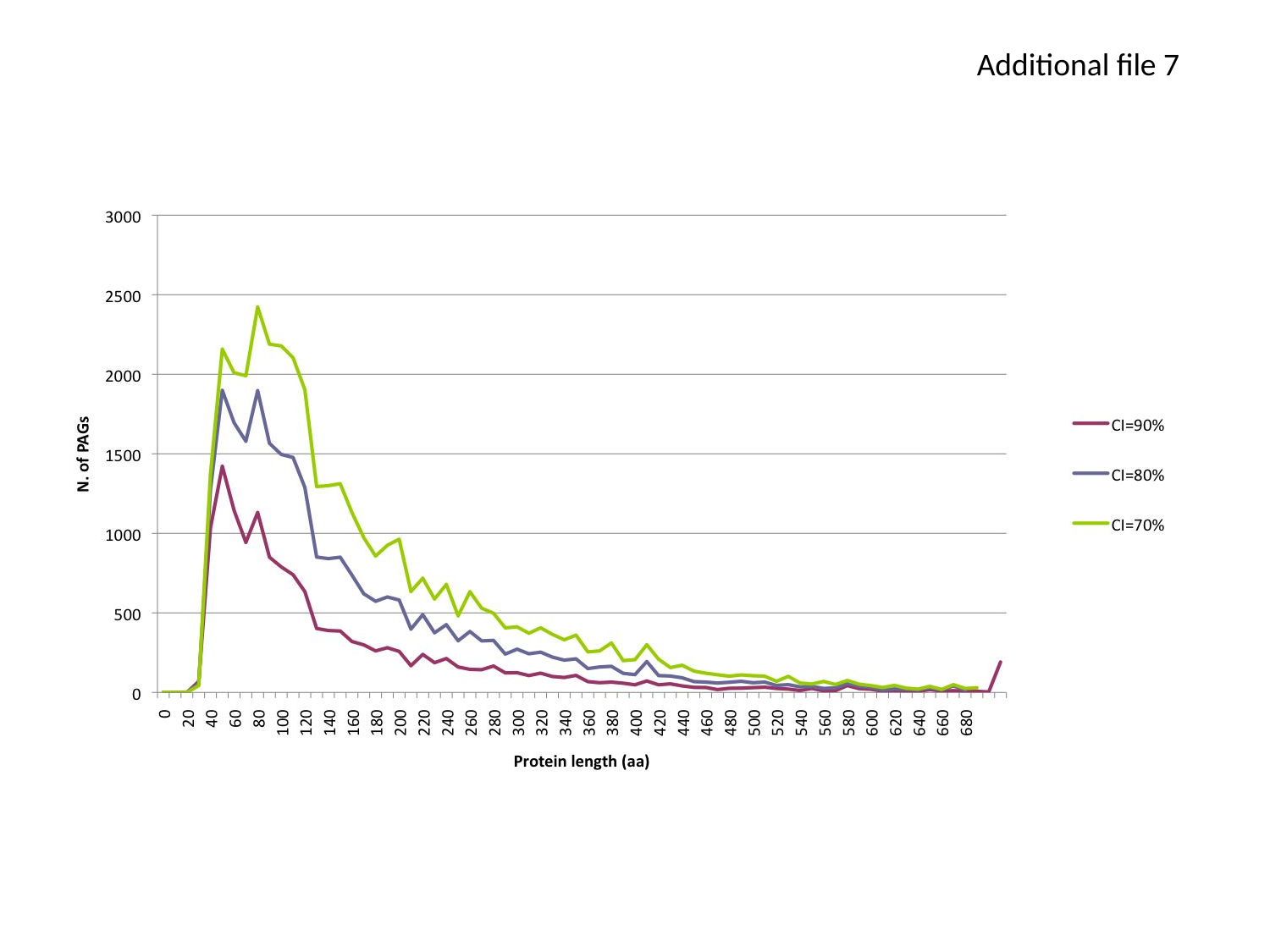

Additional file 7

Supplement: Additional file 7 — lower confidence PAGs length. The length of lower confidence PAGs encoded proteins. [file 1471-2164-12-403-S7.PPT]

## Slide 1
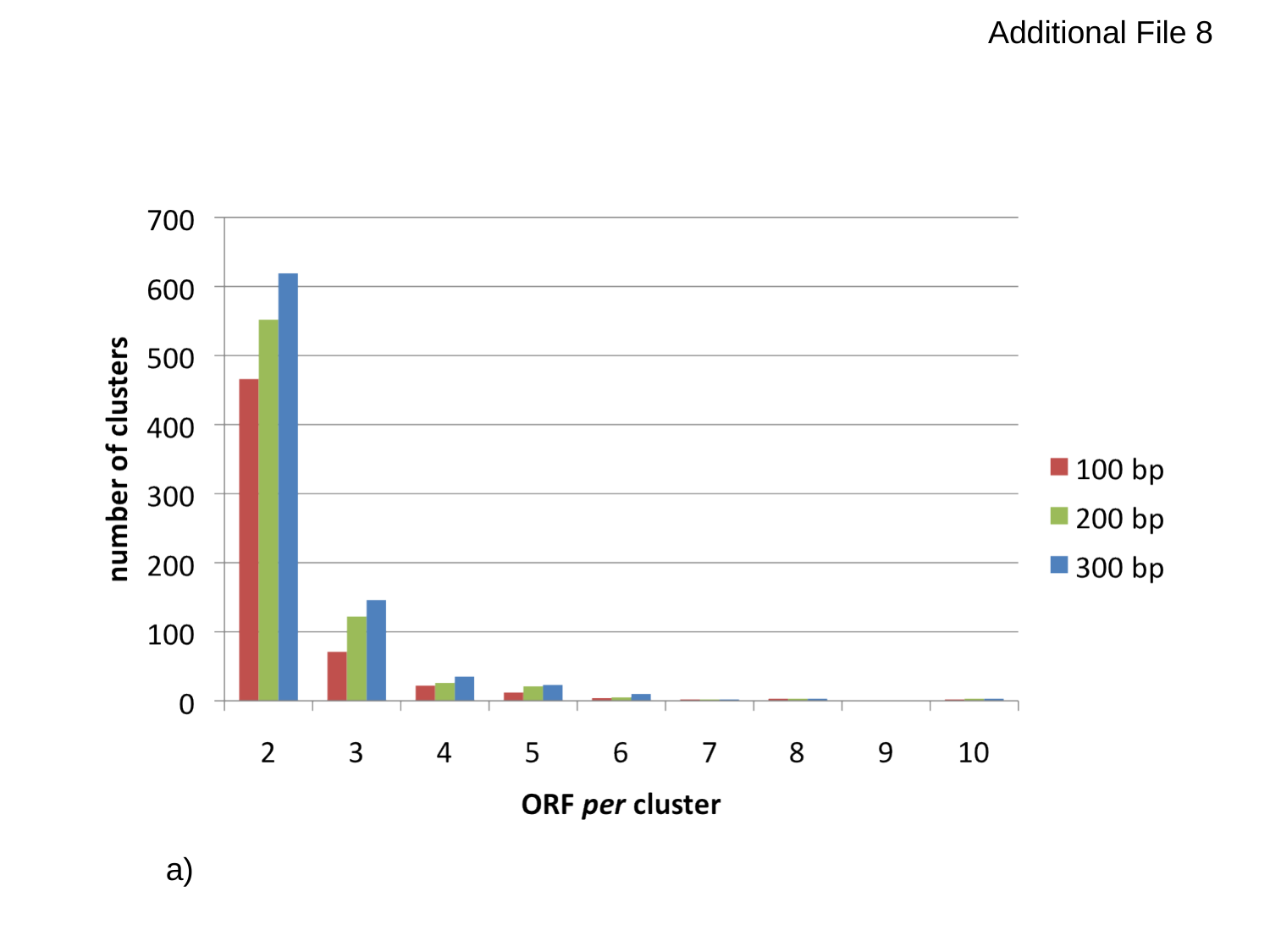

Additional File 8
a)

## Slide 2
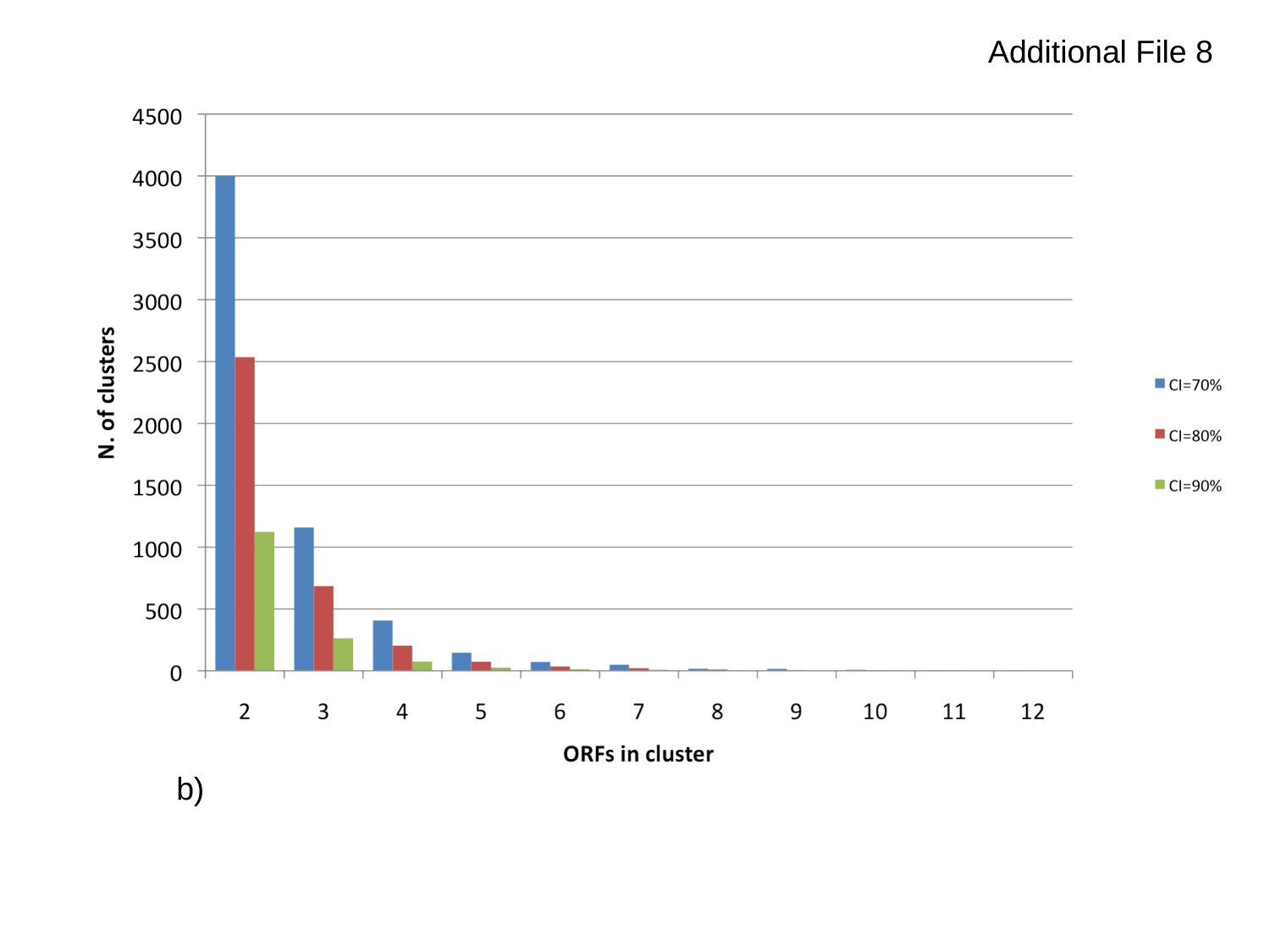

Additional File 8
b)

Supplement: Additional file 8 — clusters of lower confidence PAGs. Results of gene clusters analysis for a) 100, 200 and 300 bp gene distance threshold and b) for PAGs retrieved at 70%, 80% and 90% CIs. [file 1471-2164-12-403-S8.PPT]

## Slide 1
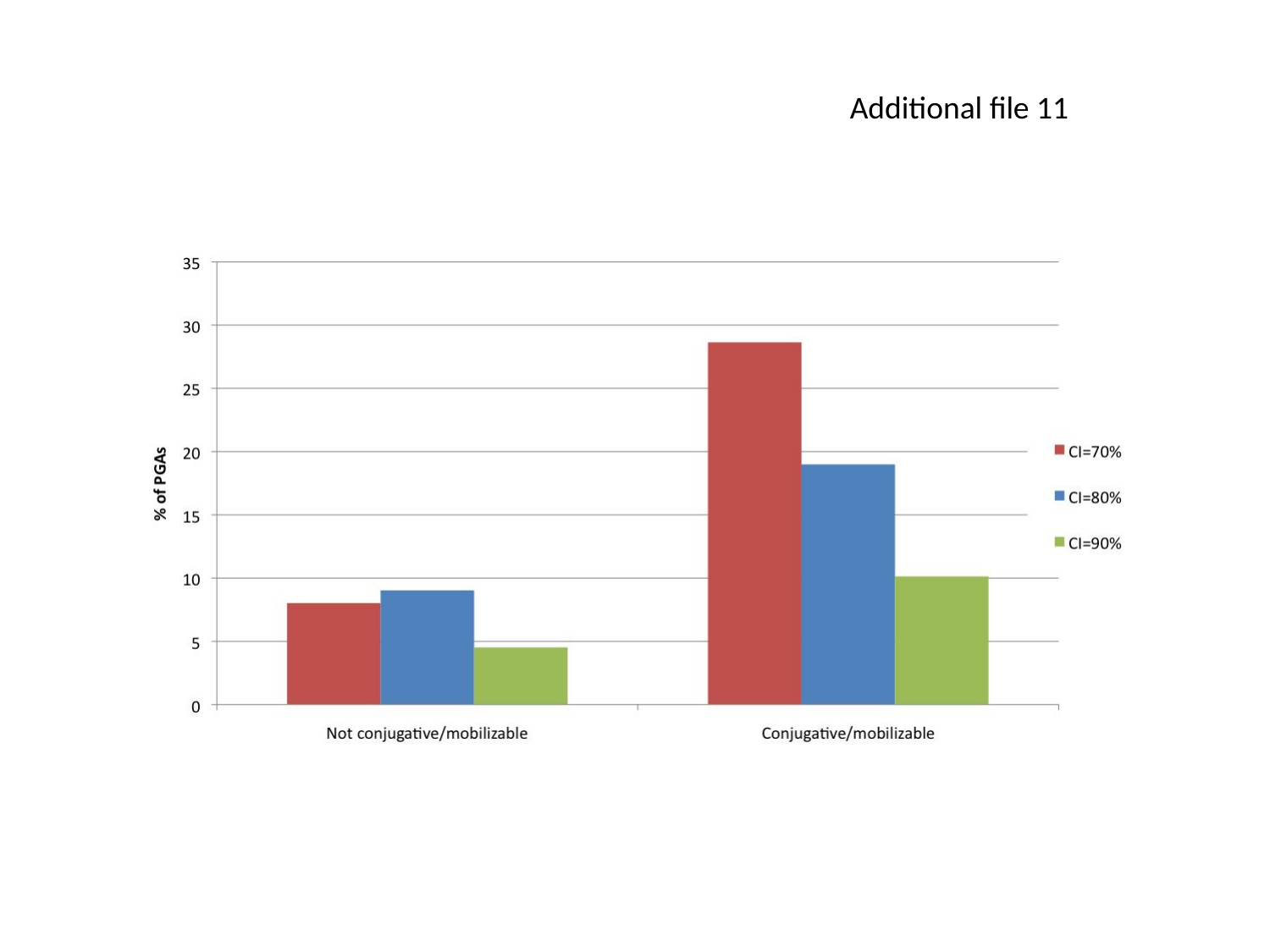

Additional file 11

Supplement: Additional file 11 — lower confidence PAGs in CMPs and NCMPs. Distribution of lower confidence PAGs among CMPs and NCMPs [file 1471-2164-12-403-S11.PPT]

## Slide 1
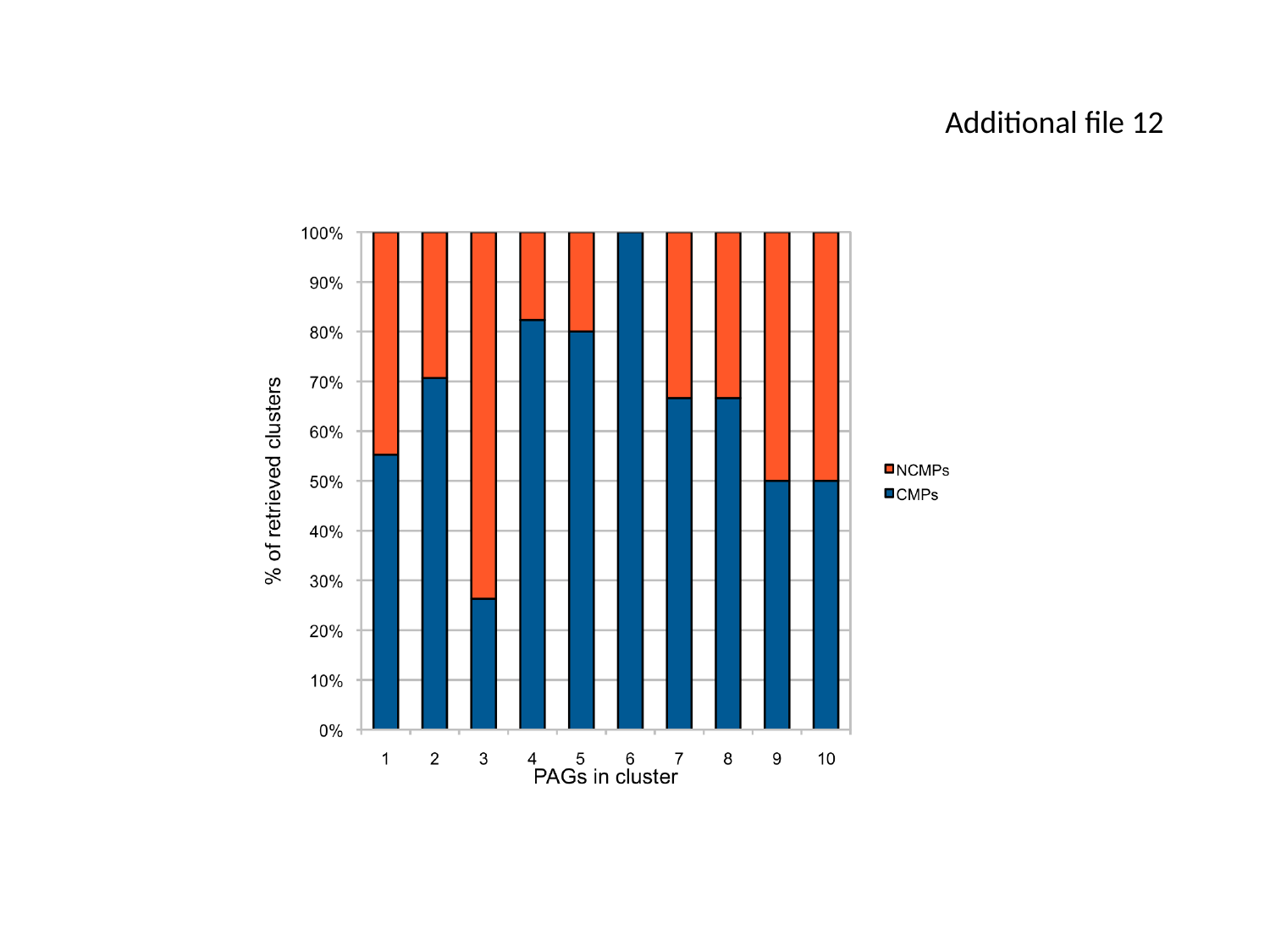

Additional file 12

Supplement: Additional file 12 — PAGs clusters in CMPs and NCMPs. Distribution and length of PAGs clusters in CMPs and NCMPs. [file 1471-2164-12-403-S12.PPT]
