# Supplementary material for: The mosaicism of plasmids revealed by atypical genes detection and analysis
Source: BMC Genomics. 2011 Aug 8;12:403. doi: 10.1186/1471-2164-12-403 (PMC3166947; doi:10.1186/1471-2164-12-403)
Supplement: Additional file 5 — lower confidence PAGs and plasmids size for lower CI values. Scatterplots illustrating the low positive correlation existing between plasmids size and their PAGs content, with three different PAGs datasets retrieved at a) 70%, b) 80% and c) 90%. [file 1471-2164-12-403-S5.PPT]

## Slide 1
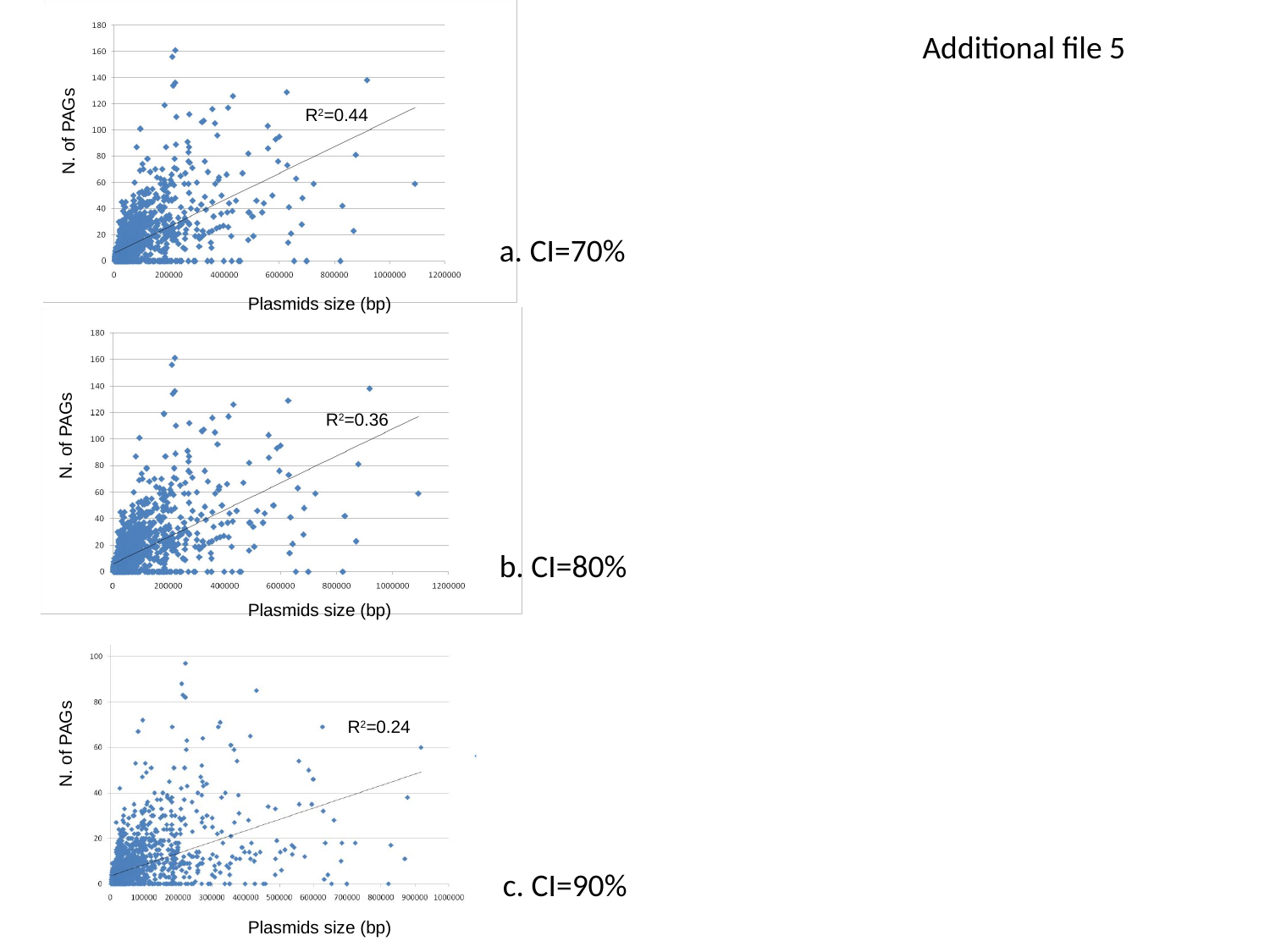

Additional file 5
R2=0.44
N. of PAGs
a. CI=70%
Plasmids size (bp)
R2=0.36
N. of PAGs
b. CI=80%
Plasmids size (bp)
R2=0.24
N. of PAGs
c. CI=90%
Plasmids size (bp)
