# Supplementary material for: The mosaicism of plasmids revealed by atypical genes detection and analysis
Source: BMC Genomics. 2011 Aug 8;12:403. doi: 10.1186/1471-2164-12-403 (PMC3166947; doi:10.1186/1471-2164-12-403)
Supplement: Additional file 6 — lower confidence PAGs functional annotation. a) Full COG http://www.ncbi.nih.gov/COG functional annotation of PAGs retrieved at 70%, 80% and 90% Cis. In particular, the function each of the sequences embedded in these datasets was inferred according to the one assigned to the best BLAST hit of COG database. b) Full Blast2GO functional annotation of PAGs retrieved at 95% CI. [file 1471-2164-12-403-S6.DOC]

Additional File 6

a)


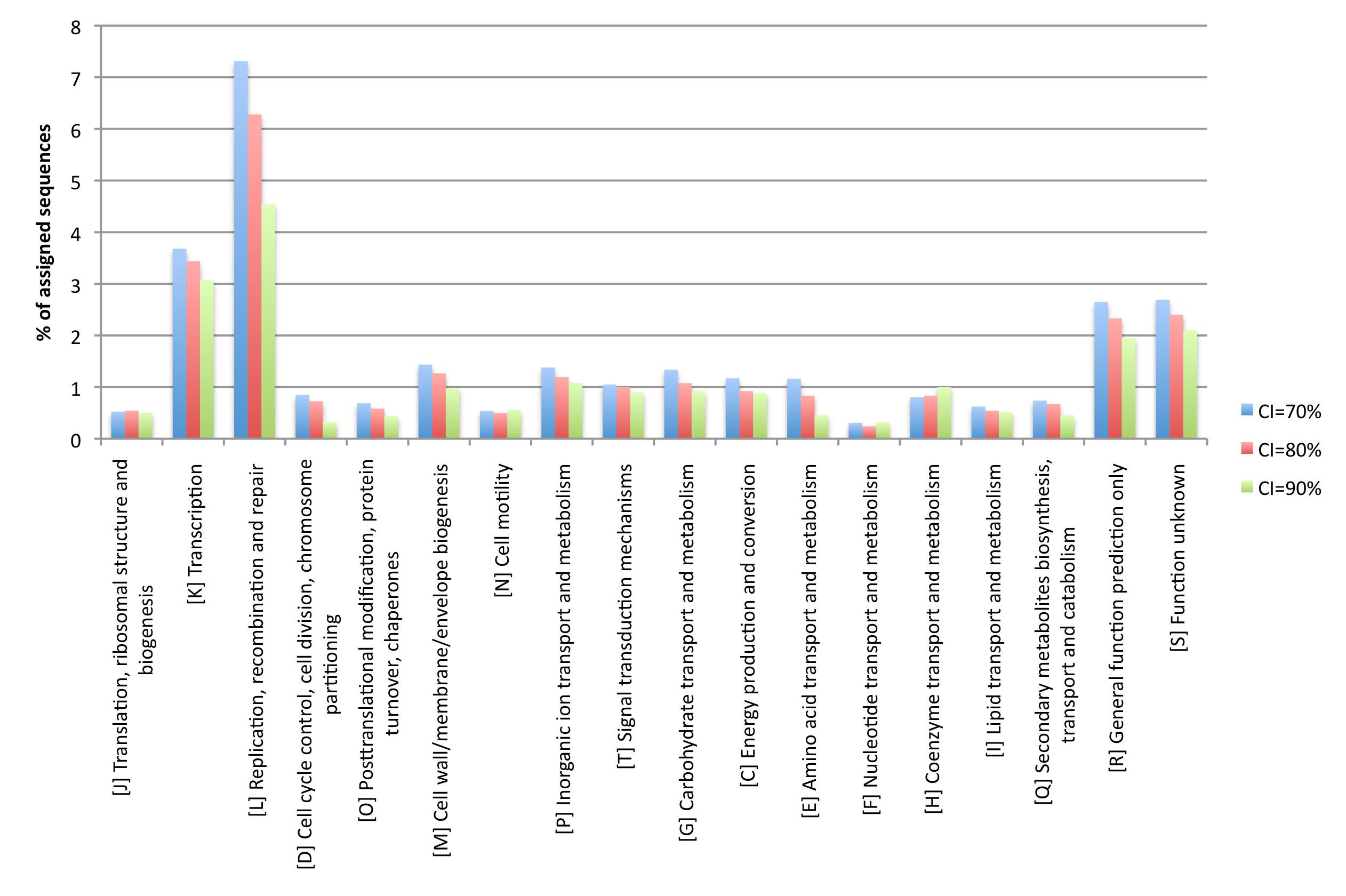


| **Sequence Distribution** | | | | |
| --- | --- | --- | --- | --- |
| **Graph Level** | **GO Term** | **#Seq** | **Score** | **Parents** |
| 1 | biological_process | 1962 | 547,66 |  |
| 6 | DNA metabolic process | 499 | 391,98 | nucleic acid metabolic process, cellular macromolecule metabolic process |
| 5 | nucleic acid metabolic process | 878 | 366,11 | nucleobase, nucleoside, nucleotide and nucleic acid metabolic process |
| 4 | cellular macromolecule metabolic process | 940 | 344,01 | cellular metabolic process, macromolecule metabolic process |
| 2 | metabolic process | 1490 | 330,19 | biological_process |
| 7 | regulation of transcription | 435 | 312,05 | transcription, regulation of macromolecule biosynthetic process, regulation of gene expression, regulation of cellular biosynthetic process, regulation of nucleobase, nucleoside, nucleotide and nucleic acid metabolic process |
| 2 | cellular process | 1439 | 307,04 | biological_process |
| 8 | regulation of transcription, DNA-dependent | 302 | 290,42 | transcription, DNA-dependent, regulation of RNA metabolic process, regulation of transcription |
| 3 | cellular metabolic process | 1277 | 267,41 | metabolic process, cellular process |
| 3 | macromolecule metabolic process | 1076 | 265,55 | metabolic process |
| 6 | DNA recombination | 321 | 239,40 | DNA metabolic process |
| 4 | nucleobase, nucleoside, nucleotide and nucleic acid metabolic process | 938 | 234,49 | primary metabolic process, cellular nitrogen compound metabolic process |
| 2 | multi-organism process | 380 | 230,21 | biological_process |
| 6 | DNA integration | 220 | 220,00 | DNA metabolic process |
| 6 | transcription | 446 | 210,44 | cellular macromolecule biosynthetic process, gene expression, nucleic acid metabolic process |
| 3 | primary metabolic process | 1144 | 210,33 | metabolic process |
| 4 | cellular biosynthetic process | 774 | 206,13 | cellular metabolic process, biosynthetic process |
| 4 | transposition, DNA-mediated | 204 | 204,00 | DNA recombination, transposition |
| 6 | transcription, DNA-dependent | 321 | 202,94 | transcription, RNA biosynthetic process |
| 6 | regulation of gene expression | 462 | 197,09 | gene expression, regulation of macromolecule metabolic process |
| 4 | transport | 337 | 194,65 | establishment of localization |
| 3 | pathogenesis | 190 | 190,00 | multi-organism process |
| 6 | regulation of macromolecule biosynthetic process | 437 | 188,67 | macromolecule biosynthetic process, regulation of macromolecule metabolic process, regulation of biosynthetic process |
| 6 | regulation of cellular biosynthetic process | 437 | 188,67 | cellular biosynthetic process, regulation of cellular metabolic process, regulation of biosynthetic process |
| 6 | regulation of nucleobase, nucleoside, nucleotide and nucleic acid metabolic process | 435 | 187,23 | nucleobase, nucleoside, nucleotide and nucleic acid metabolic process, regulation of nitrogen compound metabolic process, regulation of primary metabolic process, regulation of cellular metabolic process |
| 3 | conjugation | 195 | 185,00 | multi-organism process |
| 5 | cellular macromolecule biosynthetic process | 545 | 178,19 | cellular macromolecule metabolic process, cellular biosynthetic process, macromolecule biosynthetic process |
| 3 | biosynthetic process | 875 | 175,18 | metabolic process |
| 7 | regulation of RNA metabolic process | 302 | 174,25 | RNA metabolic process, regulation of nucleobase, nucleoside, nucleotide and nucleic acid metabolic process, regulation of macromolecule metabolic process |
| 4 | macromolecule biosynthetic process | 651 | 173,77 | macromolecule metabolic process, biosynthetic process |
| 4 | cellular nitrogen compound metabolic process | 1056 | 171,98 | cellular metabolic process, nitrogen compound metabolic process |
| 5 | regulation of macromolecule metabolic process | 462 | 160,08 | regulation of metabolic process, macromolecule metabolic process |
| 4 | response to antibiotic | 145 | 145,00 | response to chemical stimulus |
| 4 | gene expression | 493 | 141,97 | macromolecule metabolic process |
| 3 | transposition | 213 | 131,40 | cellular process |
| 3 | nitrogen compound metabolic process | 1073 | 130,71 | metabolic process |
| 5 | RNA biosynthetic process | 321 | 124,16 | RNA metabolic process, cellular biosynthetic process |
| 4 | regulation of cellular process | 501 | 123,63 | regulation of biological process, cellular process |
| 3 | response to chemical stimulus | 248 | 119,25 | response to stimulus |
| 3 | establishment of localization | 337 | 116,79 | biological_process, localization |
| 2 | response to stimulus | 357 | 116,41 | biological_process |
| 5 | regulation of cellular metabolic process | 437 | 113,20 | regulation of metabolic process, cellular metabolic process, regulation of cellular process |
| 5 | regulation of biosynthetic process | 437 | 113,20 | regulation of metabolic process, biosynthetic process |
| 5 | regulation of primary metabolic process | 437 | 112,86 | regulation of metabolic process, primary metabolic process |
| 5 | regulation of nitrogen compound metabolic process | 435 | 112,34 | regulation of metabolic process, nitrogen compound metabolic process |
| 5 | acyl-carrier-protein biosynthetic process | 104 | 104,00 | macromolecule biosynthetic process |
| 5 | auxin biosynthetic process | 103 | 103,00 | auxin metabolic process, cellular biosynthetic process, hormone biosynthetic process |
| 3 | oxidation reduction | 108 | 101,88 | metabolic process |
| 6 | RNA metabolic process | 337 | 82,85 | nucleic acid metabolic process, cellular macromolecule metabolic process |
| 3 | regulation of biological process | 528 | 76,59 | biological regulation, biological_process |
| 4 | signal transmission | 83 | 71,06 | signaling process |
| 4 | regulation of metabolic process | 462 | 70,95 | metabolic process, regulation of biological process |
| 2 | localization | 339 | 70,50 | biological_process |
| 5 | two-component signal transduction system (phosphorelay) | 67 | 67,00 | signal transmission |
| 6 | folic acid biosynthetic process | 67 | 67,00 | folic acid and derivative biosynthetic process, folic acid metabolic process, pteridine and derivative biosynthetic process, cellular amino acid biosynthetic process, water-soluble vitamin biosynthetic process |
| 3 | plasmid maintenance | 72 | 64,40 | cellular process |
| 5 | cellular amino acid metabolic process | 117 | 63,63 | cellular amine metabolic process, cellular amino acid and derivative metabolic process, carboxylic acid metabolic process |
| 4 | auxin metabolic process | 103 | 61,80 | cellular metabolic process, hormone metabolic process |
| 4 | hormone biosynthetic process | 103 | 61,80 | hormone metabolic process, biosynthetic process |
| 6 | DNA replication | 97 | 60,16 | cellular macromolecule biosynthetic process, DNA metabolic process |
| 6 | response to mercury ion | 80 | 57,36 | response to metal ion |
| 3 | small molecule metabolic process | 283 | 52,61 | metabolic process |
| 2 | biological regulation | 616 | 52,10 | biological_process |
| 5 | signal transduction | 53 | 51,43 | regulation of cellular process, signal transmission |
| 6 | cellular amino acid biosynthetic process | 77 | 50,34 | amine biosynthetic process, cellular amino acid metabolic process, carboxylic acid biosynthetic process |
| 4 | heterocycle metabolic process | 180 | 50,25 | cellular metabolic process |
| 6 | pteridine and derivative biosynthetic process | 73 | 48,00 | pteridine and derivative metabolic process, aromatic compound biosynthetic process, heterocycle biosynthetic process, cellular nitrogen compound biosynthetic process |
| 6 | water-soluble vitamin biosynthetic process | 80 | 47,76 | water-soluble vitamin metabolic process, vitamin biosynthetic process |
| 6 | carboxylic acid metabolic process | 149 | 47,52 | oxoacid metabolic process |
| 6 | folic acid and derivative biosynthetic process | 69 | 47,00 | folic acid and derivative metabolic process, coenzyme biosynthetic process |
| 6 | cation transport | 96 | 46,99 | ion transport |
| 9 | DNA replication initiation | 46 | 46,00 | DNA metabolic process, DNA-dependent DNA replication |
| 4 | cellular aromatic compound metabolic process | 128 | 45,88 | cellular metabolic process |
| 6 | carboxylic acid biosynthetic process | 104 | 44,62 | organic acid biosynthetic process, carboxylic acid metabolic process |
| 5 | transmembrane transport | 51 | 44,07 | transport, cellular process |
| null | electron transport | 44 | 44,00 | obsolete_biological_process |
| 5 | bacteriocin immunity | 44 | 44,00 | toxin metabolic process |
| 7 | metal ion transport | 66 | 42,85 | cation transport |
| 7 | protein secretion | 45 | 42,80 | secretion by cell, protein transport |
| 5 | cellular nitrogen compound biosynthetic process | 145 | 42,68 | cellular biosynthetic process, cellular nitrogen compound metabolic process |
| 3 | signaling process | 83 | 42,64 | biological_process, signaling |
| 3 | response to stress | 82 | 41,50 | response to stimulus |
| 5 | proteolysis | 41 | 41,00 | protein metabolic process |
| 5 | cellular amine metabolic process | 124 | 40,77 | cellular nitrogen compound metabolic process, amine metabolic process |
| 4 | cellular amino acid and derivative metabolic process | 119 | 40,27 | cellular metabolic process, primary metabolic process, small molecule metabolic process |
| 6 | folic acid metabolic process | 67 | 40,20 | pteridine and derivative metabolic process, water-soluble vitamin metabolic process, folic acid and derivative metabolic process, cellular amino acid metabolic process |
| 4 | protein metabolic process | 102 | 39,44 | primary metabolic process, macromolecule metabolic process |
| null | obsolete_biological_process | 62 | 38,40 |  |
| 3 | immune response | 40 | 37,45 | response to stimulus, immune system process |
| 4 | toxin metabolic process | 55 | 37,40 | cellular metabolic process, secondary metabolic process |
| 3 | hormone metabolic process | 103 | 37,08 | regulation of hormone levels, metabolic process |
| 5 | heterocycle biosynthetic process | 98 | 36,89 | heterocycle metabolic process, cellular biosynthetic process |
| 5 | protein transport | 57 | 36,76 | establishment of protein localization, transport |
| 6 | DNA repair | 37 | 36,20 | response to DNA damage stimulus, DNA metabolic process |
| 6 | phosphoenolpyruvate-dependent sugar phosphotransferase system | 36 | 36,00 | carbohydrate transport |
| 5 | detoxification of mercury ion | 44 | 35,60 | response to toxin, response to mercury ion |
| 5 | response to metal ion | 80 | 34,42 | response to inorganic substance |
| 7 | monocarboxylic acid metabolic process | 103 | 34,38 | carboxylic acid metabolic process |
| 5 | ion transport | 110 | 33,65 | transport |
| 8 | DNA-dependent DNA replication | 53 | 33,60 | DNA replication |
| 6 | coenzyme biosynthetic process | 77 | 32,28 | coenzyme metabolic process, cofactor biosynthetic process |
| 4 | cell redox homeostasis | 31 | 31,00 | cellular homeostasis, regulation of cellular process |
| 5 | amine biosynthetic process | 77 | 30,20 | cellular nitrogen compound biosynthetic process, amine metabolic process |
| 5 | DNA modification | 49 | 30,20 | macromolecule modification, DNA metabolic process |
| 5 | aromatic compound biosynthetic process | 75 | 29,95 | cellular aromatic compound metabolic process, cellular biosynthetic process |
| 8 | negative regulation of transcription | 40 | 29,60 | regulation of transcription, negative regulation of nucleobase, nucleoside, nucleotide and nucleic acid metabolic process, transcription, negative regulation of cellular biosynthetic process, negative regulation of macromolecule biosynthetic process, negative regulation of gene expression |
| 5 | water-soluble vitamin metabolic process | 82 | 29,59 | vitamin metabolic process |
| 9 | ATP biosynthetic process | 33 | 29,08 | purine ribonucleoside triphosphate biosynthetic process, ATP metabolic process |
| 4 | pteridine and derivative metabolic process | 73 | 28,80 | heterocycle metabolic process, nitrogen compound metabolic process, cellular aromatic compound metabolic process |
| 5 | vitamin biosynthetic process | 80 | 28,66 | small molecule biosynthetic process, vitamin metabolic process, cellular biosynthetic process |
| 5 | oxoacid metabolic process | 149 | 28,51 | cellular ketone metabolic process, organic acid metabolic process |
| 5 | folic acid and derivative metabolic process | 69 | 28,20 | heterocycle metabolic process, cellular aromatic compound metabolic process, monocarboxylic acid metabolic process, group transfer coenzyme metabolic process |
| 4 | macromolecule modification | 78 | 28,14 | macromolecule metabolic process |
| 4 | amine metabolic process | 130 | 27,84 | nitrogen compound metabolic process |
| 3 | secondary metabolic process | 62 | 27,52 | metabolic process |
| 5 | organic acid biosynthetic process | 104 | 26,77 | small molecule biosynthetic process, organic acid metabolic process, cellular biosynthetic process |
| 5 | cofactor biosynthetic process | 92 | 26,45 | cofactor metabolic process, cellular biosynthetic process |
| 6 | nucleobase metabolic process | 33 | 26,33 | heterocycle metabolic process, nucleobase, nucleoside and nucleotide metabolic process, cellular aromatic compound metabolic process |
| 4 | small molecule biosynthetic process | 157 | 26,04 | biosynthetic process, small molecule metabolic process |
| 8 | negative regulation of transcription, DNA-dependent | 26 | 26,00 | negative regulation of RNA metabolic process, regulation of transcription, DNA-dependent, transcription, DNA-dependent, negative regulation of transcription |
| 5 | secretion by cell | 45 | 25,68 | secretion, establishment of localization in cell, cellular process |
| 2 | signaling | 83 | 25,61 | biological_process |
| 7 | DNA methylation | 25 | 25,00 | regulation of gene expression, epigenetic, macromolecule methylation, DNA alkylation |
| 6 | purine base metabolic process | 29 | 23,88 | nucleobase metabolic process |
| 4 | nucleobase, nucleoside and nucleotide metabolic process | 93 | 23,70 | nucleobase, nucleoside, nucleotide and nucleic acid metabolic process, small molecule metabolic process |
| 4 | response to toxin | 46 | 23,36 | response to chemical stimulus |
| 4 | DNA topological change | 23 | 23,00 | DNA metabolic process, DNA conformation change |
| 4 | cytolysis | 23 | 23,00 | cell death |
| 3 | regulation of biological quality | 159 | 22,56 | biological regulation |
| 5 | carbohydrate transport | 37 | 22,54 | transport |
| 2 | immune system process | 40 | 22,47 | biological_process |
| 4 | regulation of hormone levels | 103 | 22,25 | regulation of biological quality |
| 8 | transition metal ion transport | 34 | 22,08 | metal ion transport |
| 5 | methylation | 39 | 22,08 | one-carbon metabolic process |
| 5 | establishment of protein localization | 57 | 22,05 | establishment of localization, protein localization |
| 9 | regulation of pH | 22 | 22,00 | monovalent inorganic cation homeostasis |
| 5 | response to DNA damage stimulus | 37 | 21,72 | cellular response to stress |
| 5 | cellular protein metabolic process | 62 | 21,40 | protein metabolic process, cellular macromolecule metabolic process |
| 7 | mercury ion transport | 21 | 21,00 | detoxification of mercury ion, di-, tri-valent inorganic cation transport, transition metal ion transport |
| 4 | response to inorganic substance | 81 | 20,78 | response to chemical stimulus |
| 6 | pyrimidine base metabolic process | 20 | 20,00 | nucleobase metabolic process |
| 7 | tetracycline transport | 20 | 20,00 | organic alcohol transport, antibiotic transport |
| 4 | cellular ketone metabolic process | 151 | 19,82 | cellular metabolic process, small molecule metabolic process |
| 4 | lipid biosynthetic process | 30 | 19,54 | lipid metabolic process, biosynthetic process |
| 4 | one-carbon metabolic process | 45 | 19,25 | cellular metabolic process, small molecule metabolic process |
| 4 | plasmid partitioning | 19 | 19,00 | plasmid maintenance |
| 5 | hemolysis of cells in other organism | 19 | 19,00 | cytolysis of cells of another organism |
| 3 | cellular homeostasis | 32 | 18,68 | homeostatic process, cellular process |
| 7 | di-, tri-valent inorganic cation transport | 32 | 18,48 | cation transport |
| 4 | lipid metabolic process | 41 | 18,18 | primary metabolic process |
| 4 | unidirectional conjugation | 18 | 18,00 | conjugation, genetic transfer |
| 4 | cellular catabolic process | 52 | 17,97 | cellular metabolic process, catabolic process |
| 7 | negative regulation of nucleobase, nucleoside, nucleotide and nucleic acid metabolic process | 40 | 17,76 | negative regulation of nitrogen compound metabolic process, negative regulation of cellular metabolic process, nucleobase, nucleoside, nucleotide and nucleic acid metabolic process, regulation of nucleobase, nucleoside, nucleotide and nucleic acid metabolic process |
| 4 | vitamin metabolic process | 82 | 17,76 | small molecule metabolic process |
| 7 | negative regulation of macromolecule biosynthetic process | 40 | 17,76 | regulation of macromolecule biosynthetic process, macromolecule biosynthetic process, negative regulation of macromolecule metabolic process, negative regulation of biosynthetic process |
| 7 | negative regulation of cellular biosynthetic process | 40 | 17,76 | cellular biosynthetic process, regulation of cellular biosynthetic process, negative regulation of cellular metabolic process, negative regulation of biosynthetic process |
| 7 | negative regulation of gene expression | 40 | 17,76 | gene expression, regulation of gene expression, negative regulation of macromolecule metabolic process |
| 8 | purine ribonucleoside triphosphate biosynthetic process | 33 | 17,45 | ribonucleoside triphosphate biosynthetic process, purine ribonucleoside triphosphate metabolic process, purine ribonucleotide biosynthetic process, purine nucleoside triphosphate biosynthetic process |
| 8 | ATP metabolic process | 33 | 17,45 | purine ribonucleoside triphosphate metabolic process |
| 4 | organic acid metabolic process | 149 | 17,19 | cellular metabolic process, small molecule metabolic process |
| 6 | translation | 22 | 16,96 | cellular macromolecule biosynthetic process, gene expression, cellular protein metabolic process |
| 6 | group transfer coenzyme metabolic process | 69 | 16,92 | coenzyme metabolic process |
| 6 | phosphorylation | 31 | 16,90 | phosphate metabolic process |
| 6 | macromolecule methylation | 27 | 16,80 | macromolecule modification, cellular macromolecule metabolic process, methylation |
| 3 | DNA conformation change | 29 | 16,42 | cellular process |
| 8 | branched chain family amino acid catabolic process | 9 | 16,20 | cellular amino acid catabolic process, branched chain family amino acid metabolic process |
| 4 | cofactor metabolic process | 107 | 16,16 | cellular metabolic process |
| 7 | transcription initiation | 16 | 16,00 | transcription, DNA-dependent, protein complex assembly |
| 7 | negative regulation of RNA metabolic process | 26 | 15,60 | regulation of RNA metabolic process, negative regulation of nucleobase, nucleoside, nucleotide and nucleic acid metabolic process, RNA metabolic process, negative regulation of macromolecule metabolic process |
| 4 | establishment of localization in cell | 46 | 15,45 | establishment of localization, cellular localization |
| 5 | secretion | 45 | 15,41 | transport |
| 3 | cell death | 26 | 15,25 | death, cellular process |
| 6 | DNA alkylation | 25 | 15,00 | DNA modification |
| 7 | regulation of gene expression, epigenetic | 25 | 15,00 | regulation of gene expression |
| 6 | nucleotide metabolic process | 60 | 14,84 | nucleoside phosphate metabolic process |
| 3 | catabolic process | 64 | 14,58 | metabolic process |
| 6 | negative regulation of macromolecule metabolic process | 40 | 14,40 | macromolecule metabolic process, regulation of macromolecule metabolic process, negative regulation of metabolic process |
| 4 | cellular response to stress | 39 | 14,16 | response to stress, cellular response to stimulus |
| 5 | coenzyme metabolic process | 89 | 14,01 | cofactor metabolic process |
| 6 | proton transport | 19 | 13,45 | hydrogen transport, monovalent inorganic cation transport |
| 4 | ribosome biogenesis | 16 | 13,30 | ribonucleoprotein complex biogenesis |
| 4 | protein localization | 57 | 13,23 | macromolecule localization |
| 8 | monovalent inorganic cation homeostasis | 22 | 13,20 | cation homeostasis |
| 4 | generation of precursor metabolites and energy | 26 | 13,20 | cellular metabolic process |
| 4 | electron transport chain | 14 | 13,13 | generation of precursor metabolites and energy, oxidation reduction |
| 6 | fatty acid biosynthetic process | 13 | 13,00 | lipid biosynthetic process, fatty acid metabolic process, carboxylic acid biosynthetic process |
| null | ATP-dependent proteolysis | 13 | 13,00 | obsolete_biological_process |
| 6 | antibiotic transport | 21 | 13,00 | drug transport |
| 4 | homeostatic process | 54 | 12,92 | regulation of biological quality |
| 6 | nucleotide biosynthetic process | 45 | 12,49 | nucleotide metabolic process, nucleobase, nucleoside and nucleotide biosynthetic process |
| 7 | protein amino acid phosphorylation | 15 | 12,20 | phosphorylation, post-translational protein modification |
| 5 | negative regulation of cellular process | 52 | 12,10 | cellular process, regulation of cellular process, negative regulation of biological process |
| 6 | serine family amino acid metabolic process | 18 | 12,04 | cellular amino acid metabolic process |
| 5 | organic alcohol transport | 20 | 12,00 | transport |
| 6 | DNA restriction-modification system | 12 | 12,00 | DNA modification |
| 4 | cellular lipid metabolic process | 32 | 11,97 | cellular metabolic process, lipid metabolic process |
| 6 | branched chain family amino acid metabolic process | 11 | 11,88 | cellular amino acid metabolic process |
| 6 | cellular amino acid catabolic process | 12 | 11,74 | cellular amino acid metabolic process, amine catabolic process, carboxylic acid catabolic process |
| 7 | monovalent inorganic cation transport | 24 | 11,67 | cation transport |
| 4 | carbohydrate metabolic process | 42 | 11,60 | primary metabolic process |
| 4 | cytolysis of cells of another organism | 19 | 11,40 | killing of cells of another organism |
| 5 | porphyrin metabolic process | 17 | 11,28 | tetrapyrrole metabolic process, cofactor metabolic process |
| 2 | cellular component biogenesis | 51 | 11,15 | biological_process |
| 5 | protein complex assembly | 19 | 11,04 | macromolecular complex assembly, protein complex biogenesis |
| 7 | cobalamin biosynthetic process | 11 | 11,00 | porphyrin biosynthetic process, water-soluble vitamin biosynthetic process, cobalamin metabolic process |
| 6 | post-translational protein modification | 23 | 10,92 | protein modification process |
| 6 | glutamine family amino acid metabolic process | 15 | 10,92 | cellular amino acid metabolic process |
| 5 | phosphate metabolic process | 33 | 10,86 | phosphorus metabolic process |
| 3 | genetic transfer | 18 | 10,80 | multi-organism process |
| 7 | purine ribonucleotide biosynthetic process | 34 | 10,68 | purine nucleotide biosynthetic process, ribonucleotide biosynthetic process, purine ribonucleotide metabolic process |
| 4 | cellular carbohydrate metabolic process | 26 | 10,67 | cellular metabolic process, carbohydrate metabolic process |
| 6 | negative regulation of cellular metabolic process | 40 | 10,66 | negative regulation of cellular process, regulation of cellular metabolic process, cellular metabolic process, negative regulation of metabolic process |
| 6 | negative regulation of nitrogen compound metabolic process | 40 | 10,66 | nitrogen compound metabolic process, regulation of nitrogen compound metabolic process, negative regulation of metabolic process |
| 6 | negative regulation of biosynthetic process | 40 | 10,66 | biosynthetic process, negative regulation of metabolic process, regulation of biosynthetic process |
| 8 | ribonucleoside triphosphate biosynthetic process | 33 | 10,47 | ribonucleoside triphosphate metabolic process, nucleoside triphosphate biosynthetic process, ribonucleotide biosynthetic process |
| 7 | purine nucleoside triphosphate biosynthetic process | 33 | 10,47 | purine nucleoside triphosphate metabolic process, purine nucleotide biosynthetic process, nucleoside triphosphate biosynthetic process |
| 7 | purine ribonucleoside triphosphate metabolic process | 33 | 10,47 | ribonucleoside triphosphate metabolic process, purine nucleoside triphosphate metabolic process, purine ribonucleotide metabolic process |
| 6 | polysaccharide biosynthetic process | 19 | 10,46 | polysaccharide metabolic process, carbohydrate biosynthetic process, macromolecule biosynthetic process |
| 8 | RNA-dependent DNA replication | 10 | 10,00 | DNA replication |
| 6 | porphyrin biosynthetic process | 14 | 9,80 | cofactor biosynthetic process, tetrapyrrole biosynthetic process, porphyrin metabolic process |
| 4 | sulfur metabolic process | 19 | 9,66 | cellular metabolic process |
| 6 | chlorophyll metabolic process | 10 | 9,60 | porphyrin metabolic process |
| 3 | cellular localization | 46 | 9,27 | localization, cellular process |
| 2 | cellular component organization | 43 | 9,26 | biological_process |
| 3 | cellular response to stimulus | 50 | 9,16 | response to stimulus, cellular process |
| 2 | death | 26 | 9,15 | biological_process |
| 8 | leucine catabolic process | 9 | 9,00 | branched chain family amino acid catabolic process, leucine metabolic process |
| 8 | valine catabolic process | 9 | 9,00 | branched chain family amino acid catabolic process, valine metabolic process |
| 8 | isoleucine catabolic process | 9 | 9,00 | isoleucine metabolic process, branched chain family amino acid catabolic process |
| 3 | cell adhesion | 9 | 9,00 | biological adhesion, cellular process |
| 6 | DNA catabolic process | 9 | 9,00 | DNA metabolic process, cellular macromolecule catabolic process |
| 6 | nucleoside phosphate metabolic process | 60 | 8,90 | nucleobase, nucleoside and nucleotide metabolic process |
| 6 | fatty acid metabolic process | 14 | 8,80 | cellular lipid metabolic process, monocarboxylic acid metabolic process |
| 4 | negative regulation of biological process | 54 | 8,62 | regulation of biological process, biological_process |
| 5 | antibiotic metabolic process | 16 | 8,47 | drug metabolic process |
| 5 | drug transport | 22 | 8,40 | response to drug, transport |
| 7 | glycine metabolic process | 12 | 8,40 | serine family amino acid metabolic process |
| 5 | antibiotic biosynthetic process | 16 | 8,32 | cellular biosynthetic process, antibiotic metabolic process |
| 7 | cation homeostasis | 23 | 8,14 | ion homeostasis |
| 6 | nucleobase, nucleoside and nucleotide biosynthetic process | 46 | 8,09 | nucleobase, nucleoside and nucleotide metabolic process, small molecule biosynthetic process, nucleobase, nucleoside, nucleotide and nucleic acid biosynthetic process |
| 5 | hydrogen transport | 19 | 8,07 | transport |
| 8 | adenine catabolic process | 8 | 8,00 | adenine metabolic process, purine base catabolic process |
| 10 | pentose-phosphate shunt | 8 | 8,00 | NADPH regeneration, glucose catabolic process |
| 4 | nitrogen fixation | 8 | 8,00 | nitrogen compound metabolic process |
| 4 | cellular cell wall organization | 8 | 8,00 | cell wall organization, cellular cell wall organization or biogenesis, external encapsulating structure organization |
| 6 | beta-alanine metabolic process | 8 | 8,00 | cellular amino acid metabolic process |
| 6 | dihydrofolate biosynthetic process | 8 | 8,00 | folic acid and derivative biosynthetic process, pteridine and derivative biosynthetic process, dihydrofolate metabolic process |
| 8 | glycine biosynthetic process | 8 | 8,00 | glycine metabolic process, serine family amino acid biosynthetic process |
| 7 | protein secretion by the type III secretion system | 8 | 8,00 | protein secretion |
| 3 | ribonucleoprotein complex biogenesis | 16 | 7,98 | cellular component biogenesis, cellular process |
| 3 | macromolecule localization | 57 | 7,94 | localization |
| 5 | cellular macromolecule catabolic process | 14 | 7,68 | cellular macromolecule metabolic process, cellular catabolic process, macromolecule catabolic process |
| 4 | carbohydrate biosynthetic process | 24 | 7,57 | biosynthetic process, carbohydrate metabolic process |
| 4 | polysaccharide metabolic process | 24 | 7,48 | macromolecule metabolic process, carbohydrate metabolic process |
| 4 | tetrapyrrole metabolic process | 18 | 7,49 | heterocycle metabolic process, nitrogen compound metabolic process |
| 3 | killing of cells of another organism | 20 | 7,44 | multi-organism process, cell killing |
| 6 | protein folding | 9 | 7,40 | cellular protein metabolic process |
| 6 | regulation of protein secretion | 10 | 7,20 | protein secretion, regulation of secretion, regulation of cellular localization, regulation of protein transport |
| 3 | cellular cell wall organization or biogenesis | 16 | 7,18 | cell wall organization or biogenesis, cellular process |
| 5 | tetrapyrrole biosynthetic process | 15 | 7,08 | tetrapyrrole metabolic process, heterocycle biosynthetic process, cellular nitrogen compound biosynthetic process |
| 6 | carboxylic acid catabolic process | 12 | 7,04 | carboxylic acid metabolic process, organic acid catabolic process |
| 5 | amine catabolic process | 12 | 7,04 | amine metabolic process |
| 8 | ribonucleotide biosynthetic process | 35 | 7,01 | nucleotide biosynthetic process, ribonucleotide metabolic process |
| 6 | penicillin biosynthetic process | 7 | 7,00 | penicillin metabolic process, beta-lactam antibiotic biosynthetic process, carboxylic acid biosynthetic process, sulfur compound biosynthetic process |
| 4 | conjugation with cellular fusion | 7 | 7,00 | sexual reproduction, conjugation |
| 7 | negative regulation of protein secretion | 7 | 7,00 | negative regulation of cellular process, protein secretion, regulation of protein secretion, negative regulation of protein transport, negative regulation of secretion |
| 8 | peptidyl-histidine phosphorylation | 7 | 7,00 | protein amino acid phosphorylation, peptidyl-histidine modification |
| 6 | protein modification process | 25 | 6,90 | macromolecule modification, cellular protein metabolic process |
| 6 | aspartate family amino acid metabolic process | 14 | 6,83 | cellular amino acid metabolic process |
| 7 | ATP synthesis coupled proton transport | 8 | 6,80 | ATP biosynthetic process, oxidative phosphorylation, energy coupled proton transport, down electrochemical gradient |
| 3 | cellular component assembly | 28 | 6,77 | cellular component biogenesis, cellular component organization |
| 4 | macromolecular complex assembly | 20 | 6,70 | macromolecular complex subunit organization, cellular component assembly |
| 6 | monosaccharide metabolic process | 17 | 6,71 | cellular carbohydrate metabolic process, alcohol metabolic process |
| 3 | protein complex biogenesis | 19 | 6,62 | cellular component biogenesis |
| 4 | peptidoglycan-based cell wall biogenesis | 9 | 6,60 | cell wall biogenesis |
| 7 | leucine metabolic process | 11 | 6,60 | branched chain family amino acid metabolic process |
| 7 | valine metabolic process | 11 | 6,60 | branched chain family amino acid metabolic process |
| 8 | glucose catabolic process | 11 | 6,60 | hexose catabolic process, glucose metabolic process |
| 7 | isoleucine metabolic process | 11 | 6,60 | branched chain family amino acid metabolic process |
| 6 | cobalamin metabolic process | 11 | 6,60 | water-soluble vitamin metabolic process, porphyrin metabolic process |
| 4 | phosphorus metabolic process | 33 | 6,52 | cellular metabolic process |
| 8 | nucleoside triphosphate biosynthetic process | 34 | 6,50 | nucleotide biosynthetic process, nucleoside triphosphate metabolic process |
| 6 | purine ribonucleotide metabolic process | 34 | 6,41 | ribonucleotide metabolic process, purine nucleotide metabolic process |
| 6 | purine nucleotide biosynthetic process | 34 | 6,41 | nucleotide biosynthetic process, purine nucleotide metabolic process |
| 6 | aromatic amino acid family metabolic process | 10 | 6,41 | cellular amino acid metabolic process, cellular aromatic compound metabolic process |
| 5 | negative regulation of metabolic process | 40 | 6,39 | regulation of metabolic process, metabolic process, negative regulation of biological process |
| 8 | glucose metabolic process | 12 | 6,36 | hexose metabolic process |
| 8 | ribonucleoside triphosphate metabolic process | 33 | 6,28 | ribonucleotide metabolic process, nucleoside triphosphate metabolic process |
| 6 | purine nucleoside triphosphate metabolic process | 33 | 6,28 | nucleoside triphosphate metabolic process, purine nucleotide metabolic process |
| 6 | polyketide biosynthetic process | 6 | 6,00 | ketone biosynthetic process, acetate derivative biosynthetic process, polyketide metabolic process |
| 9 | DNA unwinding involved in replication | 6 | 6,00 | DNA-dependent DNA replication, DNA duplex unwinding |
| 5 | glyoxylate metabolic process | 6 | 6,00 | monocarboxylic acid metabolic process, cellular aldehyde metabolic process |
| 8 | peptidoglycan biosynthetic process | 6 | 6,00 | glycosaminoglycan biosynthetic process, peptidoglycan-based cell wall biogenesis, cell wall macromolecule biosynthetic process, peptidoglycan metabolic process |
| 7 | organic anion transport | 7 | 6,00 | anion transport |
| 7 | aspartate metabolic process | 6 | 6,00 | aspartate family amino acid metabolic process |
| 6 | response to methotrexate | 6 | 6,00 | response to organic cyclic substance |
| null | peroxidase reaction | 6 | 6,00 | obsolete_biological_process |
| 8 | cadmium ion transport | 6 | 6,00 | di-, tri-valent inorganic cation transport, transition metal ion transport |
| 8 | phosphonate transport | 6 | 6,00 | organic anion transport |
| 7 | alanine metabolic process | 6 | 6,00 | pyruvate family amino acid metabolic process |
| 9 | copper ion transport | 6 | 6,00 | transition metal ion transport |
| 6 | hexose metabolic process | 13 | 5,98 | monosaccharide metabolic process |
| 5 | antibiotic catabolic process | 7 | 5,80 | cellular catabolic process, antibiotic metabolic process |
| 6 | anion transport | 13 | 5,76 | ion transport |
| 2 | cell wall organization or biogenesis | 20 | 5,75 | biological_process |
| 3 | cell division | 7 | 5,43 | cellular process |
| 2 | biological adhesion | 9 | 5,40 | biological_process |
| 4 | alcohol metabolic process | 21 | 5,33 | small molecule metabolic process |
| 5 | cellular amino acid derivative metabolic process | 13 | 5,27 | cellular amino acid and derivative metabolic process |
| 2 | cell killing | 22 | 5,18 | biological_process |
| 4 | response to oxidative stress | 6 | 5,13 | response to chemical stimulus, response to stress |
| 4 | drug metabolic process | 16 | 5,08 | cellular metabolic process |
| 5 | heterocycle catabolic process | 15 | 5,06 | heterocycle metabolic process, cellular catabolic process |
| 4 | response to drug | 22 | 5,04 | response to chemical stimulus |
| 6 | D-gluconate metabolic process | 5 | 5,00 | aldonic acid metabolic process |
| 9 | Mo-molybdopterin cofactor biosynthetic process | 5 | 5,00 | molybdopterin cofactor biosynthetic process, Mo-molybdopterin cofactor metabolic process, coenzyme biosynthetic process |
| 8 | benzoate metabolic process | 5 | 5,00 | monocarboxylic acid metabolic process, benzene and derivative metabolic process |
| 3 | spore germination | 5 | 5,00 | developmental process |
| 7 | glutamate metabolic process | 5 | 5,00 | glutamine family amino acid metabolic process |
| 4 | defense response | 9 | 5,01 | response to stress |
| 5 | taurine metabolic process | 5 | 5,00 | cellular amino acid derivative metabolic process, sulfur metabolic process |
| 6 | lipopolysaccharide biosynthetic process | 5 | 5,00 | lipopolysaccharide metabolic process, cellular polysaccharide biosynthetic process, lipid biosynthetic process |
| 7 | streptomycin biosynthetic process | 5 | 5,00 | aminoglycoside antibiotic biosynthetic process, streptomycin metabolic process |
| 6 | tryptophan metabolic process | 6 | 4,96 | indolalkylamine metabolic process, aromatic amino acid family metabolic process |
| 4 | macromolecule catabolic process | 16 | 4,95 | macromolecule metabolic process, catabolic process |
| 6 | ion homeostasis | 23 | 4,88 | chemical homeostasis |
| 5 | nucleobase, nucleoside, nucleotide and nucleic acid biosynthetic process | 46 | 4,86 | nucleobase, nucleoside, nucleotide and nucleic acid metabolic process, cellular nitrogen compound biosynthetic process |
| 3 | external encapsulating structure organization | 8 | 4,80 | cellular component organization |
| 8 | adenine metabolic process | 8 | 4,80 | purine base metabolic process |
| 6 | dihydrofolate metabolic process | 8 | 4,80 | pteridine and derivative metabolic process, folic acid and derivative metabolic process |
| 8 | serine family amino acid biosynthetic process | 8 | 4,80 | serine family amino acid metabolic process, cellular amino acid biosynthetic process |
| 8 | purine base catabolic process | 8 | 4,80 | nucleobase catabolic process, purine base metabolic process |
| 3 | cell wall organization | 8 | 4,80 | cell wall organization or biogenesis |
| 10 | NADPH regeneration | 8 | 4,80 | NADP metabolic process |
| 8 | positive regulation of transcription | 5 | 4,60 | regulation of transcription, transcription, positive regulation of cellular biosynthetic process, positive regulation of macromolecule biosynthetic process, positive regulation of nucleobase, nucleoside, nucleotide and nucleic acid metabolic process, positive regulation of gene expression |
| 6 | tyrosine metabolic process | 5 | 4,60 | aromatic amino acid family metabolic process |
| 7 | glutamine metabolic process | 5 | 4,60 | glutamine family amino acid metabolic process |
| 5 | sulfur compound biosynthetic process | 8 | 4,56 | cellular biosynthetic process, sulfur metabolic process |
| 4 | small molecule catabolic process | 27 | 4,56 | catabolic process, small molecule metabolic process |
| 8 | peptidyl-histidine modification | 8 | 4,56 | peptidyl-amino acid modification |
| 4 | phospholipid metabolic process | 7 | 4,54 | organophosphate metabolic process, cellular lipid metabolic process |
| 8 | pyruvate metabolic process | 6 | 4,40 | monocarboxylic acid metabolic process |
| 6 | phospholipid biosynthetic process | 5 | 4,36 | phospholipid metabolic process, lipid biosynthetic process, cellular biosynthetic process |
| 6 | regulation of protein transport | 10 | 4,32 | regulation of establishment of protein localization, protein transport, regulation of transport |
| 6 | cellular carbohydrate biosynthetic process | 11 | 4,32 | cellular carbohydrate metabolic process, cellular biosynthetic process, carbohydrate biosynthetic process |
| 6 | regulation of secretion | 10 | 4,32 | secretion, regulation of transport |
| 6 | beta-lactam antibiotic metabolic process | 7 | 4,32 | heterocycle metabolic process, cellular amine metabolic process, cellular amide metabolic process, antibiotic metabolic process |
| 5 | regulation of cellular localization | 10 | 4,32 | cellular localization, regulation of localization, regulation of cellular process |
| 5 | oxidative phosphorylation | 9 | 4,30 | phosphorylation, generation of precursor metabolites and energy |
| 5 | organic acid catabolic process | 12 | 4,22 | small molecule catabolic process, cellular catabolic process, organic acid metabolic process |
| 3 | sexual reproduction | 7 | 4,20 | reproduction |
| 8 | peptidoglycan metabolic process | 7 | 4,20 | glycosaminoglycan metabolic process |
| 7 | negative regulation of secretion | 7 | 4,20 | regulation of secretion, secretion, negative regulation of transport |
| 8 | tRNA aminoacylation for protein translation | 5 | 4,20 | translation, tRNA aminoacylation |
| 6 | beta-lactam antibiotic biosynthetic process | 7 | 4,20 | heterocycle biosynthetic process, amide biosynthetic process, antibiotic biosynthetic process, beta-lactam antibiotic metabolic process |
| 8 | ribonucleotide metabolic process | 35 | 4,21 | nucleotide metabolic process |
| 7 | negative regulation of protein transport | 7 | 4,20 | regulation of protein transport, negative regulation of transport, protein transport |
| 5 | penicillin metabolic process | 7 | 4,20 | carboxylic acid metabolic process, sulfur metabolic process, beta-lactam antibiotic metabolic process |
| 7 | energy coupled proton transport, down electrochemical gradient | 8 | 4,08 | proton transport, ion transmembrane transport |
| 3 | macromolecular complex subunit organization | 20 | 4,02 | cellular component organization |
| 8 | sodium ion transport | 4 | 4,00 | metal ion transport, monovalent inorganic cation transport |
| 4 | cell wall macromolecule catabolic process | 4 | 4,00 | cell wall macromolecule metabolic process, catabolic process |
| 4 | chemotaxis | 4 | 4,00 | response to chemical stimulus, taxis |
| 8 | molybdate ion transport | 4 | 4,00 | inorganic anion transport |
| 9 | DNA replication, synthesis of RNA primer | 4 | 4,00 | DNA metabolic process, DNA-dependent DNA replication, RNA biosynthetic process |
| 6 | amino sugar metabolic process | 4 | 4,00 | monosaccharide metabolic process |
| 5 | gas vesicle organization | 4 | 4,00 | vesicle organization |
| 5 | proline metabolic process | 4 | 4,00 | heterocycle metabolic process, glutamine family amino acid metabolic process |
| 7 | protein refolding | 4 | 4,00 | protein folding |
| 8 | starch metabolic process | 4 | 4,00 | cellular glucan metabolic process |
| 6 | sucrose metabolic process | 4 | 4,00 | glycoside metabolic process, disaccharide metabolic process |
| 8 | gluconeogenesis | 4 | 4,00 | pyruvate metabolic process, hexose biosynthetic process, glucose metabolic process |
| 3 | cell wall biogenesis | 9 | 3,96 | cellular cell wall organization or biogenesis, cellular component biogenesis |
| 8 | hexose catabolic process | 11 | 3,96 | hexose metabolic process, monosaccharide catabolic process |
| 8 | nucleoside triphosphate metabolic process | 34 | 3,90 | nucleotide metabolic process |
| 5 | purine nucleotide metabolic process | 34 | 3,85 | heterocycle metabolic process, nucleotide metabolic process |
| 5 | RNA modification | 5 | 3,80 | RNA metabolic process, macromolecule modification |
| 6 | RNA catabolic process | 5 | 3,80 | RNA metabolic process, cellular macromolecule catabolic process |
| 8 | iron ion transport | 5 | 3,80 | di-, tri-valent inorganic cation transport, transition metal ion transport |
| 4 | cell wall macromolecule metabolic process | 10 | 3,70 | cell wall organization or biogenesis, macromolecule metabolic process |
| 7 | inorganic anion transport | 6 | 3,60 | anion transport |
| 5 | ketone biosynthetic process | 6 | 3,60 | cellular ketone metabolic process, small molecule biosynthetic process, cellular biosynthetic process |
| 10 | polyketide metabolic process | 6 | 3,60 | acetate derivative metabolic process |
| 6 | cell wall macromolecule biosynthetic process | 6 | 3,60 | cell wall biogenesis, cellular cell wall macromolecule metabolic process, cellular component macromolecule biosynthetic process |
| 6 | cellular polysaccharide biosynthetic process | 6 | 3,60 | cellular macromolecule biosynthetic process, cellular polysaccharide metabolic process, polysaccharide biosynthetic process, cellular carbohydrate biosynthetic process |
| 6 | pyruvate family amino acid metabolic process | 6 | 3,60 | cellular amino acid metabolic process |
| 4 | cellular aldehyde metabolic process | 6 | 3,60 | cellular metabolic process |
| 7 | arginine metabolic process | 4 | 3,60 | glutamine family amino acid metabolic process |
| 8 | acetate derivative biosynthetic process | 6 | 3,60 | acetate derivative metabolic process, acetate biosynthetic process |
| 5 | DNA duplex unwinding | 6 | 3,60 | DNA geometric change |
| 8 | glycosaminoglycan biosynthetic process | 6 | 3,60 | aminoglycan biosynthetic process, glycosaminoglycan metabolic process |
| 6 | cellular polysaccharide metabolic process | 10 | 3,60 | polysaccharide metabolic process, cellular carbohydrate metabolic process, cellular macromolecule metabolic process |
| 4 | riboflavin metabolic process | 4 | 3,60 | riboflavin and derivative metabolic process, nitrogen compound metabolic process |
| 8 | branched chain family amino acid biosynthetic process | 2 | 3,60 | cellular amino acid biosynthetic process, branched chain family amino acid metabolic process |
| 5 | response to organic cyclic substance | 6 | 3,60 | response to organic substance |
| 5 | cellular amide metabolic process | 9 | 3,55 | cellular nitrogen compound metabolic process |
| 6 | glycoside metabolic process | 9 | 3,48 | cellular carbohydrate metabolic process |
| 2 | reproduction | 9 | 3,24 | biological_process |
| 6 | isoprenoid biosynthetic process | 4 | 3,20 | isoprenoid metabolic process, lipid biosynthetic process, cellular biosynthetic process |
| 2 | developmental process | 7 | 3,21 | biological_process |
| 5 | aromatic compound catabolic process | 4 | 3,22 | cellular catabolic process, cellular aromatic compound metabolic process |
| 6 | peptidyl-amino acid modification | 9 | 3,10 | protein modification process |
| 6 | streptomycin metabolic process | 5 | 3,00 | aminoglycoside antibiotic metabolic process |
| 9 | iron chelate transport | 3 | 3,00 | iron ion transport |
| 8 | molybdopterin cofactor biosynthetic process | 5 | 3,00 | pteridine and derivative biosynthetic process, molybdopterin cofactor metabolic process, cofactor biosynthetic process |
| 8 | regulation of translational elongation | 3 | 3,00 | regulation of translation, translational elongation |
| 9 | plasma membrane ATP synthesis coupled proton transport | 3 | 3,00 | ATP synthesis coupled proton transport |
| 6 | aminoglycoside antibiotic biosynthetic process | 5 | 3,00 | aminoglycoside antibiotic metabolic process, glycoside biosynthetic process, antibiotic biosynthetic process |
| 4 | response to starvation | 3 | 3,00 | response to stress, response to nutrient levels |
| 6 | protein amino acid methylation | 3 | 3,00 | protein amino acid alkylation, macromolecule methylation |
| 8 | mRNA catabolic process | 3 | 3,00 | mRNA metabolic process, RNA catabolic process |
| 8 | lysine catabolic process | 3 | 3,00 | aspartate family amino acid catabolic process, lysine metabolic process |
| 7 | threonine metabolic process | 3 | 3,00 | aspartate family amino acid metabolic process |
| 5 | methane metabolic process | 3 | 3,00 | cellular alkane metabolic process |
| 7 | L-serine metabolic process | 3 | 3,00 | serine family amino acid metabolic process |
| 6 | beta-lactam antibiotic catabolic process | 3 | 3,00 | antibiotic catabolic process, heterocycle catabolic process, cellular amide catabolic process, beta-lactam antibiotic metabolic process |
| 6 | lipopolysaccharide metabolic process | 5 | 3,00 | cellular lipid metabolic process, cellular polysaccharide metabolic process |
| 7 | negative regulation of flagellum assembly | 3 | 3,00 | regulation of flagellum assembly, negative regulation of cell projection organization, flagellum assembly |
| 6 | aldonic acid metabolic process | 5 | 3,00 | cellular carbohydrate metabolic process, monocarboxylic acid metabolic process |
| 7 | benzene and derivative metabolic process | 5 | 3,00 | cellular aromatic compound metabolic process, xenobiotic metabolic process |
| 8 | hexose biosynthetic process | 5 | 3,00 | hexose metabolic process, monosaccharide biosynthetic process |
| 6 | rRNA modification | 3 | 3,00 | RNA modification, rRNA processing |
| 10 | limonene catabolic process | 3 | 3,00 | monoterpene catabolic process, limonene metabolic process |
| 8 | Mo-molybdopterin cofactor metabolic process | 5 | 3,00 | molybdopterin cofactor metabolic process |
| 5 | glycolysis | 3 | 3,00 | generation of precursor metabolites and energy, glucose catabolic process |
| 8 | organophosphate ester transport | 3 | 3,00 | organic anion transport |
| 7 | positive regulation of nucleobase, nucleoside, nucleotide and nucleic acid metabolic process | 6 | 2,98 | positive regulation of nitrogen compound metabolic process, nucleobase, nucleoside, nucleotide and nucleic acid metabolic process, regulation of nucleobase, nucleoside, nucleotide and nucleic acid metabolic process, positive regulation of cellular metabolic process |
| 6 | indolalkylamine metabolic process | 6 | 2,98 | indole derivative metabolic process, cellular biogenic amine metabolic process |
| 5 | chemical homeostasis | 23 | 2,93 | homeostatic process |
| 9 | NADP metabolic process | 8 | 2,88 | nicotinamide nucleotide metabolic process |
| 6 | nucleobase catabolic process | 8 | 2,88 | heterocycle catabolic process, nucleobase, nucleoside and nucleotide catabolic process, nucleobase metabolic process |
| 8 | negative regulation by symbiont of host defense response | 2 | 2,88 | negative regulation by organism of defense response of other organism during symbiotic interaction, modulation by symbiont of host defense response, suppression of host defenses |
| 6 | xenobiotic metabolic process | 10 | 2,85 | cellular metabolic process, cellular response to xenobiotic stimulus |
| 5 | cellular nitrogen compound catabolic process | 14 | 2,78 | cellular catabolic process, cellular nitrogen compound metabolic process |
| 7 | positive regulation of gene expression | 5 | 2,76 | gene expression, regulation of gene expression, positive regulation of macromolecule metabolic process |
| 7 | positive regulation of macromolecule biosynthetic process | 5 | 2,76 | regulation of macromolecule biosynthetic process, macromolecule biosynthetic process, positive regulation of macromolecule metabolic process, positive regulation of biosynthetic process |
| 7 | positive regulation of cellular biosynthetic process | 5 | 2,76 | cellular biosynthetic process, regulation of cellular biosynthetic process, positive regulation of cellular metabolic process, positive regulation of biosynthetic process |
| 3 | organophosphate metabolic process | 7 | 2,72 | metabolic process |
| 6 | nucleoside metabolic process | 4 | 2,68 | nucleobase, nucleoside and nucleotide metabolic process |
| 5 | regulation of transport | 10 | 2,59 | regulation of localization, transport |
| 6 | regulation of establishment of protein localization | 10 | 2,59 | regulation of protein localization, establishment of protein localization |
| 5 | negative regulation of transport | 7 | 2,52 | negative regulation of biological process, transport, regulation of transport |
| 7 | tRNA aminoacylation | 5 | 2,52 | amino acid activation, tRNA metabolic process |
| 6 | glycosaminoglycan metabolic process | 7 | 2,52 | aminoglycan metabolic process |
| 6 | amide biosynthetic process | 7 | 2,52 | cellular nitrogen compound biosynthetic process, cellular amide metabolic process |
| 6 | ion transmembrane transport | 8 | 2,45 | transmembrane transport, ion transport |
| 4 | cell projection assembly | 6 | 2,45 | cellular component assembly, cell projection organization |
| 6 | cellular amide catabolic process | 4 | 2,40 | cellular nitrogen compound catabolic process, cellular amide metabolic process |
| 4 | vesicle organization | 4 | 2,40 | organelle organization |
| 6 | disaccharide metabolic process | 4 | 2,40 | cellular carbohydrate metabolic process, oligosaccharide metabolic process |
| 3 | taxis | 4 | 2,40 | locomotion, locomotory behavior, response to external stimulus |
| 7 | regulation of translation | 4 | 2,40 | regulation of cellular protein metabolic process, regulation of macromolecule biosynthetic process, posttranscriptional regulation of gene expression, translation, regulation of cellular biosynthetic process |
| 6 | cellular glucan metabolic process | 4 | 2,40 | glucan metabolic process, cellular polysaccharide metabolic process |
| 7 | lysine metabolic process | 5 | 2,38 | aspartate family amino acid metabolic process |
| 6 | monosaccharide catabolic process | 11 | 2,38 | alcohol catabolic process, cellular carbohydrate catabolic process, monosaccharide metabolic process |
| 6 | isoprenoid metabolic process | 7 | 2,31 | cellular lipid metabolic process |
| 3 | organelle organization | 7 | 2,29 | cellular component organization, cellular process |
| 4 | response to organic substance | 7 | 2,24 | response to chemical stimulus |
| 8 | pyridine nucleotide metabolic process | 9 | 2,24 | oxidoreduction coenzyme metabolic process, nucleotide metabolic process |
| 5 | defense response to bacterium | 3 | 2,20 | defense response, response to bacterium |
| 3 | DNA packaging | 3 | 2,20 | DNA conformation change, cellular component organization |
| 6 | aminoglycan biosynthetic process | 6 | 2,16 | aminoglycan metabolic process, polysaccharide biosynthetic process |
| 6 | riboflavin and derivative metabolic process | 4 | 2,16 | water-soluble vitamin metabolic process |
| 9 | acetate derivative metabolic process | 6 | 2,16 | acetate metabolic process |
| 7 | acetate biosynthetic process | 6 | 2,16 | acetate metabolic process, carboxylic acid biosynthetic process |
| 4 | cellular cell wall macromolecule metabolic process | 6 | 2,16 | cellular macromolecule metabolic process, cellular cell wall organization or biogenesis, cell wall macromolecule metabolic process |
| 4 | DNA geometric change | 6 | 2,16 | DNA conformation change |
| 2 | locomotion | 5 | 2,16 | biological_process |
| 6 | cellular component macromolecule biosynthetic process | 6 | 2,16 | cellular macromolecule biosynthetic process, cellular component biogenesis |
| 4 | innate immune response | 3 | 2,13 | immune response, defense response |
| 5 | flagellum assembly | 4 | 2,08 | flagellum organization, cell projection assembly |
| 3 | response to external stimulus | 7 | 2,09 | response to stimulus |
| 6 | oxidoreduction coenzyme metabolic process | 11 | 2,06 | coenzyme metabolic process |
| 6 | positive regulation of macromolecule metabolic process | 6 | 2,02 | positive regulation of metabolic process, regulation of macromolecule metabolic process, macromolecule metabolic process |
| 6 | tetrahydrobiopterin biosynthetic process | 2 | 2,00 | pteridine and derivative biosynthetic process, tetrahydrobiopterin metabolic process |
| 8 | valine biosynthetic process | 2 | 2,00 | branched chain family amino acid biosynthetic process, valine metabolic process |
| 8 | isoleucine biosynthetic process | 2 | 2,00 | branched chain family amino acid biosynthetic process, isoleucine metabolic process |
| 6 | cysteine metabolic process | 2 | 2,00 | sulfur amino acid metabolic process, serine family amino acid metabolic process |
| 5 | negative regulation of cell killing | 2 | 2,00 | regulation of cell killing, cell killing, negative regulation of biological process |
| 8 | galactose metabolic process | 2 | 2,00 | hexose metabolic process |
| 7 | beta-glucoside transport | 2 | 2,00 | glucoside transport |
| 5 | phospholipid catabolic process | 2 | 2,00 | phospholipid metabolic process, cellular lipid catabolic process |
| 7 | protein secretion by the type II secretion system | 2 | 2,00 | protein secretion |
| 5 | glutathione metabolic process | 2 | 2,00 | coenzyme metabolic process, cellular amino acid derivative metabolic process, sulfur metabolic process, peptide metabolic process |
| 8 | protein lipoylation | 2 | 2,00 | protein-cofactor linkage |
| 7 | protein secretion by the type IV secretion system | 2 | 2,00 | secretion by the type IV secretion system, protein secretion |
| 10 | negative regulation by symbiont of pathogen-associated molecular pattern-induced host innate immunity | 2 | 2,00 | negative regulation by symbiont of host innate immunity, negative regulation by organism of pathogen-associated molecular pattern-induced innate immunity of other organism during symbiotic interaction, modulation by symbiont of pathogen-associated molecular pattern-induced host innate immunity |
| 9 | atrazine catabolic process | 2 | 2,00 | atrazine metabolic process, s-triazine compound catabolic process |
| 5 | pilus assembly | 2 | 2,00 | pilus organization, cell projection assembly |
| 4 | response to cold | 2 | 2,00 | response to stress, response to temperature stimulus |
| 6 | cobalamin transport | 2 | 2,00 | vitamin transport |
| 6 | defense response to Gram-positive bacterium | 2 | 2,00 | defense response to bacterium |
| 9 | hexachlorocyclohexane metabolic process | 2 | 2,00 | chlorinated hydrocarbon metabolic process |
| 5 | D-ribose metabolic process | 2 | 2,00 | heterocycle metabolic process, pentose metabolic process |
| 4 | chromosome condensation | 2 | 2,00 | DNA packaging, chromosome organization |
| 6 | CDP-diacylglycerol metabolic process | 2 | 2,00 | diacylglycerol metabolic process, glycerophospholipid metabolic process |
| 6 | ubiquinone biosynthetic process | 2 | 2,00 | quinone cofactor biosynthetic process, ubiquinone metabolic process |
| 6 | steroid biosynthetic process | 2 | 2,00 | steroid metabolic process, lipid biosynthetic process |
| 8 | leucine biosynthetic process | 2 | 2,00 | branched chain family amino acid biosynthetic process, leucine metabolic process |
| 5 | ciliary or flagellar motility | 2 | 2,00 | cell motility |
| 7 | cytochrome complex assembly | 2 | 2,00 | cellular protein complex assembly |
| 6 | lipoprotein biosynthetic process | 2 | 2,00 | cellular macromolecule biosynthetic process, lipoprotein metabolic process |
| 6 | heme transport | 2 | 2,00 | cofactor transport |
| 7 | protein amino acid dephosphorylation | 2 | 2,00 | post-translational protein modification, dephosphorylation |
| 9 | dTMP biosynthetic process | 2 | 2,00 | pyrimidine deoxyribonucleoside monophosphate biosynthetic process, dTMP metabolic process |
| 8 | potassium ion transport | 2 | 2,00 | metal ion transport, monovalent inorganic cation transport |
| 5 | terpenoid biosynthetic process | 2 | 2,00 | isoprenoid biosynthetic process, terpenoid metabolic process |
| 6 | nucleobase transport | 2 | 2,00 | nucleobase, nucleoside, nucleotide and nucleic acid transport |
| 4 | binary fission | 2 | 2,00 | asexual reproduction, reproduction of a single-celled organism |
| 4 | barrier septum formation | 2 | 2,00 | cellular component assembly, cytokinetic process |
| 8 | phosphate transport | 2 | 2,00 | inorganic anion transport |
| 6 | amino acid transport | 3 | 1,96 | amine transport, carboxylic acid transport |
| 4 | lipid catabolic process | 6 | 1,95 | lipid metabolic process, catabolic process |
| 8 | chlorinated hydrocarbon metabolic process | 4 | 1,92 | halogenated hydrocarbon metabolic process |
| 5 | response to nutrient levels | 3 | 1,80 | response to extracellular stimulus |
| 8 | limonene metabolic process | 3 | 1,80 | monoterpene metabolic process |
| 8 | rRNA processing | 3 | 1,80 | rRNA metabolic process, ribosome biogenesis, ncRNA processing |
| 4 | cellular alkane metabolic process | 3 | 1,80 | cellular metabolic process |
| 5 | aminoglycoside antibiotic metabolic process | 5 | 1,80 | glycoside metabolic process, antibiotic metabolic process, amine metabolic process |
| 6 | glycoside biosynthetic process | 5 | 1,80 | glycoside metabolic process, cellular carbohydrate biosynthetic process |
| 6 | glucan metabolic process | 4 | 1,80 | polysaccharide metabolic process |
| 6 | negative regulation of cell projection organization | 3 | 1,80 | negative regulation of cellular process, regulation of cell projection organization, negative regulation of cellular component organization, cell projection organization |
| 8 | aspartate family amino acid catabolic process | 3 | 1,80 | aspartate family amino acid metabolic process, cellular amino acid catabolic process |
| 7 | molybdopterin cofactor metabolic process | 5 | 1,80 | prosthetic group metabolic process, pteridine and derivative metabolic process, coenzyme metabolic process |
| 6 | mRNA metabolic process | 3 | 1,80 | RNA metabolic process |
| 6 | quinone cofactor biosynthetic process | 3 | 1,80 | quinone cofactor metabolic process, coenzyme biosynthetic process |
| 6 | cellular protein complex assembly | 2 | 1,80 | cellular macromolecular complex assembly, protein complex assembly |
| 7 | translational elongation | 3 | 1,80 | cellular macromolecule biosynthetic process, translation |
| 6 | regulation of flagellum assembly | 3 | 1,80 | regulation of cell projection assembly, flagellum assembly |
| 8 | protein amino acid alkylation | 3 | 1,80 | post-translational protein modification |
| 8 | monoterpene catabolic process | 3 | 1,80 | terpene catabolic process, monoterpene metabolic process |
| 6 | monosaccharide biosynthetic process | 5 | 1,80 | alcohol biosynthetic process, cellular carbohydrate biosynthetic process, monosaccharide metabolic process |
| 6 | cellular biogenic amine metabolic process | 6 | 1,79 | cellular amine metabolic process, cellular amino acid derivative metabolic process |
| 5 | amine transport | 3 | 1,78 | transport |
| 6 | indole derivative metabolic process | 6 | 1,79 | indole and derivative metabolic process |
| 6 | positive regulation of cellular metabolic process | 6 | 1,79 | positive regulation of cellular process, cellular metabolic process, positive regulation of metabolic process, regulation of cellular metabolic process |
| 6 | positive regulation of nitrogen compound metabolic process | 6 | 1,79 | regulation of nitrogen compound metabolic process, positive regulation of metabolic process, nitrogen compound metabolic process |
| 7 | modulation by symbiont of host defense response | 2 | 1,77 | modification by symbiont of host morphology or physiology, response to host defenses, modulation by organism of defense response of other organism during symbiotic interaction |
| 6 | nucleobase, nucleoside and nucleotide catabolic process | 8 | 1,73 | nucleobase, nucleoside and nucleotide metabolic process, small molecule catabolic process, nucleobase, nucleoside, nucleotide and nucleic acid catabolic process |
| 7 | negative regulation by organism of defense response of other organism during symbiotic interaction | 2 | 1,73 | suppression of defenses of other organism during symbiotic interaction, negative regulation of defense response, modulation by organism of defense response of other organism during symbiotic interaction |
| 8 | nicotinamide nucleotide metabolic process | 8 | 1,73 | pyridine nucleotide metabolic process |
| 9 | suppression of host defenses | 2 | 1,73 | suppression of defenses of other organism during symbiotic interaction, avoidance of host defenses |
| 5 | cellular response to xenobiotic stimulus | 10 | 1,71 | cellular response to chemical stimulus, response to xenobiotic stimulus |
| 6 | glycerolipid metabolic process | 4 | 1,65 | cellular lipid metabolic process |
| 6 | positive regulation of biosynthetic process | 5 | 1,66 | positive regulation of metabolic process, regulation of biosynthetic process, biosynthetic process |
| 5 | alcohol catabolic process | 12 | 1,64 | small molecule catabolic process, alcohol metabolic process |
| 6 | lysine biosynthetic process via diaminopimelate | 2 | 1,60 | diaminopimelate metabolic process, lysine biosynthetic process |
| 3 | chromosome segregation | 2 | 1,60 | cellular process |
| 5 | polyol metabolic process | 2 | 1,60 | alcohol metabolic process |
| 8 | glutamine family amino acid biosynthetic process | 2 | 1,60 | glutamine family amino acid metabolic process, cellular amino acid biosynthetic process |
| 6 | L-phenylalanine metabolic process | 2 | 1,60 | aromatic amino acid family metabolic process |
| 6 | cellular lipid catabolic process | 5 | 1,59 | cellular catabolic process, cellular lipid metabolic process, lipid catabolic process |
| 5 | regulation of protein localization | 10 | 1,56 | regulation of localization, protein localization |
| 5 | glycerophospholipid metabolic process | 3 | 1,56 | phospholipid metabolic process, glycerolipid metabolic process |
| 6 | ncRNA metabolic process | 8 | 1,56 | RNA metabolic process |
| 4 | regulation of localization | 10 | 1,56 | localization, regulation of biological process |
| 6 | carboxylic acid transport | 4 | 1,54 | organic acid transport |
| 6 | aminoglycan metabolic process | 7 | 1,51 | polysaccharide metabolic process, amine metabolic process |
| 8 | tRNA metabolic process | 5 | 1,51 | ncRNA metabolic process |
| 6 | amino acid activation | 5 | 1,51 | cellular amino acid metabolic process |
| 3 | cell projection organization | 6 | 1,47 | cellular component organization, cellular process |
| 6 | modification by symbiont of host morphology or physiology | 2 | 1,47 | interaction with host, modification of morphology or physiology of other organism during symbiotic interaction |
| 4 | locomotory behavior | 4 | 1,44 | behavior |
| 5 | modification of morphology or physiology of other organism during symbiotic interaction | 2 | 1,44 | symbiosis, encompassing mutualism through parasitism, regulation of biological quality |
| 7 | posttranscriptional regulation of gene expression | 4 | 1,44 | regulation of gene expression |
| 6 | regulation of cellular protein metabolic process | 4 | 1,44 | cellular protein metabolic process, regulation of protein metabolic process, regulation of cellular metabolic process |
| 5 | oligosaccharide metabolic process | 4 | 1,44 | carbohydrate metabolic process |
| 6 | cellular carbohydrate catabolic process | 11 | 1,43 | cellular carbohydrate metabolic process, carbohydrate catabolic process |
| 6 | aromatic amino acid family biosynthetic process | 1 | 1,32 | aromatic amino acid family metabolic process, aromatic compound biosynthetic process, chorismate metabolic process, cellular amino acid biosynthetic process |
| 4 | response to bacterium | 3 | 1,32 | response to other organism |
| 7 | xenobiotic catabolic process | 3 | 1,32 | cellular catabolic process, xenobiotic metabolic process |
| 8 | acetate metabolic process | 6 | 1,30 | monocarboxylic acid metabolic process |
| 6 | pyrimidine nucleotide biosynthetic process | 3 | 1,25 | nucleotide biosynthetic process, pyrimidine nucleotide metabolic process |
| 7 | modulation by symbiont of host cellular process | 1 | 1,25 | modification by symbiont of host morphology or physiology, regulation of cellular process |
| 4 | flagellum organization | 4 | 1,25 | cell projection organization |
| 4 | cellular response to chemical stimulus | 12 | 1,23 | response to chemical stimulus, cellular response to stimulus |
| 8 | aromatic amino acid family biosynthetic process, prephenate pathway | 1 | 1,20 | aromatic amino acid family biosynthetic process |
| 5 | cofactor transport | 2 | 1,20 | transport |
| 9 | negative regulation by organism of pathogen-associated molecular pattern-induced innate immunity of other organism during symbiotic interaction | 2 | 1,20 | modulation by organism of pathogen-associated molecular pattern-induced innate immunity in other organism during symbiotic interaction, negative regulation by organism of innate immunity in other organism during symbiotic interaction |
| 8 | s-triazine compound catabolic process | 2 | 1,20 | xenobiotic catabolic process, heterocycle catabolic process, cellular nitrogen compound catabolic process, s-triazine compound metabolic process |
| 5 | cytokinetic process | 2 | 1,20 | cytokinesis, cellular process |
| 7 | dicarboxylic acid metabolic process | 3 | 1,20 | carboxylic acid metabolic process |
| 5 | lipoprotein metabolic process | 2 | 1,20 | cellular macromolecule metabolic process |
| 3 | asexual reproduction | 2 | 1,20 | reproduction |
| 5 | vitamin transport | 2 | 1,20 | transport |
| 5 | cellular macromolecular complex assembly | 3 | 1,21 | macromolecular complex assembly, cellular macromolecular complex subunit organization |
| 5 | steroid metabolic process | 2 | 1,20 | lipid metabolic process |
| 4 | peptide metabolic process | 2 | 1,20 | cellular metabolic process |
| 6 | pentose metabolic process | 2 | 1,20 | monosaccharide metabolic process |
| 8 | atrazine metabolic process | 2 | 1,20 | chlorinated hydrocarbon metabolic process, s-triazine compound metabolic process |
| 5 | positive regulation of metabolic process | 6 | 1,21 | regulation of metabolic process, metabolic process, positive regulation of biological process |
| 5 | nucleobase, nucleoside, nucleotide and nucleic acid transport | 2 | 1,20 | transport |
| 10 | modulation by symbiont of pathogen-associated molecular pattern-induced host innate immunity | 2 | 1,20 | modulation by organism of pathogen-associated molecular pattern-induced innate immunity in other organism during symbiotic interaction, modulation by symbiont of host innate immunity |
| 4 | regulation of cell killing | 2 | 1,20 | cell killing, regulation of biological process |
| 3 | reproduction of a single-celled organism | 2 | 1,20 | reproduction |
| 4 | carbohydrate catabolic process | 13 | 1,20 | carbohydrate metabolic process, catabolic process |
| 4 | terpenoid metabolic process | 2 | 1,20 | isoprenoid metabolic process, secondary metabolic process |
| 4 | pigment biosynthetic process | 2 | 1,20 | pigment metabolic process, biosynthetic process |
| 7 | secretion by the type IV secretion system | 2 | 1,20 | secretion by cell |
| 6 | dephosphorylation | 2 | 1,20 | phosphate metabolic process |
| 8 | dTMP metabolic process | 2 | 1,20 | pyrimidine deoxyribonucleoside monophosphate metabolic process |
| 9 | negative regulation by symbiont of host programmed cell death | 1 | 1,20 | modulation by symbiont of host programmed cell death, negative regulation by organism of programmed cell death in other organism during symbiotic interaction |
| 4 | pilus organization | 2 | 1,20 | cell projection organization |
| 8 | protein-cofactor linkage | 2 | 1,20 | post-translational protein modification |
| 5 | ubiquinone metabolic process | 2 | 1,20 | quinone cofactor metabolic process, cellular ketone metabolic process, oxidoreduction coenzyme metabolic process |
| 6 | tetrahydrobiopterin metabolic process | 2 | 1,20 | pteridine and derivative metabolic process |
| 9 | negative regulation by symbiont of host innate immunity | 2 | 1,20 | modulation by symbiont of host innate immunity, negative regulation by symbiont of host defense response, negative regulation by organism of innate immunity in other organism during symbiotic interaction, negative regulation by symbiont of host immune response |
| 5 | sulfur amino acid metabolic process | 2 | 1,20 | cellular amino acid metabolic process, sulfur metabolic process |
| 5 | diaminopimelate metabolic process | 2 | 1,20 | dicarboxylic acid metabolic process, amine metabolic process |
| 8 | diacylglycerol metabolic process | 2 | 1,20 | acylglycerol metabolic process |
| 4 | response to temperature stimulus | 2 | 1,20 | response to abiotic stimulus |
| 4 | chromosome organization | 2 | 1,20 | organelle organization |
| 8 | pyrimidine deoxyribonucleoside monophosphate biosynthetic process | 2 | 1,20 | pyrimidine deoxyribonucleotide biosynthetic process, deoxyribonucleoside monophosphate biosynthetic process, pyrimidine deoxyribonucleoside monophosphate metabolic process, pyrimidine nucleoside monophosphate biosynthetic process |
| 6 | glucoside transport | 2 | 1,20 | carbohydrate transport |
| 4 | cell motility | 2 | 1,20 | locomotion, cellular component movement, localization of cell |
| 7 | halogenated hydrocarbon metabolic process | 4 | 1,15 | xenobiotic metabolic process |
| 5 | apoptosis | 2 | 1,13 | programmed cell death |
| 3 | response to other organism | 5 | 1,11 | multi-organism process, response to biotic stimulus |
| 8 | monoterpene metabolic process | 3 | 1,08 | terpene metabolic process |
| 8 | terpene catabolic process | 3 | 1,08 | terpene metabolic process, isoprenoid catabolic process |
| 5 | positive regulation of cellular process | 6 | 1,07 | cellular process, regulation of cellular process, positive regulation of biological process |
| 5 | quinone cofactor metabolic process | 3 | 1,08 | cofactor metabolic process |
| 8 | ncRNA processing | 3 | 1,08 | RNA processing |
| 5 | regulation of cell projection assembly | 3 | 1,08 | cell projection assembly, regulation of cellular component biogenesis, regulation of cell projection organization |
| 5 | alcohol biosynthetic process | 5 | 1,08 | small molecule biosynthetic process, alcohol metabolic process |
| 7 | pyrimidine deoxyribonucleotide biosynthetic process | 3 | 1,08 | pyrimidine deoxyribonucleotide metabolic process, 2'-deoxyribonucleotide biosynthetic process, pyrimidine nucleotide biosynthetic process |
| 4 | response to extracellular stimulus | 3 | 1,08 | response to external stimulus |
| 6 | prosthetic group metabolic process | 5 | 1,08 | cellular protein metabolic process, cofactor metabolic process |
| 6 | modulation by organism of defense response of other organism during symbiotic interaction | 2 | 1,06 | response to defenses of other organism during symbiotic interaction, regulation of defense response, modification of morphology or physiology of other organism during symbiotic interaction |
| 7 | response to host defenses | 2 | 1,06 | response to defenses of other organism during symbiotic interaction, response to host |
| 4 | indole and derivative metabolic process | 6 | 1,07 | heterocycle metabolic process, nitrogen compound metabolic process, cellular aromatic compound metabolic process |
| 8 | rRNA metabolic process | 3 | 1,08 | ncRNA metabolic process |
| 5 | negative regulation of cellular component organization | 3 | 1,08 | regulation of cellular component organization, negative regulation of biological process, cellular component organization |
| 6 | negative regulation of defense response | 2 | 1,04 | negative regulation of response to stimulus, defense response, regulation of defense response |
| 5 | interaction with host | 2 | 1,03 | symbiosis, encompassing mutualism through parasitism, interspecies interaction between organisms |
| 7 | suppression of defenses of other organism during symbiotic interaction | 2 | 1,04 | avoidance of defenses of other organism during symbiotic interaction |
| 8 | avoidance of host defenses | 2 | 1,04 | avoidance of defenses of other organism during symbiotic interaction, response to host defenses |
| 5 | nucleobase, nucleoside, nucleotide and nucleic acid catabolic process | 8 | 1,04 | nucleobase, nucleoside, nucleotide and nucleic acid metabolic process, cellular nitrogen compound catabolic process |
| 4 | response to xenobiotic stimulus | 10 | 1,03 | response to chemical stimulus |
| 11 | positive regulation of RNA elongation from RNA polymerase II promoter | 1 | 1,00 | RNA elongation from RNA polymerase II promoter, regulation of RNA elongation from RNA polymerase II promoter, positive regulation of RNA elongation |
| 3 | microtubule-based process | 1 | 1,00 | cellular process |
| 7 | ribosomal large subunit assembly | 1 | 1,00 | ribosomal subunit assembly, ribosomal large subunit biogenesis |
| 8 | mismatch repair | 1 | 1,00 | DNA repair |
| 8 | protocatechuate catabolic process | 1 | 1,00 | catechol catabolic process, protocatechuate metabolic process |
| 4 | photosynthesis | 1 | 1,00 | cellular metabolic process |
| 8 | modulation by symbiont of host signal transduction pathway | 1 | 1,00 | modulation of signal transduction pathway in other organism during symbiotic interaction, modulation by symbiont of host cellular process |
| 5 | nucleotide-sugar metabolic process | 1 | 1,00 | nucleobase, nucleoside, nucleotide and nucleic acid metabolic process |
| 10 | negative regulation by symbiont of host apoptosis | 1 | 1,00 | modulation by symbiont of host apoptosis, negative regulation of apoptosis, negative regulation by symbiont of host programmed cell death |
| 8 | fructose metabolic process | 1 | 1,00 | hexose metabolic process |
| 8 | glycine catabolic process | 1 | 1,00 | glycine metabolic process, serine family amino acid catabolic process |
| 9 | glutamyl-tRNA aminoacylation | 1 | 1,00 | tRNA aminoacylation for protein translation |
| 7 | regulation of cell shape | 1 | 1,00 | regulation of cell morphogenesis, regulation of biological quality |
| 8 | arginine catabolic process | 1 | 1,00 | arginine metabolic process, glutamine family amino acid catabolic process |
| 7 | respiratory chain complex IV assembly | 1 | 1,00 | cellular protein complex assembly |
| 7 | protein secretion by the type I secretion system | 1 | 1,00 | protein secretion |
| 6 | corrin biosynthetic process | 1 | 1,00 | cofactor biosynthetic process, tetrapyrrole biosynthetic process, corrin metabolic process |
| 8 | tryptophan catabolic process to kynurenine | 1 | 1,00 | tryptophan catabolic process |
| 5 | regulation of chromosome segregation | 1 | 1,00 | chromosome segregation, regulation of cellular process |
| 6 | inositol metabolic process | 1 | 1,00 | cellular carbohydrate metabolic process, polyol metabolic process |
| 8 | lactose transport | 1 | 1,00 | disaccharide transport |
| 5 | riboflavin biosynthetic process | 1 | 1,00 | riboflavin metabolic process, cellular nitrogen compound biosynthetic process, riboflavin and derivative biosynthetic process, water-soluble vitamin biosynthetic process |
| 6 | lipid glycosylation | 1 | 1,00 | lipid modification |
| 5 | heme biosynthetic process | 1 | 1,00 | porphyrin biosynthetic process, pigment biosynthetic process, heme metabolic process |
| 8 | base-excision repair | 1 | 1,00 | DNA repair |
| 5 | peptidyl-diphthamide biosynthetic process from peptidyl-histidine | 1 | 1,00 | peptidyl-diphthamide metabolic process, cellular biosynthetic process |
| 6 | drug transmembrane transport | 1 | 1,00 | transmembrane transport, drug transport |
| 9 | SRP-dependent cotranslational protein targeting to membrane | 1 | 1,00 | cotranslational protein targeting to membrane, protein targeting to ER |
| 8 | L-glutamate transport | 1 | 1,00 | acidic amino acid transport, organic anion transport |
| 7 | pyrimidine ribonucleotide biosynthetic process | 1 | 1,00 | pyrimidine ribonucleotide metabolic process, pyrimidine nucleotide biosynthetic process, ribonucleotide biosynthetic process |
| 8 | interaction with host via protein secreted by type III secretion system | 1 | 1,00 | interaction with host via secreted substance during symbiotic interaction, interaction with other organism via protein secreted by type III secretion system during symbiotic interaction |
| 3 | cell cycle | 1 | 1,00 | cellular process |
| 6 | polyamine transport | 1 | 1,00 | amine transport |
| 7 | thiamin biosynthetic process | 1 | 1,00 | thiamin and derivative biosynthetic process, thiamin metabolic process |
| 5 | nicotinamide metabolic process | 1 | 1,00 | alkaloid metabolic process, pyridine nucleotide metabolic process, cellular amide metabolic process |
| 6 | hydrogen peroxide catabolic process | 1 | 1,00 | cellular response to hydrogen peroxide, cellular catabolic process, hydrogen peroxide metabolic process |
| 7 | killing of cells in other organism during symbiotic interaction | 1 | 1,00 | killing of cells of another organism, disruption of cells of other organism during symbiotic interaction |
| 6 | sporulation resulting in formation of a cellular spore | 1 | 1,00 | anatomical structure formation involved in morphogenesis, sporulation, cell differentiation |
| 10 | dUTP biosynthetic process | 1 | 1,00 | dUTP metabolic process, pyrimidine deoxyribonucleoside triphosphate biosynthetic process |
| 6 | pyrroloquinoline quinone biosynthetic process | 1 | 1,00 | heterocycle biosynthetic process, quinone cofactor biosynthetic process, cellular nitrogen compound biosynthetic process, peptidyl-tyrosine modification, carboxylic acid biosynthetic process |
| 7 | ethanol oxidation | 1 | 1,00 | ethanol metabolic process |
| 8 | protein-chromophore linkage | 1 | 1,00 | post-translational protein modification |
| 6 | nucleoside biosynthetic process | 1 | 1,00 | nucleoside metabolic process, nucleobase, nucleoside and nucleotide biosynthetic process |
| 10 | negative regulation by symbiont of defense-related host cell wall callose deposition | 1 | 1,00 | negative regulation by symbiont of defense-related host callose deposition, negative regulation by organism of defense-related cell wall callose deposition in other organism during symbiotic interaction, modulation by symbiont of defense-related host cell wall callose deposition |
| 8 | regulation of translational initiation | 1 | 1,00 | translational initiation, regulation of translation |
| 8 | glutamine biosynthetic process | 1 | 1,00 | glutamine family amino acid biosynthetic process, glutamine metabolic process |
| 10 | 'de novo' IMP biosynthetic process | 1 | 1,00 | IMP biosynthetic process |
| 9 | negative regulation by symbiont of host salicylic acid-mediated defense response | 1 | 1,00 | negative regulation by organism of salicylic acid-mediated defense response of other organism during symbiotic interaction, systemic acquired resistance, salicylic acid mediated signaling pathway, modulation by symbiont of host salicylic acid-mediated defense response, negative regulation by symbiont of host defense response, negative regulation of signal transduction |
| 9 | reductive tricarboxylic acid cycle | 1 | 1,00 | carbon fixation, tricarboxylic acid cycle |
| 9 | positive regulation by symbiont of host jasmonic acid-mediated defense response | 1 | 1,00 | positive regulation by symbiont of host immune response, positive regulation by organism of jasmonic acid-mediated defense response of other organism during symbiotic interaction, positive regulation by symbiont of host defense response, modulation by symbiont of host jasmonic acid-mediated defense response |
| null | glutathione conjugation reaction | 1 | 1,00 | obsolete_biological_process |
| 8 | tryptophan biosynthetic process | 1 | 1,00 | tryptophan metabolic process, indolalkylamine biosynthetic process, aromatic amino acid family biosynthetic process |
| 7 | branched-chain aliphatic amino acid transport | 1 | 1,00 | amino acid transport |
| 8 | caprolactam catabolic process | 1 | 1,00 | xenobiotic catabolic process, caprolactam metabolic process, heterocycle catabolic process, cellular amide catabolic process |
| 8 | cellulose catabolic process | 1 | 1,00 | cellulose metabolic process, glucan catabolic process |
| 8 | cellular iron ion homeostasis | 1 | 1,00 | cellular di-, tri-valent inorganic cation homeostasis, iron ion homeostasis |
| 8 | positive regulation of transcription, DNA-dependent | 1 | 1,00 | regulation of transcription, DNA-dependent, transcription, DNA-dependent, positive regulation of RNA metabolic process, positive regulation of transcription |
| 8 | mannose biosynthetic process | 1 | 1,00 | hexose biosynthetic process, mannose metabolic process |
| 5 | chlorophyll biosynthetic process | 1 | 1,00 | porphyrin biosynthetic process, pigment biosynthetic process, chlorophyll metabolic process |
| 8 | tyrosine biosynthetic process | 1 | 1,00 | aromatic amino acid family biosynthetic process, prephenate pathway, tyrosine metabolic process |
| 9 | alanyl-tRNA aminoacylation | 1 | 1,00 | tRNA aminoacylation for protein translation |
| 8 | extracellular polysaccharide biosynthetic process | 1 | 1,00 | cellular polysaccharide biosynthetic process, extracellular polysaccharide metabolic process |
| 9 | negative regulation by symbiont of host defense-related protein level | 1 | 1,00 | modulation by symbiont of host defense-related protein level, negative regulation by symbiont of host defense response |
| 8 | peptidoglycan catabolic process | 1 | 1,00 | glycosaminoglycan catabolic process, peptidoglycan metabolic process |
| 8 | phosphoinositide biosynthetic process | 1 | 1,00 | phosphoinositide metabolic process, glycerophospholipid biosynthetic process |
| 8 | L-phenylalanine biosynthetic process | 1 | 1,00 | aromatic amino acid family biosynthetic process, prephenate pathway, L-phenylalanine metabolic process |
| 8 | bile acid and bile salt transport | 1 | 1,00 | monocarboxylic acid transport |
| 9 | mitochondrial electron transport, cytochrome c to oxygen | 1 | 1,00 | mitochondrial ATP synthesis coupled electron transport, respiratory electron transport chain |
| 8 | nicotinate nucleotide metabolic process | 1 | 1,00 | pyridine nucleotide metabolic process |
| 7 | diaminopimelate biosynthetic process | 1 | 1,00 | diaminopimelate metabolic process, carboxylic acid biosynthetic process, lysine biosynthetic process via diaminopimelate |
| 4 | bioluminescence | 1 | 1,00 | cellular metabolic process |
| 7 | protein tetramerization | 1 | 1,00 | protein oligomerization |
| 9 | negative regulation by symbiont of host defense-related programmed cell death | 1 | 1,00 | negative regulation by symbiont of host programmed cell death, negative regulation by symbiont of host defense response, modulation by symbiont of host defense-related programmed cell death |
| 5 | peptide transport | 1 | 1,00 | transport |
| 6 | phytochromobilin biosynthetic process | 1 | 1,00 | cofactor biosynthetic process, tetrapyrrole biosynthetic process, phytochromobilin metabolic process |
| 8 | lysine biosynthetic process | 2 | 0,96 | aspartate family amino acid biosynthetic process, lysine metabolic process |
| 7 | positive regulation of RNA metabolic process | 2 | 0,96 | RNA metabolic process, regulation of RNA metabolic process, positive regulation of macromolecule metabolic process, positive regulation of nucleobase, nucleoside, nucleotide and nucleic acid metabolic process |
| 4 | symbiosis, encompassing mutualism through parasitism | 2 | 0,96 | interspecies interaction between organisms |
| 5 | organic acid transport | 4 | 0,92 | transport |
| 3 | behavior | 4 | 0,86 | response to stimulus |
| 6 | regulation of protein metabolic process | 4 | 0,86 | regulation of primary metabolic process, regulation of macromolecule metabolic process, protein metabolic process |
| 4 | positive regulation of biological process | 7 | 0,80 | regulation of biological process, biological_process |
| 8 | modulation by symbiont of host immune response | 2 | 0,79 | response to host immune response, modulation by symbiont of host defense response, modulation by organism of immune response of other organism during symbiotic interaction |
| 8 | chorismate metabolic process | 1 | 0,79 | dicarboxylic acid metabolic process |
| 4 | programmed cell death | 2 | 0,76 | cell death |
| 4 | regulation of cellular component organization | 4 | 0,75 | regulation of biological process, cellular component organization |
| 5 | pyrimidine nucleotide metabolic process | 3 | 0,75 | heterocycle metabolic process, nucleotide metabolic process |
| 9 | modulation by symbiont of host innate immunity | 2 | 0,72 | modulation by symbiont of host immune response, modulation by organism of innate immunity in other organism during symbiotic interaction |
| 3 | response to abiotic stimulus | 2 | 0,72 | response to stimulus |
| 3 | pigment metabolic process | 2 | 0,72 | metabolic process |
| 8 | deoxyribonucleoside monophosphate biosynthetic process | 2 | 0,72 | deoxyribonucleoside monophosphate metabolic process, deoxyribonucleotide biosynthetic process, nucleoside monophosphate biosynthetic process |
| 3 | cellular component movement | 2 | 0,72 | cellular process |
| 8 | negative regulation by organism of innate immunity in other organism during symbiotic interaction | 2 | 0,72 | negative regulation by organism of defense response of other organism during symbiotic interaction, negative regulation by organism of immune response of other organism during symbiotic interaction, modulation by organism of innate immunity in other organism during symbiotic interaction |
| 7 | s-triazine compound metabolic process | 2 | 0,72 | heterocycle metabolic process, nitrogen compound metabolic process, cellular aromatic compound metabolic process, xenobiotic metabolic process |
| 9 | negative regulation by symbiont of host immune response | 2 | 0,72 | modulation by symbiont of host immune response, negative regulation by organism of immune response of other organism during symbiotic interaction |
| 7 | pyrimidine nucleoside monophosphate biosynthetic process | 2 | 0,72 | pyrimidine nucleoside monophosphate metabolic process, pyrimidine nucleotide biosynthetic process, nucleoside monophosphate biosynthetic process |
| 3 | cellular macromolecular complex subunit organization | 3 | 0,73 | macromolecular complex subunit organization, cellular process |
| 5 | regulation of signal transduction | 2 | 0,72 | signal transduction, regulation of cell communication, regulation of signaling process |
| 3 | localization of cell | 2 | 0,72 | localization |
| 9 | modulation by organism of pathogen-associated molecular pattern-induced innate immunity in other organism during symbiotic interaction | 2 | 0,72 | modulation by organism of innate immunity in other organism during symbiotic interaction |
| 4 | cytokinesis | 2 | 0,72 | cell division |
| 8 | negative regulation by organism of programmed cell death in other organism during symbiotic interaction | 1 | 0,72 | negative regulation of programmed cell death, modulation of programmed cell death in other organism during symbiotic interaction |
| 8 | modulation by symbiont of host programmed cell death | 1 | 0,72 | modulation by symbiont of host cellular process, modulation of programmed cell death in other organism during symbiotic interaction |
| 7 | pyrimidine deoxyribonucleoside monophosphate metabolic process | 2 | 0,72 | deoxyribonucleoside monophosphate metabolic process, pyrimidine deoxyribonucleotide metabolic process, pyrimidine nucleoside monophosphate metabolic process |
| 6 | acylglycerol metabolic process | 2 | 0,72 | neutral lipid metabolic process, glycerolipid metabolic process, glycerol ether metabolic process |
| 3 | response to biotic stimulus | 5 | 0,67 | response to stimulus |
| 5 | regulation of cell projection organization | 3 | 0,65 | regulation of cellular component organization, cell projection organization, regulation of cellular process |
| 6 | terpene metabolic process | 3 | 0,65 | isoprenoid metabolic process |
| 6 | pyrimidine deoxyribonucleotide metabolic process | 3 | 0,65 | 2'-deoxyribonucleotide metabolic process, pyrimidine nucleotide metabolic process |
| 8 | 2'-deoxyribonucleotide biosynthetic process | 3 | 0,65 | 2'-deoxyribonucleotide metabolic process, deoxyribonucleotide biosynthetic process |
| 4 | regulation of cellular component biogenesis | 3 | 0,65 | cellular component biogenesis, regulation of biological process |
| 6 | RNA processing | 3 | 0,65 | RNA metabolic process, gene expression |
| 6 | isoprenoid catabolic process | 3 | 0,65 | isoprenoid metabolic process, cellular lipid catabolic process |
| 6 | response to host | 2 | 0,64 | interaction with host, response to other organism |
| 6 | regulation of defense response | 2 | 0,64 | regulation of response to stress, defense response |
| 6 | avoidance of defenses of other organism during symbiotic interaction | 2 | 0,62 | response to defenses of other organism during symbiotic interaction |
| 5 | negative regulation of response to stimulus | 2 | 0,62 | response to stimulus, regulation of response to stimulus, negative regulation of biological process |
| 5 | corrin metabolic process | 1 | 0,60 | tetrapyrrole metabolic process, cofactor metabolic process |
| 8 | mannose metabolic process | 1 | 0,60 | hexose metabolic process |
| 7 | caprolactam metabolic process | 1 | 0,60 | heterocycle metabolic process, cellular amide metabolic process, xenobiotic metabolic process |
| 8 | serine family amino acid catabolic process | 1 | 0,60 | serine family amino acid metabolic process, cellular amino acid catabolic process |
| 7 | cellular di-, tri-valent inorganic cation homeostasis | 1 | 0,60 | di-, tri-valent inorganic cation homeostasis, cellular cation homeostasis |
| 6 | phosphoinositide metabolic process | 1 | 0,60 | glycerophospholipid metabolic process |
| 8 | positive regulation of RNA elongation | 1 | 0,60 | regulation of RNA elongation, positive regulation of RNA metabolic process, RNA elongation |
| 6 | glycerophospholipid biosynthetic process | 1 | 0,60 | glycerolipid biosynthetic process, glycerophospholipid metabolic process, phospholipid biosynthetic process |
| 8 | modulation by symbiont of host defense-related programmed cell death | 1 | 0,60 | modulation by symbiont of host defense response, modulation by symbiont of host programmed cell death |
| 7 | interaction with host via secreted substance during symbiotic interaction | 1 | 0,60 | interaction with host via substance released outside of symbiont, interaction with other organism via secreted substance during symbiotic interaction |
| 8 | glutamine family amino acid catabolic process | 1 | 0,60 | glutamine family amino acid metabolic process, cellular amino acid catabolic process |
| 7 | protocatechuate metabolic process | 1 | 0,60 | monocarboxylic acid metabolic process, catechol metabolic process |
| 7 | cellular response to hydrogen peroxide | 1 | 0,60 | response to hydrogen peroxide, cellular response to reactive oxygen species |
| 9 | IMP biosynthetic process | 1 | 0,60 | purine ribonucleoside monophosphate biosynthetic process, IMP metabolic process |
| 4 | heme metabolic process | 1 | 0,60 | pigment metabolic process, porphyrin metabolic process |
| 6 | protein oligomerization | 1 | 0,60 | protein complex assembly |
| 6 | thiamin and derivative biosynthetic process | 1 | 0,60 | thiamin and derivative metabolic process, aromatic compound biosynthetic process, heterocycle biosynthetic process, cellular nitrogen compound biosynthetic process, sulfur compound biosynthetic process, water-soluble vitamin biosynthetic process |
| 7 | translational initiation | 1 | 0,60 | translation, cellular process |
| 6 | glucan catabolic process | 1 | 0,60 | glucan metabolic process, polysaccharide catabolic process |
| 9 | negative regulation by symbiont of defense-related host callose deposition | 1 | 0,60 | modulation by symbiont of defense-related host callose deposition, negative regulation by symbiont of host defense response, negative regulation by organism of defense-related callose deposition in other organism during symbiotic interaction |
| 8 | positive regulation by symbiont of host defense response | 1 | 0,60 | modulation by symbiont of host defense response, positive regulation by organism of defense response of other organism during symbiotic interaction |
| 10 | regulation of RNA elongation from RNA polymerase II promoter | 1 | 0,60 | regulation of RNA elongation, RNA elongation from RNA polymerase II promoter, regulation of transcription from RNA polymerase II promoter |
| 7 | catechol catabolic process | 1 | 0,60 | diol catabolic process, catechol metabolic process |
| 4 | carbon fixation | 1 | 0,60 | organic substance metabolic process |
| 7 | acidic amino acid transport | 1 | 0,60 | amino acid transport |
| 8 | glycosaminoglycan catabolic process | 1 | 0,60 | glycosaminoglycan metabolic process, aminoglycan catabolic process |
| 5 | ribosomal large subunit biogenesis | 1 | 0,60 | ribosome biogenesis, ribonucleoprotein complex biogenesis |
| 7 | disaccharide transport | 1 | 0,60 | oligosaccharide transport |
| 9 | cotranslational protein targeting to membrane | 1 | 0,60 | protein targeting to membrane |
| 9 | negative regulation by organism of defense-related cell wall callose deposition in other organism during symbiotic interaction | 1 | 0,60 | modulation by organism of defense-related cell wall callose deposition in other organism during symbiotic interaction, negative regulation by organism of defense-related callose deposition in other organism during symbiotic interaction |
| 9 | modulation by symbiont of host jasmonic acid-mediated defense response | 1 | 0,60 | modulation by symbiont of host immune response, modulation by organism of jasmonic acid-mediated defense response of other organism during symbiotic interaction |
| 6 | extracellular polysaccharide metabolic process | 1 | 0,60 | cellular polysaccharide metabolic process |
| 8 | peptidyl-diphthamide metabolic process | 1 | 0,60 | peptidyl-histidine modification |
| 9 | modulation by symbiont of defense-related host cell wall callose deposition | 1 | 0,60 | modulation by organism of defense-related cell wall callose deposition in other organism during symbiotic interaction, modulation by symbiont of defense-related host callose deposition, modulation by symbiont of defense-related host cell wall thickening |
| 9 | RNA elongation from RNA polymerase II promoter | 1 | 0,60 | transcription from RNA polymerase II promoter, RNA elongation |
| 3 | sporulation | 1 | 0,60 | developmental process |
| 9 | positive regulation by symbiont of host immune response | 1 | 0,60 | modulation by symbiont of host immune response, positive regulation by organism of immune response of other organism during symbiotic interaction |
| 9 | modulation by symbiont of host apoptosis | 1 | 0,60 | modulation by organism of apoptosis in other organism during symbiotic interaction, modulation by symbiont of host programmed cell death |
| 8 | tryptophan catabolic process | 1 | 0,60 | tryptophan metabolic process, aromatic amino acid family catabolic process, indolalkylamine catabolic process |
| 6 | pyrimidine ribonucleotide metabolic process | 1 | 0,60 | ribonucleotide metabolic process, pyrimidine nucleotide metabolic process |
| 6 | interaction with other organism via protein secreted by type III secretion system during symbiotic interaction | 1 | 0,60 | interaction with other organism via secreted substance during symbiotic interaction |
| 4 | anatomical structure formation involved in morphogenesis | 1 | 0,60 | anatomical structure morphogenesis, developmental process |
| 8 | dUTP metabolic process | 1 | 0,60 | pyrimidine deoxyribonucleoside triphosphate metabolic process |
| 4 | alkaloid metabolic process | 1 | 0,60 | nitrogen compound metabolic process, secondary metabolic process |
| 8 | modulation by symbiont of host defense-related protein level | 1 | 0,60 | modulation by symbiont of host defense response, modulation by symbiont of host protein levels |
| 8 | systemic acquired resistance, salicylic acid mediated signaling pathway | 1 | 0,60 | systemic acquired resistance, salicylic acid mediated signaling pathway |
| 9 | modulation by symbiont of host salicylic acid-mediated defense response | 1 | 0,60 | modulation by symbiont of host defense response, modulation by organism of salicylic acid-mediated defense response of other organism during symbiotic interaction, regulation of signal transduction, regulation of systemic acquired resistance, systemic acquired resistance, salicylic acid mediated signaling pathway |
| 6 | indolalkylamine biosynthetic process | 1 | 0,60 | indolalkylamine metabolic process, heterocycle biosynthetic process, cellular biogenic amine biosynthetic process, indole derivative biosynthetic process |
| 6 | negative regulation of signal transduction | 1 | 0,60 | negative regulation of signaling process, signal transduction, regulation of signal transduction, negative regulation of cell communication |
| 6 | lipid modification | 1 | 0,60 | cellular lipid metabolic process |
| 5 | hydrogen peroxide metabolic process | 1 | 0,60 | oxygen and reactive oxygen species metabolic process |
| 9 | mitochondrial ATP synthesis coupled electron transport | 1 | 0,60 | ATP synthesis coupled electron transport |
| 8 | peptidyl-tyrosine modification | 1 | 0,60 | peptidyl-amino acid modification |
| 8 | negative regulation of apoptosis | 1 | 0,60 | negative regulation of programmed cell death, regulation of apoptosis, apoptosis |
| 5 | phytochromobilin metabolic process | 1 | 0,60 | tetrapyrrole metabolic process, cofactor metabolic process |
| 6 | ribosomal subunit assembly | 1 | 0,60 | ribosome assembly |
| 4 | cell differentiation | 1 | 0,60 | cellular developmental process |
| 6 | thiamin metabolic process | 1 | 0,60 | thiamin and derivative metabolic process |
| 7 | riboflavin and derivative biosynthetic process | 1 | 0,60 | riboflavin and derivative metabolic process |
| 8 | pyrimidine deoxyribonucleoside triphosphate biosynthetic process | 1 | 0,60 | pyrimidine nucleoside triphosphate biosynthetic process, pyrimidine deoxyribonucleotide biosynthetic process, pyrimidine deoxyribonucleoside triphosphate metabolic process, deoxyribonucleoside triphosphate biosynthetic process |
| 7 | monocarboxylic acid transport | 1 | 0,60 | carboxylic acid transport |
| 8 | negative regulation by organism of salicylic acid-mediated defense response of other organism during symbiotic interaction | 1 | 0,60 | negative regulation by organism of defense response of other organism during symbiotic interaction, modulation by organism of salicylic acid-mediated defense response of other organism during symbiotic interaction |
| 6 | disruption of cells of other organism during symbiotic interaction | 1 | 0,60 | modification of morphology or physiology of other organism during symbiotic interaction |
| 6 | cellulose metabolic process | 1 | 0,60 | glucan metabolic process |
| 8 | positive regulation by organism of jasmonic acid-mediated defense response of other organism during symbiotic interaction | 1 | 0,60 | positive regulation by organism of defense response of other organism during symbiotic interaction, modulation by organism of jasmonic acid-mediated defense response of other organism during symbiotic interaction |
| 6 | modulation of signal transduction pathway in other organism during symbiotic interaction | 1 | 0,60 | regulation of signal transduction, modification of morphology or physiology of other organism during symbiotic interaction |
| 8 | protein targeting to ER | 1 | 0,60 | protein targeting, protein localization in endoplasmic reticulum |
| 6 | ethanol metabolic process | 1 | 0,60 | monohydric alcohol metabolic process |
| 9 | iron ion homeostasis | 1 | 0,60 | di-, tri-valent inorganic cation homeostasis |
| 8 | tricarboxylic acid cycle | 1 | 0,60 | acetyl-CoA catabolic process, aerobic respiration |
| 5 | regulation of cell morphogenesis | 1 | 0,60 | cell morphogenesis, regulation of cellular component organization, regulation of cellular process, regulation of anatomical structure morphogenesis |
| 6 | polysaccharide catabolic process | 2 | 0,58 | polysaccharide metabolic process, macromolecule catabolic process, carbohydrate catabolic process |
| 8 | aspartate family amino acid biosynthetic process | 2 | 0,58 | aspartate family amino acid metabolic process, cellular amino acid biosynthetic process |
| 3 | interspecies interaction between organisms | 2 | 0,58 | multi-organism process |
| 7 | negative regulation of programmed cell death | 1 | 0,58 | regulation of programmed cell death, programmed cell death, negative regulation of cell death |
| 8 | nucleoside monophosphate biosynthetic process | 3 | 0,56 | nucleotide biosynthetic process, nucleoside monophosphate metabolic process |
| 5 | response to defenses of other organism during symbiotic interaction | 2 | 0,53 | symbiosis, encompassing mutualism through parasitism, response to other organism |
| 4 | anatomical structure morphogenesis | 2 | 0,49 | anatomical structure development, developmental process |
| 3 | cellular developmental process | 2 | 0,49 | developmental process, cellular process |
| 7 | modulation by organism of immune response of other organism during symbiotic interaction | 2 | 0,48 | regulation of immune response, response to immune response of other organism during symbiotic interaction, modulation by organism of defense response of other organism during symbiotic interaction |
| 8 | response to host immune response | 2 | 0,48 | response to host defenses, response to immune response of other organism during symbiotic interaction |
| 8 | deoxyribonucleoside monophosphate metabolic process | 2 | 0,43 | deoxyribonucleotide metabolic process, nucleoside monophosphate metabolic process |
| 5 | glycerol ether metabolic process | 2 | 0,43 | organic ether metabolic process |
| 6 | modulation of programmed cell death in other organism during symbiotic interaction | 1 | 0,43 | regulation of programmed cell death, modification of morphology or physiology of other organism during symbiotic interaction |
| 8 | negative regulation by organism of immune response of other organism during symbiotic interaction | 2 | 0,43 | negative regulation of immune response, modulation by organism of immune response of other organism during symbiotic interaction |
| 6 | neutral lipid metabolic process | 2 | 0,43 | cellular lipid metabolic process |
| 5 | regulation of cell communication | 2 | 0,43 | regulation of cellular process, cell communication |
| 4 | regulation of signaling process | 2 | 0,43 | signaling process, regulation of biological process |
| 6 | pyrimidine nucleoside monophosphate metabolic process | 2 | 0,43 | pyrimidine nucleotide metabolic process, nucleoside monophosphate metabolic process |
| 8 | modulation by organism of innate immunity in other organism during symbiotic interaction | 2 | 0,43 | modulation by organism of immune response of other organism during symbiotic interaction |
| 5 | regulation of immune response | 2 | 0,41 | regulation of immune system process, regulation of response to stimulus, immune response |
| 8 | 2'-deoxyribonucleotide metabolic process | 3 | 0,39 | deoxyribonucleotide metabolic process |
| 8 | deoxyribonucleotide biosynthetic process | 3 | 0,39 | deoxyribonucleotide metabolic process, nucleotide biosynthetic process |
| 5 | regulation of response to stress | 2 | 0,38 | response to stress, regulation of response to stimulus |
| 6 | cellular response to reactive oxygen species | 1 | 0,36 | cellular response to oxidative stress, response to reactive oxygen species |
| 8 | protein targeting to membrane | 1 | 0,36 | protein targeting |
| 7 | modulation by organism of salicylic acid-mediated defense response of other organism during symbiotic interaction | 1 | 0,36 | modulation by organism of defense response of other organism during symbiotic interaction |
| 8 | modulation by symbiont of defense-related host cell wall thickening | 1 | 0,36 | modulation by symbiont of host defense response, modulation by symbiont of host cellular process, modulation by organism of defense-related cell wall thickening in other organism during symbiotic interaction |
| 8 | positive regulation by organism of immune response of other organism during symbiotic interaction | 1 | 0,36 | positive regulation of immune response, modulation by organism of immune response of other organism during symbiotic interaction |
| 7 | regulation of systemic acquired resistance | 1 | 0,36 | systemic acquired resistance, regulation of innate immune response, regulation of response to biotic stimulus, regulation of multi-organism process |
| 9 | regulation of transcription from RNA polymerase II promoter | 1 | 0,36 | regulation of transcription, DNA-dependent, transcription from RNA polymerase II promoter |
| 6 | systemic acquired resistance | 1 | 0,36 | defense response, incompatible interaction |
| 6 | oligosaccharide transport | 1 | 0,36 | carbohydrate transport |
| 5 | negative regulation of signaling process | 1 | 0,36 | regulation of signaling process |
| 6 | cellular cation homeostasis | 1 | 0,36 | cation homeostasis, cellular ion homeostasis |
| 6 | response to hydrogen peroxide | 1 | 0,36 | response to reactive oxygen species |
| 6 | ATP synthesis coupled electron transport | 1 | 0,36 | respiratory electron transport chain, oxidative phosphorylation |
| 5 | ribosome assembly | 1 | 0,36 | organelle assembly, ribosome biogenesis, ribonucleoprotein complex assembly |
| 8 | modulation by symbiont of defense-related host callose deposition | 1 | 0,36 | modulation by organism of defense-related callose deposition of other organism during symbiotic interaction, modulation by symbiont of host defense response |
| 6 | glycerolipid biosynthetic process | 1 | 0,36 | lipid biosynthetic process, glycerolipid metabolic process, cellular biosynthetic process |
| 6 | cellular biogenic amine biosynthetic process | 1 | 0,36 | amine biosynthetic process, cellular amino acid derivative biosynthetic process, cellular biogenic amine metabolic process |
| 8 | deoxyribonucleoside triphosphate biosynthetic process | 1 | 0,36 | deoxyribonucleoside triphosphate metabolic process, nucleoside triphosphate biosynthetic process, deoxyribonucleotide biosynthetic process |
| 6 | indolalkylamine catabolic process | 1 | 0,36 | indolalkylamine metabolic process, heterocycle catabolic process, indole derivative catabolic process, cellular biogenic amine catabolic process |
| 8 | modulation by organism of defense-related cell wall callose deposition in other organism during symbiotic interaction | 1 | 0,36 | modulation by organism of defense-related callose deposition of other organism during symbiotic interaction, modulation by organism of defense-related cell wall thickening in other organism during symbiotic interaction |
| 5 | interaction with other organism via secreted substance during symbiotic interaction | 1 | 0,36 | symbiosis, encompassing mutualism through parasitism, interspecies interaction between organisms |
| 6 | negative regulation of cell communication | 1 | 0,36 | negative regulation of cellular process, regulation of cell communication, cell communication |
| 7 | protein localization in endoplasmic reticulum | 1 | 0,36 | protein localization in organelle |
| 7 | pyrimidine nucleoside triphosphate biosynthetic process | 1 | 0,36 | pyrimidine nucleoside triphosphate metabolic process, nucleoside triphosphate biosynthetic process, pyrimidine nucleotide biosynthetic process |
| 8 | negative regulation by organism of defense-related callose deposition in other organism during symbiotic interaction | 1 | 0,36 | negative regulation by organism of defense response of other organism during symbiotic interaction, modulation by organism of defense-related callose deposition of other organism during symbiotic interaction |
| 6 | catechol metabolic process | 1 | 0,36 | phenol metabolic process, diol metabolic process |
| 8 | regulation of RNA elongation | 1 | 0,36 | regulation of transcription, DNA-dependent, RNA elongation |
| 7 | acetyl-CoA catabolic process | 1 | 0,36 | coenzyme catabolic process, acetyl-CoA metabolic process |
| 6 | interaction with host via substance released outside of symbiont | 1 | 0,36 | interaction with host |
| 4 | thiamin and derivative metabolic process | 1 | 0,36 | heterocycle metabolic process, water-soluble vitamin metabolic process, nitrogen compound metabolic process, cellular aromatic compound metabolic process, sulfur metabolic process |
| 6 | cell morphogenesis | 1 | 0,36 | cellular component morphogenesis |
| 7 | positive regulation by organism of defense response of other organism during symbiotic interaction | 1 | 0,36 | positive regulation of defense response, modulation by organism of defense response of other organism during symbiotic interaction |
| 8 | purine ribonucleoside monophosphate biosynthetic process | 1 | 0,36 | purine ribonucleoside monophosphate metabolic process, ribonucleoside monophosphate biosynthetic process, purine ribonucleotide biosynthetic process, purine nucleoside monophosphate biosynthetic process |
| 5 | salicylic acid mediated signaling pathway | 1 | 0,36 | cellular response to salicylic acid stimulus, intracellular signaling pathway |
| 6 | diol catabolic process | 1 | 0,36 | alcohol catabolic process, diol metabolic process |
| 7 | RNA elongation | 1 | 0,36 | RNA metabolic process, transcription, DNA-dependent |
| 5 | regulation of anatomical structure morphogenesis | 1 | 0,36 | anatomical structure morphogenesis, regulation of developmental process |
| 6 | aromatic amino acid family catabolic process | 1 | 0,36 | aromatic amino acid family metabolic process, aromatic compound catabolic process, cellular amino acid catabolic process |
| 8 | modulation by organism of apoptosis in other organism during symbiotic interaction | 1 | 0,36 | regulation of apoptosis, modulation of programmed cell death in other organism during symbiotic interaction |
| 8 | di-, tri-valent inorganic cation homeostasis | 1 | 0,36 | cation homeostasis |
| 5 | monohydric alcohol metabolic process | 1 | 0,36 | alcohol metabolic process |
| 4 | oxygen and reactive oxygen species metabolic process | 1 | 0,36 | cellular metabolic process |
| 5 | indole derivative biosynthetic process | 1 | 0,36 | indole derivative metabolic process, cellular biosynthetic process |
| 8 | IMP metabolic process | 1 | 0,36 | purine ribonucleoside monophosphate metabolic process |
| 6 | modulation by symbiont of host protein levels | 1 | 0,36 | modification of morphology or physiology of other organism during symbiotic interaction |
| 7 | pyrimidine deoxyribonucleoside triphosphate metabolic process | 1 | 0,36 | deoxyribonucleoside triphosphate metabolic process, pyrimidine nucleoside triphosphate metabolic process, pyrimidine deoxyribonucleoside metabolic process, pyrimidine deoxyribonucleotide metabolic process |
| 8 | modulation by organism of jasmonic acid-mediated defense response of other organism during symbiotic interaction | 1 | 0,36 | modulation by organism of immune response of other organism during symbiotic interaction |
| 7 | aerobic respiration | 1 | 0,36 | cellular respiration |
| 8 | transcription from RNA polymerase II promoter | 1 | 0,36 | transcription, DNA-dependent |
| 3 | organic substance metabolic process | 1 | 0,36 | metabolic process |
| 6 | aminoglycan catabolic process | 1 | 0,36 | aminoglycan metabolic process, polysaccharide catabolic process |
| 6 | negative regulation of cell death | 1 | 0,35 | negative regulation of cellular process, cell death, regulation of cell death |
| 6 | cellular respiration | 2 | 0,35 | energy derivation by oxidation of organic compounds |
| 8 | nucleoside monophosphate metabolic process | 3 | 0,34 | nucleotide metabolic process |
| 3 | anatomical structure development | 2 | 0,29 | developmental process |
| 6 | response to immune response of other organism during symbiotic interaction | 2 | 0,29 | response to defenses of other organism during symbiotic interaction |
| 8 | deoxyribonucleotide metabolic process | 3 | 0,29 | nucleotide metabolic process |
| 4 | regulation of response to stimulus | 2 | 0,29 | regulation of biological process, response to stimulus |
| 3 | cell communication | 2 | 0,26 | cellular process |
| 4 | organic ether metabolic process | 2 | 0,26 | small molecule metabolic process |
| 6 | negative regulation of immune response | 2 | 0,26 | immune response, negative regulation of immune system process, regulation of immune response, negative regulation of response to stimulus |
| 6 | regulation of programmed cell death | 1 | 0,26 | programmed cell death, regulation of cell death |
| 4 | regulation of immune system process | 2 | 0,25 | regulation of biological process, immune system process |
| 8 | ribonucleoside monophosphate biosynthetic process | 1 | 0,22 | ribonucleoside monophosphate metabolic process, ribonucleotide biosynthetic process, nucleoside monophosphate biosynthetic process |
| 5 | energy derivation by oxidation of organic compounds | 2 | 0,21 | generation of precursor metabolites and energy |
| 5 | regulation of response to biotic stimulus | 1 | 0,22 | response to biotic stimulus, regulation of response to stimulus |
| 4 | regulation of developmental process | 1 | 0,22 | developmental process, regulation of biological process |
| 6 | cellular biogenic amine catabolic process | 1 | 0,22 | cellular amino acid derivative catabolic process, amine catabolic process, cellular biogenic amine metabolic process |
| 6 | coenzyme catabolic process | 1 | 0,22 | cofactor catabolic process, coenzyme metabolic process |
| 6 | cellular response to salicylic acid stimulus | 1 | 0,22 | cellular response to organic substance, response to salicylic acid stimulus |
| 5 | phenol metabolic process | 1 | 0,22 | cellular aromatic compound metabolic process, alcohol metabolic process |
| 6 | indole derivative catabolic process | 1 | 0,22 | indole derivative metabolic process, aromatic compound catabolic process |
| 7 | purine nucleoside monophosphate biosynthetic process | 1 | 0,22 | purine nucleoside monophosphate metabolic process, purine nucleotide biosynthetic process, nucleoside monophosphate biosynthetic process |
| 5 | ribonucleoprotein complex assembly | 1 | 0,22 | ribonucleoprotein complex biogenesis, cellular macromolecular complex assembly |
| 8 | deoxyribonucleoside triphosphate metabolic process | 1 | 0,22 | deoxyribonucleotide metabolic process, nucleoside triphosphate metabolic process |
| 6 | cellular amino acid derivative biosynthetic process | 1 | 0,22 | small molecule biosynthetic process, cellular amino acid derivative metabolic process, cellular biosynthetic process |
| 7 | protein targeting | 1 | 0,22 | intracellular protein transport |
| 7 | modulation by organism of defense-related callose deposition of other organism during symbiotic interaction | 1 | 0,22 | modulation by organism of defense response of other organism during symbiotic interaction |
| 7 | respiratory electron transport chain | 1 | 0,22 | electron transport chain, cellular respiration |
| 5 | cellular ion homeostasis | 1 | 0,22 | ion homeostasis, cellular chemical homeostasis |
| 4 | defense response, incompatible interaction | 1 | 0,22 | response to other organism, innate immune response |
| 7 | purine ribonucleoside monophosphate metabolic process | 1 | 0,22 | purine nucleoside monophosphate metabolic process, ribonucleoside monophosphate metabolic process, purine ribonucleotide metabolic process |
| 7 | modulation by organism of defense-related cell wall thickening in other organism during symbiotic interaction | 1 | 0,22 | modulation by organism of defense response of other organism during symbiotic interaction |
| 4 | intracellular signaling pathway | 1 | 0,22 | signaling pathway |
| 4 | regulation of multi-organism process | 1 | 0,22 | multi-organism process, regulation of biological process |
| 8 | pyrimidine deoxyribonucleoside metabolic process | 1 | 0,22 | deoxyribonucleoside metabolic process, pyrimidine nucleoside metabolic process |
| 5 | cellular response to oxidative stress | 1 | 0,22 | cellular response to stress, cellular response to chemical stimulus, response to oxidative stress |
| 6 | protein localization in organelle | 1 | 0,22 | cellular protein localization |
| 6 | positive regulation of defense response | 1 | 0,22 | defense response, positive regulation of response to stimulus, regulation of defense response |
| 7 | regulation of apoptosis | 1 | 0,22 | regulation of programmed cell death, apoptosis |
| 6 | acetyl-CoA metabolic process | 1 | 0,22 | coenzyme metabolic process |
| 6 | pyrimidine nucleoside triphosphate metabolic process | 1 | 0,22 | nucleoside triphosphate metabolic process, pyrimidine nucleotide metabolic process |
| 6 | regulation of innate immune response | 1 | 0,22 | regulation of immune response, innate immune response, regulation of defense response |
| 5 | cellular component morphogenesis | 1 | 0,22 | cellular developmental process, anatomical structure morphogenesis, cellular component organization |
| 5 | diol metabolic process | 1 | 0,22 | alcohol metabolic process |
| 4 | organelle assembly | 1 | 0,22 | cellular component assembly, organelle organization |
| 6 | positive regulation of immune response | 1 | 0,22 | regulation of immune response, positive regulation of response to stimulus, positive regulation of immune system process, immune response |
| 5 | response to reactive oxygen species | 1 | 0,22 | response to inorganic substance, response to oxidative stress |
| 5 | negative regulation of immune system process | 2 | 0,16 | immune system process, regulation of immune system process, negative regulation of biological process |
| 5 | regulation of cell death | 1 | 0,16 | cell death, regulation of cellular process |
| 6 | deoxyribonucleoside metabolic process | 1 | 0,13 | nucleoside metabolic process |
| 6 | pyrimidine nucleoside metabolic process | 1 | 0,13 | nucleoside metabolic process |
| 5 | positive regulation of response to stimulus | 1 | 0,13 | regulation of response to stimulus, response to stimulus, positive regulation of biological process |
| 5 | response to salicylic acid stimulus | 1 | 0,13 | response to organic substance |
| 3 | signaling pathway | 1 | 0,13 | signaling |
| 5 | positive regulation of immune system process | 1 | 0,13 | immune system process, regulation of immune system process, positive regulation of biological process |
| 5 | cellular response to organic substance | 1 | 0,13 | response to organic substance, cellular response to chemical stimulus |
| 8 | ribonucleoside monophosphate metabolic process | 1 | 0,13 | ribonucleotide metabolic process, nucleoside monophosphate metabolic process |
| 6 | purine nucleoside monophosphate metabolic process | 1 | 0,13 | purine nucleotide metabolic process, nucleoside monophosphate metabolic process |
| 5 | cofactor catabolic process | 1 | 0,13 | cellular catabolic process, cofactor metabolic process |
| 4 | cellular chemical homeostasis | 1 | 0,13 | chemical homeostasis, cellular homeostasis |
| 6 | cellular amino acid derivative catabolic process | 1 | 0,13 | small molecule catabolic process, cellular catabolic process, cellular amino acid derivative metabolic process |
| 6 | intracellular protein transport | 1 | 0,13 | protein transport, cellular protein localization, intracellular transport |
| 5 | cellular protein localization | 1 | 0,08 | cellular macromolecule localization, protein localization |
| 5 | intracellular transport | 1 | 0,08 | establishment of localization in cell, transport |
| 4 | cellular macromolecule localization | 1 | 0,05 | macromolecule localization, cellular localization |
