# Supplementary material for: The mosaicism of plasmids revealed by atypical genes detection and analysis
Source: BMC Genomics. 2011 Aug 8;12:403. doi: 10.1186/1471-2164-12-403 (PMC3166947; doi:10.1186/1471-2164-12-403)
Supplement: Additional file 10 — the atypical mer, tet, maxi and cbi clusters. Schematic representation of the atypical mer, tet, maxi and cbi clusters found in different plasmids of different microorganisms. [file 1471-2164-12-403-S10.PPT]

## Slide 1
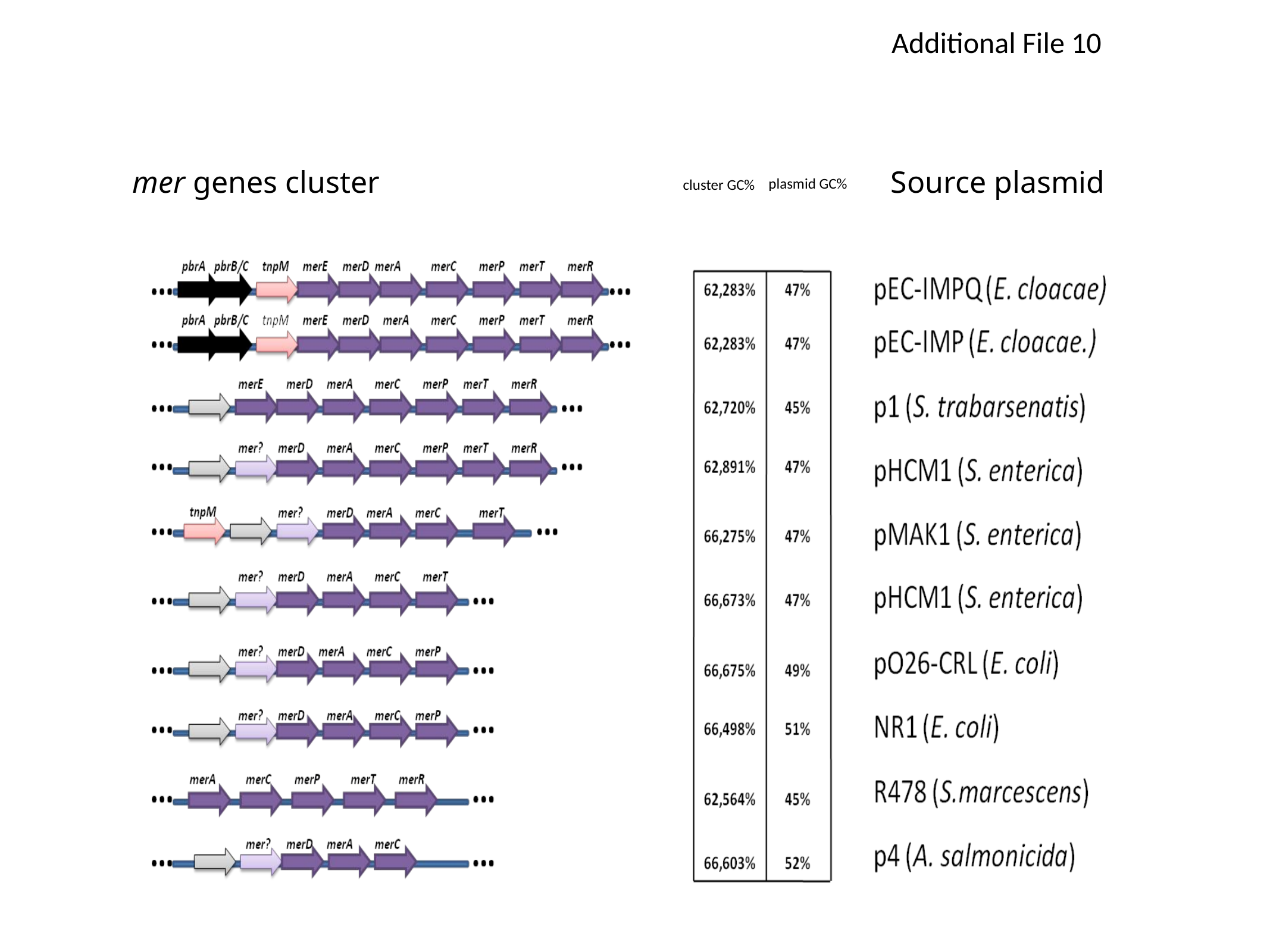

Additional File 10
mer genes cluster
Source plasmid
plasmid GC%
cluster GC%

## Slide 2
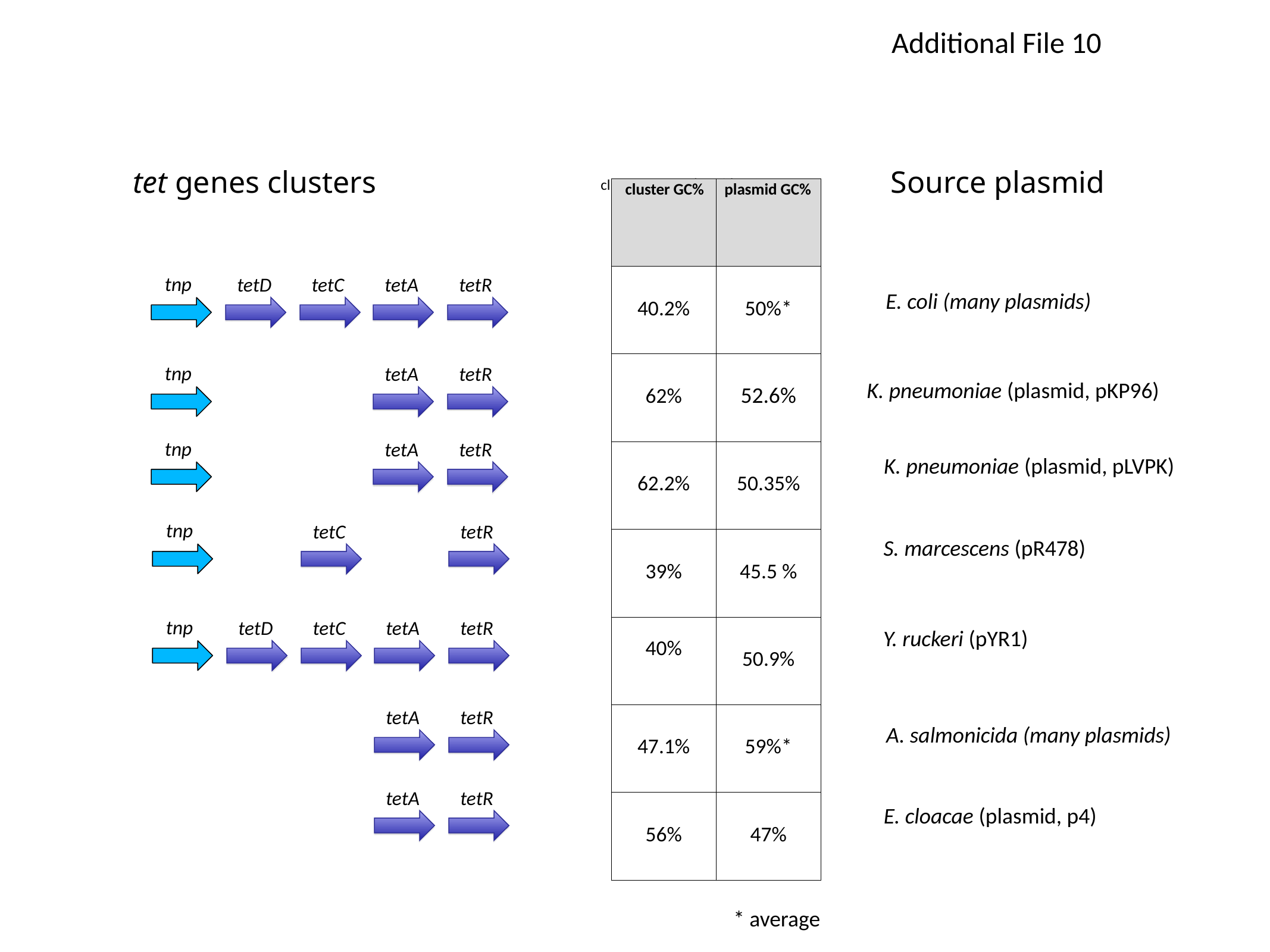

Additional File 10
tet genes clusters
Source plasmid
plasmid GC%
cluster GC%
| cluster GC% | plasmid GC% |
| --- | --- |
| 40.2% | 50%\* |
| 62% | 52.6% |
| 62.2% | 50.35% |
| 39% | 45.5 % |
| 40% | 50.9% |
| 47.1% | 59%\* |
| 56% | 47% |
tnp
tetD
tetC
tetA
tetR
E. coli (many plasmids)
tnp
tetA
tetR
K. pneumoniae (plasmid, pKP96)
tnp
tetA
tetR
K. pneumoniae (plasmid, pLVPK)
tnp
tetC
tetR
S. marcescens (pR478)
tnp
tetD
tetC
tetA
tetR
Y. ruckeri (pYR1)
tetA
tetR
A. salmonicida (many plasmids)
tetA
tetR
E. cloacae (plasmid, p4)
* average

## Slide 3
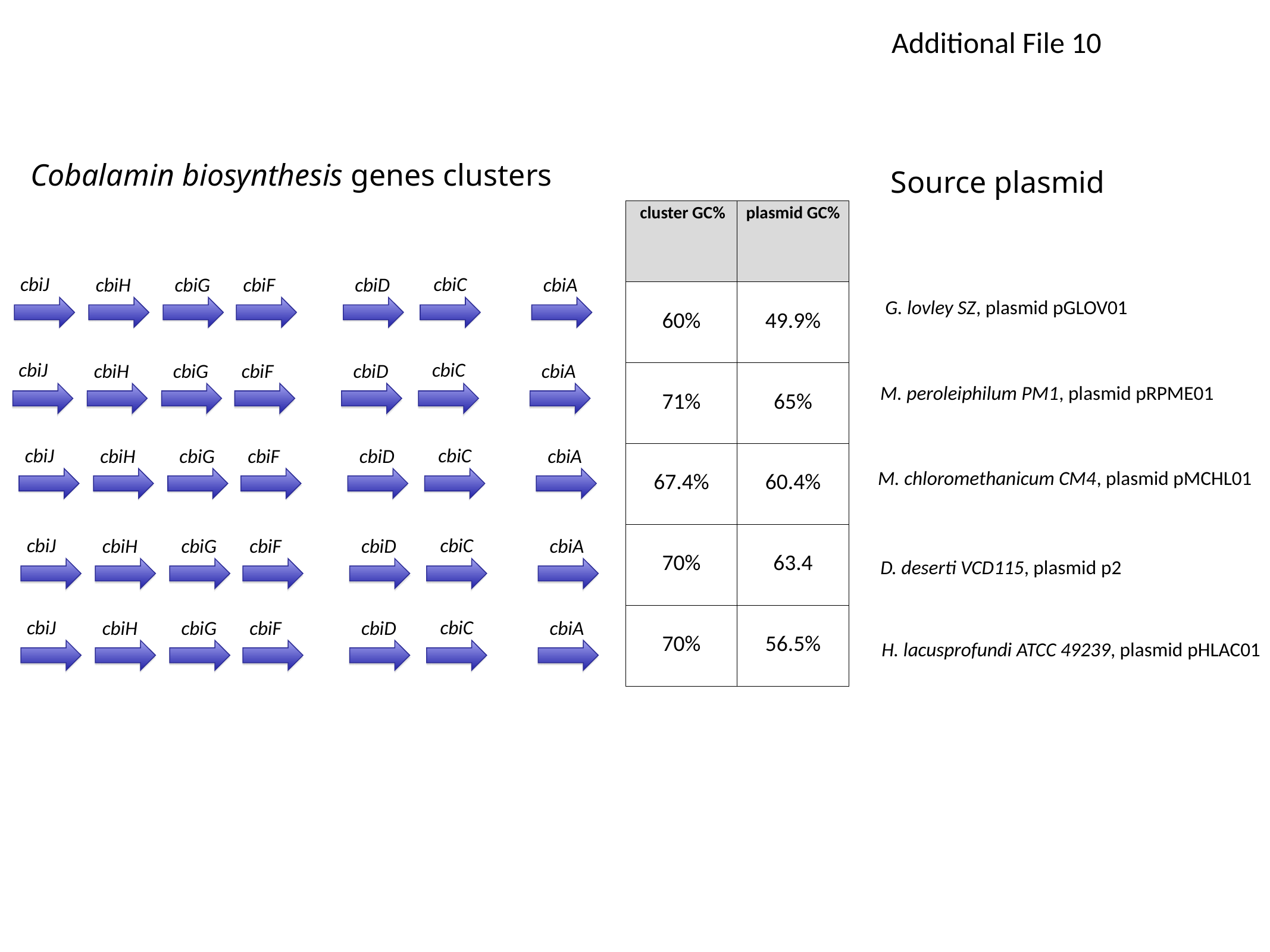

Additional File 10
Cobalamin biosynthesis genes clusters
Source plasmid
| cluster GC% | plasmid GC% |
| --- | --- |
| 60% | 49.9% |
| 71% | 65% |
| 67.4% | 60.4% |
| 70% | 63.4 |
| 70% | 56.5% |
cbiJ
cbiC
cbiH
cbiG
cbiF
cbiD
cbiA
G. lovley SZ, plasmid pGLOV01
cbiJ
cbiC
cbiH
cbiG
cbiF
cbiD
cbiA
M. peroleiphilum PM1, plasmid pRPME01
cbiJ
cbiC
cbiH
cbiG
cbiF
cbiD
cbiA
M. chloromethanicum CM4, plasmid pMCHL01
cbiJ
cbiC
cbiH
cbiG
cbiF
cbiD
cbiA
D. deserti VCD115, plasmid p2
cbiJ
cbiC
cbiH
cbiG
cbiF
cbiD
cbiA
H. lacusprofundi ATCC 49239, plasmid pHLAC01

## Slide 4
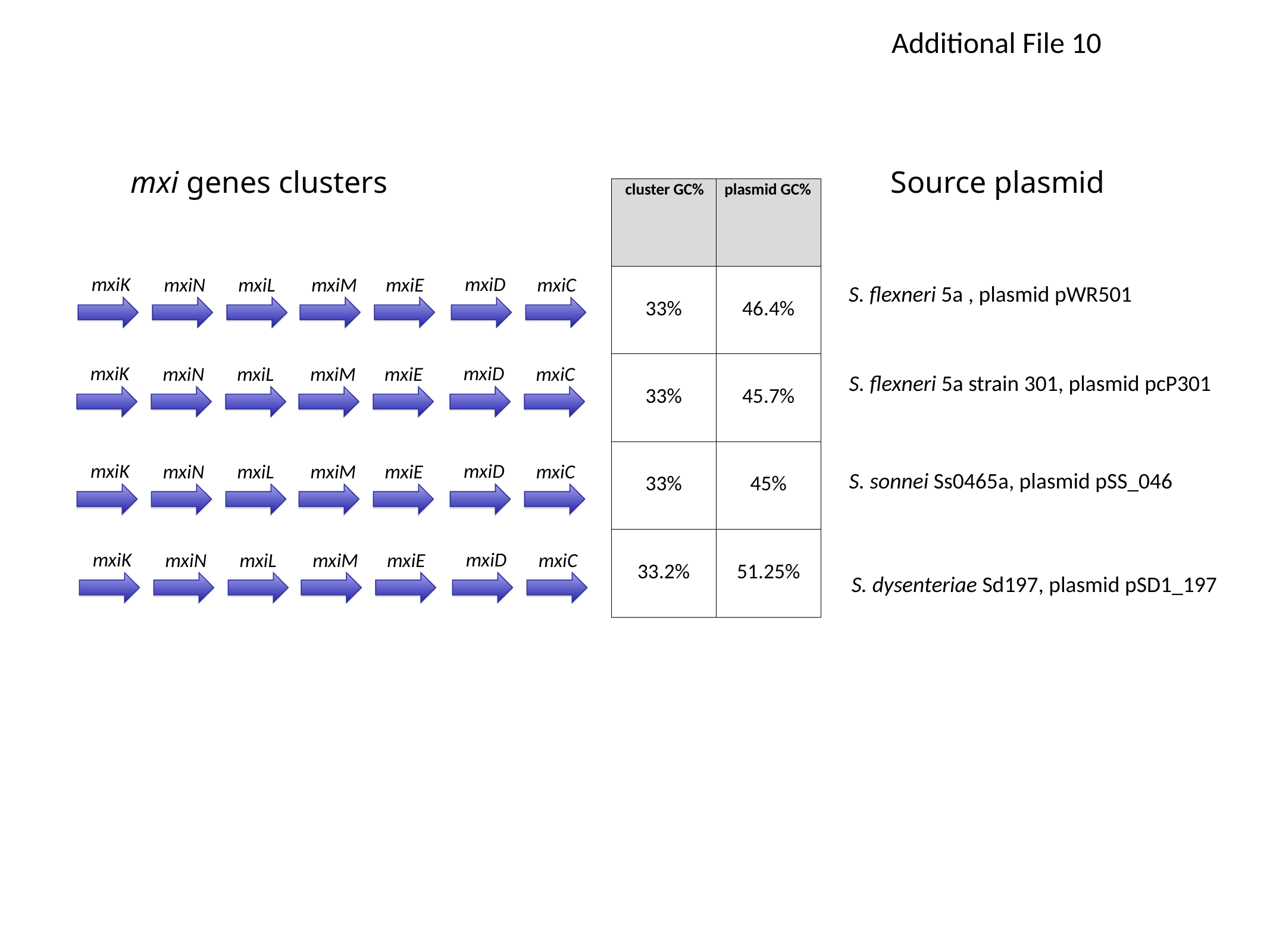

Additional File 10
mxi genes clusters
Source plasmid
| cluster GC% | plasmid GC% |
| --- | --- |
| 33% | 46.4% |
| 33% | 45.7% |
| 33% | 45% |
| 33.2% | 51.25% |
mxiK
mxiD
mxiN
mxiL
mxiM
mxiE
mxiC
S. flexneri 5a , plasmid pWR501
mxiK
mxiD
mxiN
mxiL
mxiM
mxiE
mxiC
S. flexneri 5a strain 301, plasmid pcP301
mxiK
mxiD
mxiN
mxiL
mxiM
mxiE
mxiC
S. sonnei Ss0465a, plasmid pSS_046
mxiK
mxiD
mxiN
mxiL
mxiM
mxiE
mxiC
S. dysenteriae Sd197, plasmid pSD1_197
